# Latent generative search unlocks de novo design of untapped biomolecular interactions at scale

**DOI:** 10.64898/2026.09.12.751118

**Published:** 2026-09-18

**Authors:** Kieran Didi, Danny Reidenbach, Matthew Penner, Supriya Ravichandran, Marshall Case, Mike Nichols, Erik Swanson, Alex Reis, Maggie Prescott, Yue Qian, Dongming Qian, Jingjing Yang, Weiji Li, Le Li, Daichi Shonai, Sean Gay, Bhoomika Basu Mallik, Ho Yeung Chim, Liurong Chen, Miguel Atienza Juanatey, Hubert Klein, Dominic Rieger, Phillip Schlegel, Anna U. Macintyre, Maxim Secor, Daniele Granata, Sooyoung Cha, Zhonglin Cao, Guoqing Zhou, Tomas Geffner, Xi Chen, Micha Livne, Zuobai Zhang, Tianjing Zhang, Kyle Gion, Michael M. Bronstein, Martin Steinegger, Kristine Deibler, Scott Soderling, Clara T. Schoeder, Alena Khmelinskaia, Florian Hollfelder, Christian Dallago, Emine Kucukbenli, Arash Vahdat, Pierce Ogden, Karsten Kreis

**Affiliations:** 1 NVIDIA Corporation, Santa Clara, CA, USA.; 2 University of Oxford, Oxford, UK.; 3 University of Cambridge, Cambridge, UK.; 4 Manifold Bio, Boston, MA, USA.; 5 Viva Biotech (Shanghai) Limited, Shanghai, China.; 6 Duke University, Durham, NC, USA.; 7 Ludwig-Maximilians-Universität München, Munich, Germany.; 8 Leipzig University, Leipzig, Germany.; 9 Novo Nordisk A/S, London, UK.; 10 Novo Nordisk, Lexington, MA, USA.; 11 Novo Nordisk A/S, Copenhagen, Denmark.; 12 Seoul National University, Seoul, South Korea.; 13 Center for Scalable Data Analytics and Artificial Intelligence (ScaDS.AI), Dresden/Leipzig, Germany.; 14 AITHYRA, Vienna, Austria.

**Keywords:** de novo protein design, generative models, protein binders, inference-time search, carbohydrate recognition

## Abstract

De novo protein design has advanced rapidly, yet designing binders to polar, solvent-exposed epitopes and small, flexible ligands remains challenging. Such hydrated surfaces and flexible molecules, including carbohydrates, provide few of the hydrophobic contacts favoured by current methods and have largely resisted de novo binders. To address this challenge, here we introduce latent generative search for binder design, a novel framework that uses reward-guided search at inference time to steer the Proteína-Complexa generative model. The model codesigns sequence and structure — generating them together in a continuous latent space — and thereby removes the inverse-folding step on which current methods rely. In a screen of more than one million designs by multiplexed phage display, latent generative search produced more validated binders than every other method tested, its codesigned sequences surpassing post hoc redesign. It delivered high-affinity binders across therapeutic receptors, a viral attachment protein and intracellular signalling targets. Our approach also accessed previously untapped biology, generating the first de novo proteins that bind a free carbohydrate, including one that discriminates between blood-group antigens — a polar, flexible target class beyond the reach of current design methods.

## 1 Introduction

Across artificial intelligence, the most difficult problems repeatedly led to a common recipe: a generative model that learns the structure of a solution space, paired with a search procedure that explores that space at inference time. Neither component alone offers the same capabilities: the learned prior supplies plausible candidates, while search refines them toward a goal. Search-based methods first achieved landmark successes in constrained environments like board games [1]. More recently, they proved transformative in conjunction with generative models that make challenging, unstructured problems amenable to search, including image generation [2, 3] and language modelling, for instance through test-time reasoning [4, 5].

Designing proteins that bind chosen targets with high affinity and selectivity is a problem of similar difficulty, with broad implications for therapeutics, diagnostics and synthetic biology [6]. Deep learning made it increasingly attainable [7, 8]: protein structure-prediction models provide accurate target structures [9, 10] and filtering metrics [11, 12], while generative methods, such as RFdiffusion [13], or hallucination approaches, such as BindCraft [14], produce candidate backbones. However, the pairing of a generative prior with principled search that has seen success elsewhere has not been realised here. Generative models typically produce a protein structure in a single pass, with no optimisation of the sample, and quality often falls short without extensive downstream filtering [15]. Hallucination methods instead search over sequences without a learned generative prior to guide them, which can make the search brittle and prone to non-physical solutions [16]. Both paradigms also depend on a separate inverse-folding step to assign sequences to backbones, which can degrade electrostatic and other functionally critical interactions [14, 17]. These shortcomings are most acute at polar, solvent-exposed interfaces and against carbohydrates and flexible peptides, where sequence and backbone geometry co-optimisation would enable precise placement of hydrogen bonds — and such targets therefore remain largely out of reach.

**Fig. 1:**
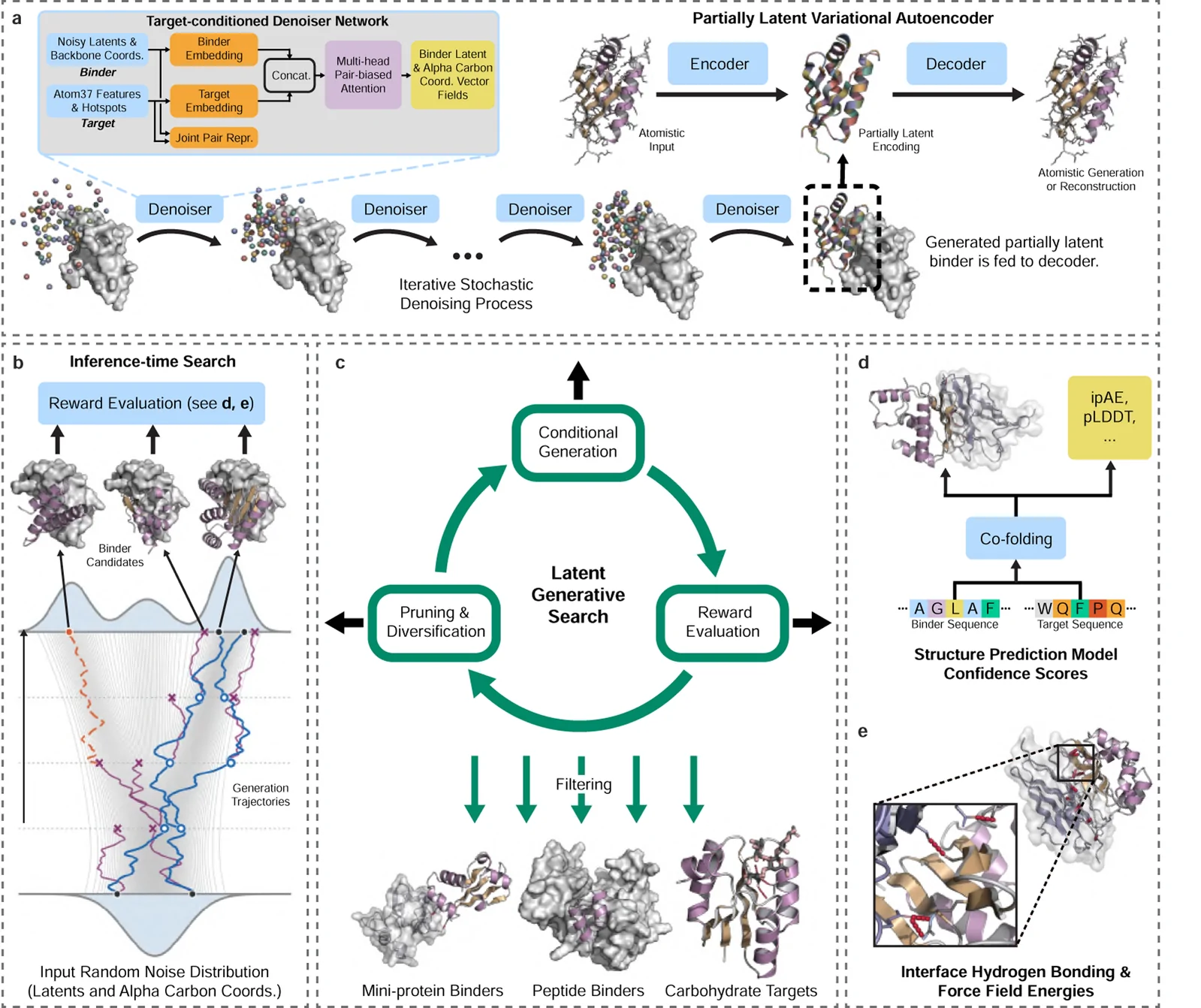
Latent generative search for de novo protein binder design. **a**, The conditional codesign generator, Proteína-Complexa, generates binder structure and sequence together using a partially latent representation [19]. A target-conditioned denoiser network iteratively transforms random noise into backbone coordinates and latent variables, after which a decoder maps the codesigned latent binder representation to a fully atomistic output. An auxiliary encoder is used during training of the variational autoencoder. **b**, Generative sampling trajectories are guided towards high reward regions of the solution space according to task-relevant scoring models in an inference-time search process. Low-scoring trajectories are terminated, whereas promising trajectories are expanded and diversified, for example through beam search. **c**, The complete latent generative search protocol alternates between generative denoising rollouts, reward-based trajectory evaluation, and the subsequent selection and expansion of promising trajectories. The resulting candidates may undergo a final filtering stage using additional scoring functions. The same framework supports diverse design tasks — including monomers, mini-protein binders, peptide binders, and binders to carbohydrate targets — by adapting the conditioning input, reward definition, and scoring models. **d**, Confidence metrics from structure prediction models, including ipAE and pLDDT, can serve as rewards during search or as criteria for final filtering. **e**, Interface hydrogen bonding scores and molecular force fields provide complementary physics-based scoring functions.

Here we bring generative search to protein design, combining a learned protein prior with goal-directed search at inference time beyond filtering. The underlying generative model, Proteína-Complexa, and its inference-time optimization algorithms were introduced previously and shown to be effective for binder design in silico, without wet-lab testing [18]; the model builds on the La-Proteína latent protein representation [19]. We generalise that work into a latent generative search framework for real-world design, defined by application-specific, multi-objective reward functions tailored to each target class, and validate it experimentally at scale across protein–protein, peptide–protein, and protein–carbohydrate interactions (Fig. 1).

Here and throughout, we use *codesign* to mean generating an amino-acid sequence and its all-atom structure together within one generative process, without assigning a new sequence afterward by inverse folding. A *codesigned sequence* is therefore the model’s direct sequence output for its simultaneously generated structure. The framework combines this codesign process with reward-guided search to steer generation toward defined objectives. We evaluate it through biophysical characterisation of designed monomers (Section 3) and initial binder tests against PDGFR, PD-L1, and the Nipah virus G-glycoprotein (Section 4); a benchmark of more than one million designs against 127 diverse and challenging targets (Section 5); nanomolar binders to the muscle-wasting receptor ActRIIA that block signalling in cells (Section 6); peptide and mini-protein kinase binders (Section 7); and, to our knowledge, the first experimentally validated de novo binders to a carbohydrate target, a blood-group antigen (Section 8). Together, these results establish latent generative search as a state-of-the-art framework for protein binder design.

## 2 A latent generative search framework

A central difficulty in atomistic protein generation is that the number of atoms varies with amino-acid identity, complicating the joint modelling of sequence and structure. La-Proteína addresses this with a partially latent representation: backbone C_*α*_ coordinates are modelled explicitly, while each residue’s amino-acid identity and remaining atoms are compressed into a fixed-length continuous latent variable by a variational autoencoder [19]. A flow-matching model then learns the joint distribution over backbone coordinates and latents, so that every codesigned model sample represents a complete protein — backbone, side chains and sequence — without requiring a separate sequence-design step [20].

Proteína-Complexa extends this representation to binder–target complexes by conditioning the flow model on a target structure and optional interface hotspot residues, while the autoencoder continues to encode and decode monomeric binders [18] (Fig. 1a). The model is trained in stages: the autoencoder and partially latent flow model are pre-trained on monomer structures, followed by training of the latent prior on Teddymer — a large synthetic dataset of binder–target pairs derived from domain–domain interactions in AlphaFold Database structures [21, 22] — and experimental multimers from the Protein Data Bank [23]. Because the generator is efficient, generation can be combined with reward-guided search at inference time: rather than accepting or rejecting individual samples, the model repeatedly generates and evaluates multiple stochastic denoising trajectories, selecting and expanding promising trajectories to steer generation towards high-quality binders (Fig. 1b,c; details in Methods Section 10). The same process can partially noise an existing binder and codesign a related backbone and sequence, enabling scaffold-based re-engineering rather than fixed-backbone sequence redesign.

The rewards used in this study encode molecular requirements familiar to biochemists and can be arbitrarily extended. First, hydrogen-bond terms favour correctly placed donor–acceptor interactions and satisfaction of buried polar groups (Fig. 1e) [24, 25]. Second, clash avoidance, shape complementarity, pocket burial and interface packing assess steric and docking compatibility. Third, structure-prediction confidence and Rosetta/Tmol energy terms assess whether the designed protein and complex are likely to fold and remain stable [26, 27]. Fourth, protein-language-model likelihoods favour sequences that are plausible in their local and global context. Fifth, hotspot engagement requires the designed binder to contact specified target residues expected to contribute to binding. Different campaigns combine these molecular terms in different proportions, allowing the same search framework to address protein interfaces, peptides and free carbohydrates.

The latent generative search framework therefore combines complementary advantages: its learned generative prior anchors search in the distribution of plausible proteins, enabling more efficient and robust exploration than prior hallucination methods, while inference-time steering provides greater control over generation than unguided generative approaches. Joint sequence–structure codesign avoids post hoc inverse-folding redesign, which can disrupt functionally important side-chain interactions, while the flexible reward framework adapts the same search process to a broad range of design tasks.

## 3 Designed monomeric proteins

Reliable binder design requires generative models that produce both designable backbones and realistic sequences encoding stable, functional proteins, an end-to-end capability that remains an open experimental challenge. Leading approaches such as RFdiffusion and RFdiffusion2 generate protein geometry and rely on ProteinMPNN or LigandMPNN for the final sequences [13, 28]. Even all-atom, sequence-aware methods often retain post hoc redesign: RFdiffusion3 generates alanine-biased sequences whose sequence–structure consistency improves after ProteinMPNN redesign, while BoltzGen includes a dedicated inverse-folding module to improve predicted refolding and solubility [29, 30]. Because Proteína-Complexa instead codesigns sequence and structure in a single pass, we posed a controlled experimental test: do its codesigned sequences express, fold and remain soluble as well as ProteinMPNN-designed sequences? This comparison tests whether generative codesign can replace inverse folding, rather than only whether the model produces designable backbones. To our knowledge, no generative codesign method had previously demonstrated experimentally that its codesigned sequences perform at parity with a dedicated inverse-folding model.

**Fig. 2:**
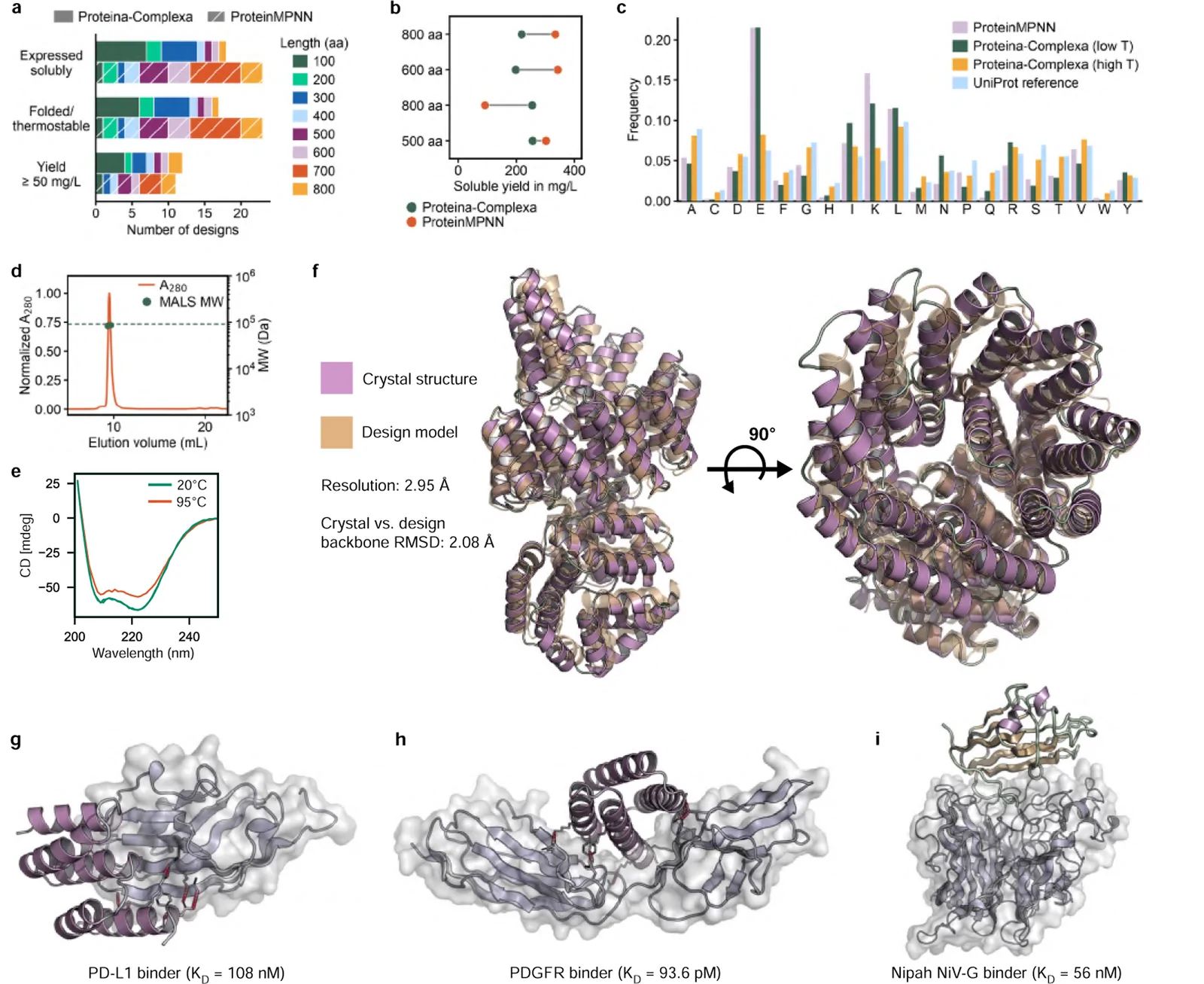
Designed monomers and protein binders. **a**, Outcomes for 48 designed monomers (100–800 aa): soluble expression, thermostable folding and yield, comparing codesigned (Proteína-Complexa) and ProteinMPNN-designed sequences. **b**, Expression yield comparison between Proteína-Complexa codesigned and ProteinMPNN-designed sequences for four shared backbones. **c**, Amino-acid composition of codesigned sequences (Proteína-Complexa, low and high sampling temperature) versus ProteinMPNN-designed sequences and a UniProt reference. **d**, Size-exclusion chromatography (SEC) profile overlaid with the molecular weight determined by multi-angle light scattering (MALS) for a representative 800-residue design. **e**, Circular dichroism (CD) spectra at 20°C and 95°C for the same design. **f**, Crystal structure of the same design (purple) overlaid on its computational model (wheat). The structure was determined at 2.95 Å resolution and resolved 794 of 800 residues; only loop residues 311–316 were unresolved. The backbone RMSD between the resolved experimental structure and the design model is 2.08 Å; per-residue RMSD in Supplementary Fig. S5. **g**, PD-L1 binder in complex; target represented with transparent surface (10.4% hit rate; *K*_D_ 108 nM–3.71 *μ*M). **h**, PDGFR binder in complex (63.5% hit rate; *K*_D_ 93.6 pM–1.34 *μ*M). **i**, De novo Nipah NiV-G binder in complex (*K*_D_ 56 nM).

We characterised 48 designed monomers spanning 100–800 amino acids (Fig. 2a). For 24 designs, sequence and backbone were codesigned; for the other 24, the generated backbones were redesigned using ProteinMPNN. For four backbones shared between the two sets, we characterised both the codesigned and ProteinMPNN-designed sequences, enabling a controlled comparison on identical structures (Fig. 2b); the remaining 20 ProteinMPNN designs had distinct backbones. Under standard *Escherichia coli* expression, 41 of 48 constructs expressed solubly; for the 38 without significant aggregation, size-exclusion chromatography (SEC) with multi-angle light scattering (MALS) confirmed the expected monomeric mass, and circular dichroism (CD) indicated folded, highly thermostable proteins with melting temperatures above 95 °C (Supplementary Figs. S1–S3). Overall, codesigned and ProteinMPNN-designed sequences performed comparably in these analyses, providing, to our knowledge, the first experimental validation of a generative codesign method whose direct sequence outputs match the performance of ProteinMPNN without post hoc inverse folding.

Proteína-Complexa’s sampling temperature provided tunable control over sequence character: lower temperatures yielded ProteinMPNN-like, readily expressible sequences, whereas higher temperatures produced amino-acid distributions closer to those of natural proteins (Fig. 2c). Among 13 high-temperature designs selected for low sequence identity (≤ 40%) and MSA-supported structure predictions, two expressed solubly, and both showed cooperative thermal transitions below 95,°C (Supplementary Fig. S4). These data suggest a temperature-dependent trade-off between the hyperstability typical of conventionally designed proteins and more natural-like sequence composition and thermal behaviour.

To confirm that the generated backbones are physically realizable and not merely high-scoring in silico, we determined a 2.95 Å-resolution crystal structure of a representative 800-residue design, a length at which most design methods fail to yield successful samples. The structure resolved 794 of 800 residues (99.3%), with only loop residues 311–316 unresolved, and matched the computational model with a backbone RMSD of 2.08 Å across the resolved residues (SEC–MALS results and CD spectra for the crystallised design in Fig. 2d,e; structure in Fig. 2f), to our knowledge the first time a generative model has produced such structures and sequences via codesign.

## 4 Protein binders

PDGFR [31, 32] and PD-L1 [12] provided our first direct tests of Proteína-Complexa’s binder-design capabilities. PDGFR signalling drives proliferative and fibrotic disease, and its extracellular domain presents the kind of polar, solvent-exposed surface that contact-driven design has struggled to engage; PD-L1 is a central immune-checkpoint target in oncology and a common benchmarking target for binder-design pipelines. Interface hotspots were identified by Rosetta alanine scanning of known complexes, and several hotspot combinations were evaluated per target rather than committing to a single in-silico prediction. For each combination, 1,500 candidates were generated by beam search with an AlphaFold2 interface-pAE reward and passed through a two-stage filter on physicochemical and confidence metrics followed by structural validation (Methods); 192 designs per target were then expressed and tested. Of these, 191 (PDGFR) and 192 (PD-L1) expressed and purified, and surface plasmon resonance identified 122 designs with detectable binding to PDGFR and 20 to PD-L1. Measured affinities spanned 93.6 pM to 1.34 *μ*M for PDGFR and 108 nM to 3.71 *μ*M for PD-L1, corresponding to hit rates of 63.5% and 10.4%, thereby experimentally validating Proteína-Complexa’s binder-design capabilities within the latent generative search framework with sequence-structure codesign. Representative PD-L1 and PDGFR complexes are shown in Fig. 2g,h.

Having established binder capability on PDGFR and PD-L1, we turned to the Nipah virus attachment glycoprotein (NiV-G) as a harder test case and a use case for scaffold-based re-engineering. NiV-G is a World Health Organization priority pathogen target with no approved therapeutics, and its receptor-binding site is recessed and difficult to engage [33]. We tested two modes within the same framework. The first was fully de novo design, guided by a multi-objective reward combining Boltz-2 interface confidence with shape complementarity, interface hydrophobicity and hydrogen-bond satisfaction [34]. The second was codesign-based scaffold re-engineering, in which an existing binder is partially noised and a related backbone and sequence are codesigned; this capability follows naturally from generating sequence and structure together and contrasts with fixed-backbone redesign, which can only change the sequence. All 20 designs were expressed, purified and screened by surface plasmon resonance: the de novo set yielded a binder at *K*_D_ = 56 nM, and five of six re-engineered designs bound with nanomolar affinity, all targeting the receptor-binding site (Fig. 2i). The same framework therefore supports both fully de novo design and the optimisation of existing binders without any change of method.

## 5 Benchmarking design methods at scale

The individual campaigns above show that our Proteína-Complexa-based latent generative search framework produces folded proteins and high-affinity binders on selected targets. We next asked how it behaves at scale and how it compares with other methods under matched computational resources. Binder design methods are typically reported on a handful of targets, often with bespoke filtering and compute budgets, which makes head-to-head comparisons difficult and can overstate generality. We therefore designed a benchmark large enough to compare methods under standardized conditions and to probe specificity directly. A 127-target panel was assembled from targets used in publicly reported binder-design studies and benchmarks, including those associated with RFdiffusion, BindCraft and mBER, maximizing overlap with established test cases while spanning diverse protein classes and surface geometries [13, 14, 35].

In total, we screened 1,015,870 designed sequences, generated across more than 1,000 design tasks, against 127 targets by multiplexed phage display. The screened library comprised three non-overlapping subsets dedicated to distinct analyses: 492,636 Proteína-Complexa sequences spanning multiple hotspot combinations for panel-wide target and epitope coverage; 390,569 sequences from all evaluated method and sequence-design configurations for a compute-matched comparison; and 132,665 additional Proteína-Complexa sequences generated at alternative sampling temperatures. All three subsets were assayed in a single experimental campaign.

Each Proteína-Complexa target–hotspot task used standard beam search guided by an AlphaFold2 interface-pAE reward, followed by confidence, clash and structural-diversity filtering (Supplementary Fig. S6). Crucially, the multiplexed format resolves both on-target activity and off-target engagement across the whole panel simultaneously, providing a view of specificity, rather than on-target activity alone, that is inaccessible in small-scale studies where each binder is tested against its intended target alone. Generation and in silico filtering across the campaign required more than 140„000 GPU hours, demonstrating the deployment of the framework at a large computational scale.

**Fig. 3:**
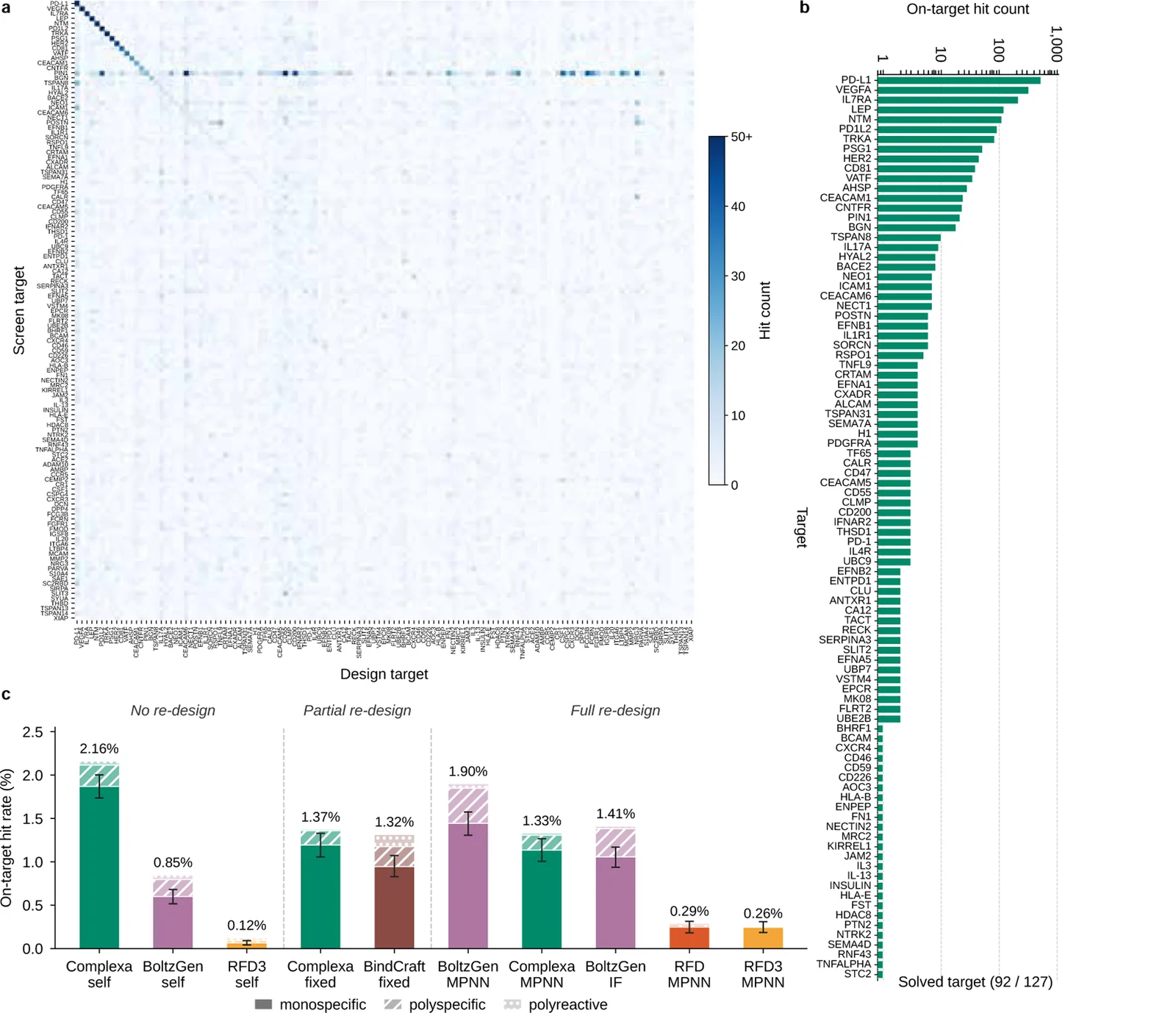
Benchmarking designs at scale against 127 targets by multiplexed phage display. **a**, Cross-reactivity matrix of hit counts for 492,636 Proteína-Complexa-generated sequences (design target, columns) screened against all 127 targets (screen target, rows); on-target hits lie on the diagonal and off-target (cross-reactive) hits off it. **b**, On-target specific hit count for each target, ranked; 92 of 127 targets were solved (at least one on-target specific hit). **c**, On-target specific hit rate across design methods (Proteína-Complexa with latent generative search, BoltzGen, RFdiffusion3, BindCraft, RFdiffusion) and sequence design strategies for the matched-compute comparison sequence set, grouped by native model output (no post hoc redesign), partial redesign (fixed-interface) and full redesign (ProteinMPNN/inverse folding); bars are split into monospecific, poly-specific and polyreactive fractions.

High-throughput screening of the 492,636 Proteína-Complexa-generated sequence subset across the 127-target panel with diverse epitopes revealed broad target coverage, with binding events strongly enriched toward the intended targets (Fig. 3a). Proteína-Complexa produced at least one on-target hit for 115 of 127 targets (91%); 92 targets (72%, Fig. 3b) yielded target-specific binders and 78 yielded poly-specific binders engaging two to four targets; these categories were not mutually exclusive. Counting each binder once, 1,982 of 23,966 binders with detected activity (8.3%) engaged their intended target, a 10.5-fold enrichment over the 0.79% expected under uniform binding across the panel. Despite this on-target enrichment, off-target binding was also widespread, with every panel target specifically bound by at least one sequence designed against another target, indicating that a single design campaign can yield binding activity well beyond the intended targets. The choice of interface hotspot used to condition generation had a substantial effect on success, and the computationally top-ranked hotspot did not always give the highest experimental hit rate (Supplementary Fig. S7), supporting the evaluation of several hotspot combinations per target rather than a single in silico prediction. The results demonstrate the effectiveness of latent generative search for binder design at scale across diverse targets.

We then compared methods by specific on-target hit rate in a compute-matched analysis restricted to the top-ranked in silico hotspot for each of the 83 targets for which at least one method produced an on-target hit (Fig. 3c). This analysis used the dedicated subset of 390,569 sequences comprising designs from all approaches. For each target, every method was assigned the same candidate budget, and generation was performed under comparable computational budgets. Using codesigned sequences, Proteína-Complexa achieved a specific hit rate of 1.87%, exceeding both the next-best method with native model sequences, BoltzGen (0.60%), and the best method with fully redesigned sequences, BoltzGen with ProteinMPNN (1.44%) [30, 36]. In absolute terms, Proteína-Complexa yielded 702 on-target hits, of which 607 were specific, from 32,469 codesigned sequences. The corresponding on-target hit counts (specific hits in parentheses) were 511 (388) for BoltzGen with ProteinMPNN, 323 (232) for BindCraft [14], 61 (57) for RFdiffusion3 [29] and 61 (52) for RFdiffusion [13].

The sequence-design strategy mattered as much as the backbone-generation method. For Proteína-Complexa, codesigned sequences (702 on-target hits, 607 specific; 1.87% specific hit rate) outperformed both ProteinMPNN redesign (323 hits, 275 specific; 1.13%) and fixed-interface redesign (333 hits, 289 specific; 1.19%) on the same backbones. This ordering did not hold for the other methods: BoltzGen performed best with ProteinMPNN redesign (1.44%) rather than its native sequences (0.60%), and RFdiffusion3 native sequences reached only 0.07%. In this benchmark, Proteína-Complexa with our latent generative search framework was therefore the only method whose codesigned sequences consistently exceeded post hoc redesign strategies, indicating that codesign can reduce reliance on separate inverse-folding models at scale. In silico confidence metrics were predictive of experimental success, with hits enriched among designs with high interface confidence and pLDDT (Supplementary Fig. S8). Finally, sampling temperature ablations incorporating the remaining subset of 132,665 sequences revealed complementary, target-dependent performance across settings, supporting the use of multiple temperatures to broaden target coverage (Supplementary Table S1).

## 6 Binders to a muscle-wasting receptor

**Fig. 4:**
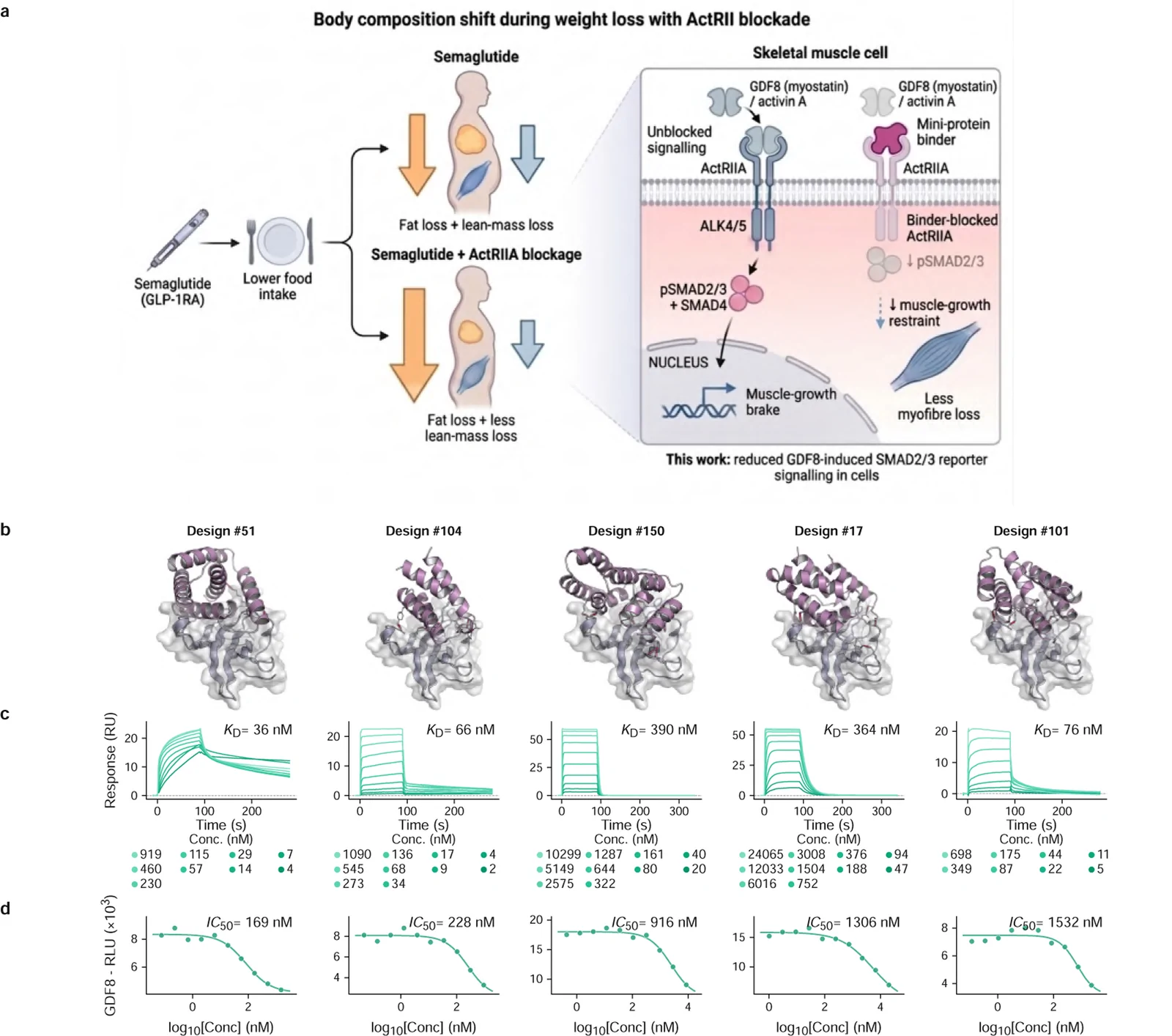
De novo binders to the muscle-wasting receptor ActRIIA. **a**, Rationale for ActRIIA blockade during semaglutide-induced weight loss and schematic of binder-mediated inhibition of GDF8/Activin signalling through Smad2/3. **b**, Predicted structures of five representative designs (#51, #104, #150, #17 and #101; purple) bound to ActRIIA (grey); the binders share a helical binding mode at low pairwise sequence identity (6–18%). **c**, Surface plasmon resonance sensorgrams (analyte concentration series, legends in nM) with fitted *K*_D_ (36–390 nM; tightest design 36 nM). **d**, Inhibition of GDF8 (myostatin)-induced Smad2/3 luciferase signalling in cells, shown as GDF8-driven luminescence versus binder concentration, with fitted IC_50_ (169–1,532 nM).

Activin receptor type IIA (ActRIIA) suppresses skeletal-muscle growth through Smad2/3 signalling and is a target in muscle-wasting disorders, including the lean-mass loss that can accompany the GLP-1 receptor agonist therapies now in widespread use [37]. A designed mini-protein that blocks this receptor could help preserve muscle during weight loss, but doing so demands both high affinity and the ability to interrupt downstream signalling in living cells (Fig. 4a).

Across seven *in silico* design rounds we varied sampling size, hotspot conditioning and the search algorithm — beam search, Feynman–Kac steering or Monte Carlo tree search. Hotspots were drawn from the ActRIIA–bimagrumab and ActRIIA–Activin A interfaces, and candidates were ranked by an averaged complex interface score before scaffold clustering. Of 200 selected designs, 192 expressed and purified. Surface plasmon resonance identified 16 binders, 8 with sub-micromolar affinity and a tightest design at *K*_D_ = 36 nM (Fig. 4c). The sub-micromolar hits shared low sequence identity (6–18%) despite a common helical binding mode (Fig. 4b), again pointing to diverse solutions rather than a single convergent design. Crucially, function tracked affinity: in a Smad2/3 luciferase reporter assay in HEK293 cells, designs inhibited myostatin-induced signalling with half-maximal inhibitory concentrations from 169 to 1,532 nM (Fig. 4d). That computationally designed mini-proteins disrupt receptor signalling in cells — directly from design and without any experimental affinity maturation — shows that the latent generative search framework yields not just binders but functional antagonists of a therapeutically important receptor.

## 7 Peptide and mini-protein kinase binders

**Fig. 5:**
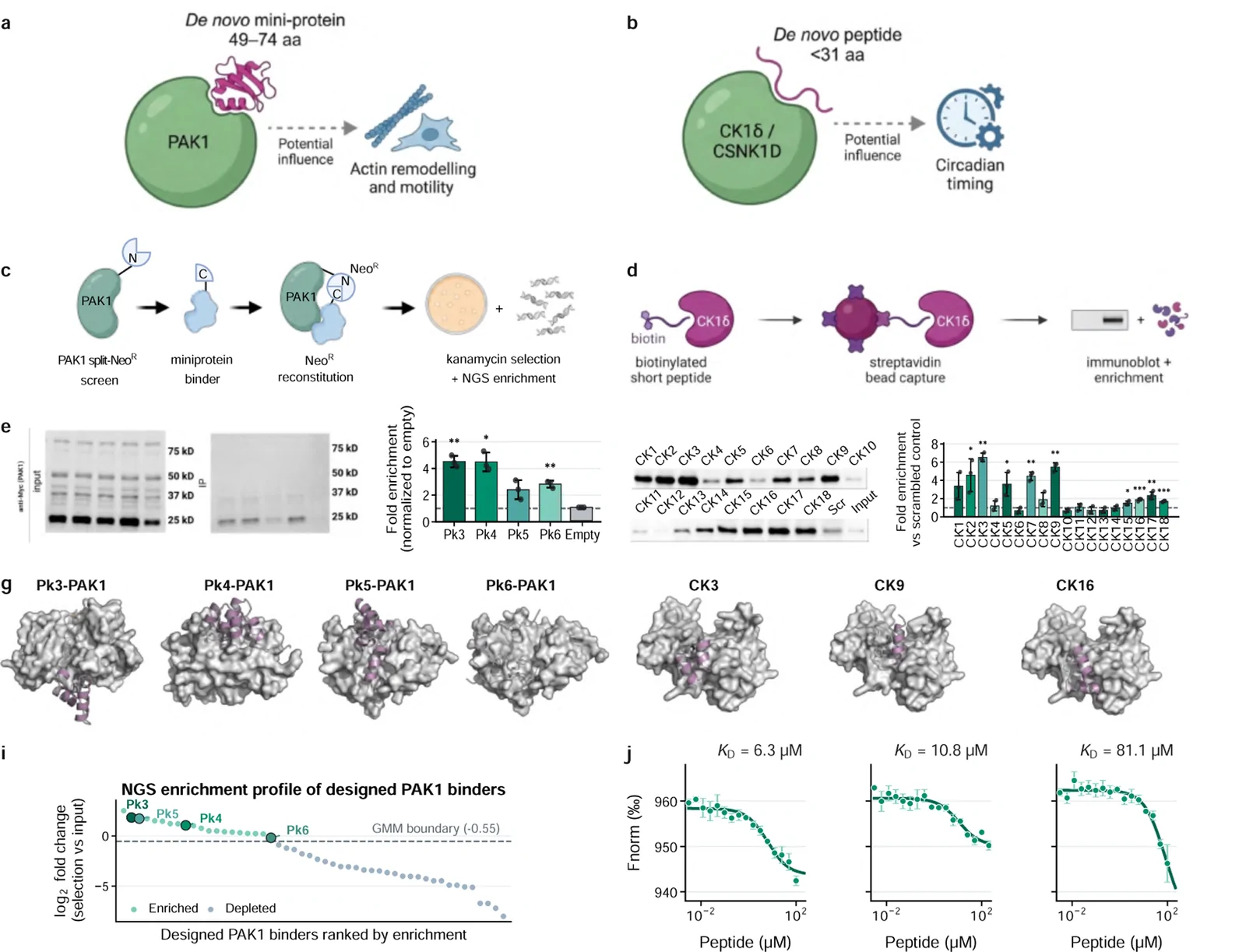
De novo peptide and mini-protein kinase binders. The left column concerns PAK1 mini-proteins (**a**,**c**,**e**,**g**,**i**) and the right column CK1*δ* peptides (**b**,**d**,**f**,**h**,**j**). **a**, Signalling context for PAK1, a Rho-family GTPase effector, and the 49–74-aa mini-protein design regime. **b**, Signalling context for CK1*δ*, a constitutive Ser/Thr kinase, and the <31-aa peptide design regime. **c**, Split-neomycin-resistance complementation assay for PAK1 mini-proteins, read out by next-generation sequencing (NGS). **d**, Streptavidin-bead pull-down assay for biotinylated CK1*δ* peptides. **e**, Mammalian validation of PAK1 binders by representative anti-Myc Western blot and fold enrichment relative to empty vector (mean ± SD, *n* = 3). **f**, CK1*δ* pull-down blots and binding enrichment for CK1–CK18 relative to scrambled control (mean ± SD, *n* = 3–4); 9 of 18 are significant (*p <* 0.05). **g**, AlphaFold2-Multimer predicted PAK1 complexes for Pk3–Pk6 (PAK1 surface, binder cartoon); details in Supplementary Table S2. **h**, Predicted CK3, CK9 and CK16 peptide–CK1*δ* complexes. **i**, Split-NeoR NGS enrichment profile for the 50 PAK1 designs, ranked by fold change, with the Gaussian-mixture decision boundary and Pk3–Pk6 indicated. **j**, Microscale-thermophoresis dose– response curves for CK3, CK9 and CK16 (*K*_D_ = 6.3, 10.8 and 81.1 *μ*M, respectively; mean ± SEM, *n* = 3–4).

Most de novo binder design has focused on mini-proteins of roughly 50–100 residues, where a folded scaffold provides a rigid, preorganised interface. Shorter peptides are both biologically and therapeutically attractive — they are synthetically accessible and can reach buried or constrained sites — but they are far harder to design: with fewer residues and a smaller, more flexible interface, sequence and backbone geometry must be specified together with little tolerance for error, exactly the regime in which codesign should be beneficial. To test generality across this size spectrum, we designed binders to the catalytic domains of two kinases: mini-protein binders (49–74 amino acids) for PAK1 [38, 39] and short peptide binders (<31 amino acids) for CK1*δ* [40, 41] (Fig. 5a,b), choosing different length windows for both targets to probe the capabilities of the method in different scenarios.

For PAK1, 50 mini-protein candidates were generated by beam search with an AlphaFold2 interface-pAE reward and screened by a split-neomycin-resistance complementation assay in *E. coli* [42, 43] (Fig. 5c). Next-generation sequencing classified 20 of 50 designs (40%) as enriched (Fig. 5i), and co-immunoprecipitation in HEK293T cells confirmed binding for top candidates, with the strongest designs reaching roughly 4.5-fold enrichment over control (*P <* 0.01; Fig. 5e). The corresponding predicted PAK1 complexes show distinct binding modes (Fig. 5g). For CK1*δ*, 18 synthesised peptides were assayed by streptavidin pull-down against the purified kinase (Fig. 5d); 9 of 18 (50%) showed significant enrichment over a scrambled control (*P <* 0.05), with the strongest binders exceeding five-fold enrichment (Fig. 5f); all binders in Supplementary Table S3. Predicted CK3, CK9 and CK16 complexes are shown in Fig. 5h, for which we measured low-to-mid micromolar dissociation constants by microscale thermophoresis (Fig. 5j). High success rates in both regimes indicate that codesign generalises across both peptide and protein binders. The binding affinities of CK3, CK9, and CK16 for CK1*δ* were determined by microscale thermophoresis using a Monolith X instrument (NanoTemper Technologies). Normalized fluorescence (*F*_norm_) was measured across a 16-point serial dilution series and fitted to a three-parameter sigmoidal binding model to derive dissociation constants (*K*_d_; Fig. 8e). CK3 exhibited a *K*_d_ of 6.27 ± 1.81 *μ*M, CK9 a *K*_d_ of 10.78 ± 2.83 *μ*M, and CK16 a *K*_d_ of 81.06 ± 31.48 *μ*M (mean ± SD). Together, these data demonstrate that Proteina-Complexa-predicted peptide binders engage the CK1*δ* kinase domain with low-to-mid-micromolar affinity.

## 8 De novo carbohydrate-antigen binders

**Fig. 6:**
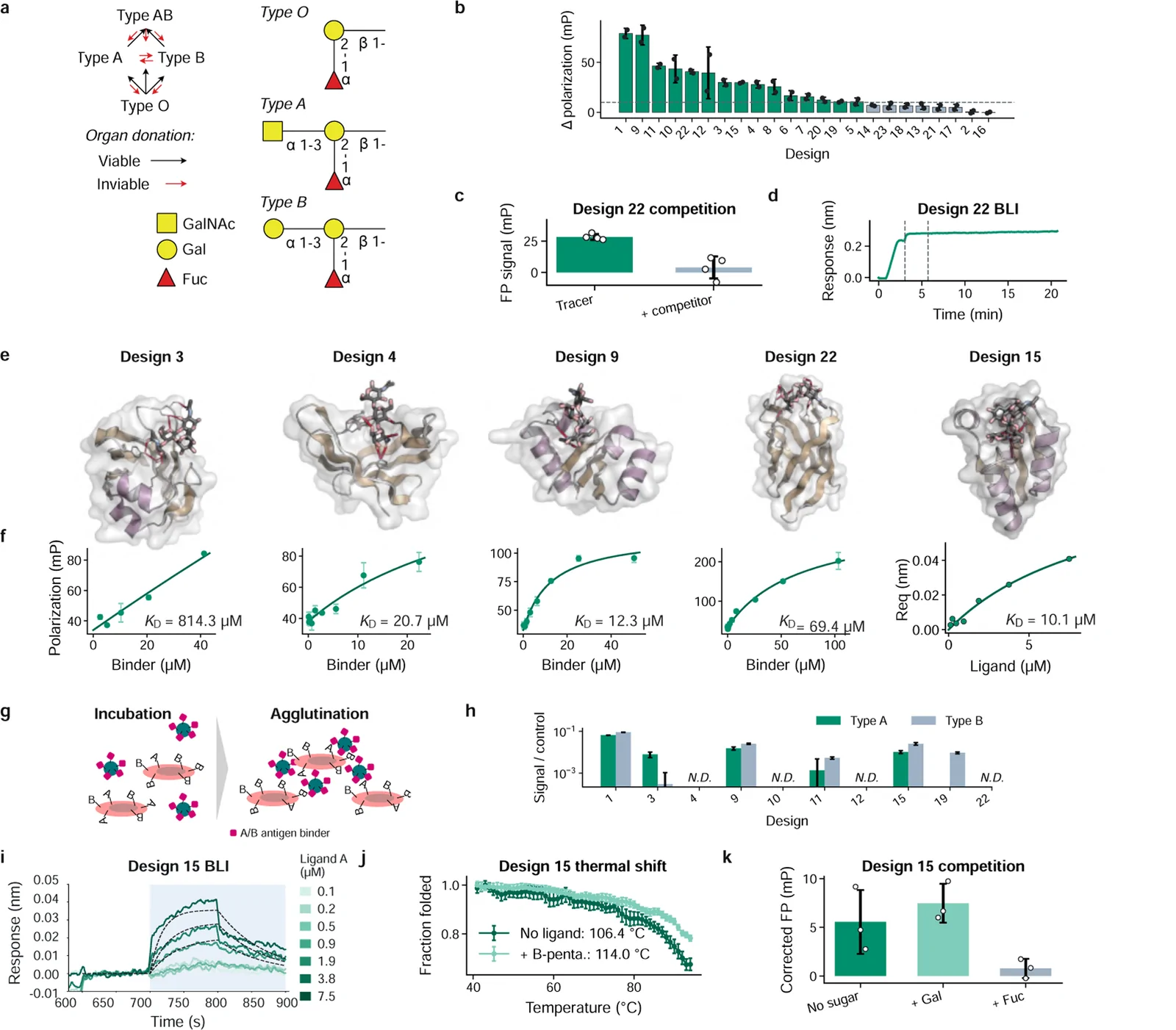
De novo binders that recognise a blood-group carbohydrate antigen. **a**, Principle of ABO blood-group compatibility and schematic structures of the A, B and O(H) antigens; the A antigen terminates in N-acetylgalactosamine (GalNAc), whereas the B antigen terminates in galactose (Gal) (glycan symbols per standard glycomics nomenclature). **b**, FP screen of designs 1–23 against fluorescently labelled type II A pentasaccharide. Δpolarization is relative to the tracer-only control; bars show duplicate means and error bars show s.d. Fifteen designs pass the +10 mP threshold. **c**, Excess unlabelled type II A competes design 22 binding to the fluorescent probe back to baseline. **d**, BLI trace of design 22 against immobilised biotinylated type II A glycan, with association and dissociation phases indicated. **e**, Predicted glycan-bound structures of designs 3, 4, 9, 15 and 22. **f**, Concentration-dependent binding of the same five designs, measured by FP (designs 3, 4, 9 and 22) or BLI equilibrium response (design 15), with fitted *K*_D_; the design 3 estimate lies above the measured concentration range. **g**, Agglutination assay in which His_6_-tagged binders are immobilised on Ni-NTA agarose beads to confer multivalency before incubation with red blood cells. **h**, Type A_2_ and type B red-blood-cell agglutination relative to blank-bead controls (error bars, s.d. of triplicate wells; N.D., no agglutination above the negative control). Design 3 agglutinates A_2_ but not B. **i**, BLI of immobilised design 15 against a type II A pentasaccharide–DNA conjugate, reference-subtracted against a buffer-only control. **j**, Circular-dichroism thermal shift of design 15 with and without type B pentasaccharide. **k**, FP competition for design 15: 30 mM unlabelled D-galactose does not compete, whereas 250 *μ*M L-fucose abolishes the signal (error bars, s.d. of triplicate measurements).

Carbohydrates carry much of the information at the human cell surface — governing blood-group identity, host–pathogen defence and immune signalling — yet they remain almost untouched by de novo design. The ABO system is the canonical example: the A antigen terminates in N-acetylgalactosamine (GalNAc), whereas the B antigen terminates in galactose (Gal), and this single chemical difference is a primary determinant of blood-transfusion and organ-transplant compatibility [44]. In reverse blood typing, patient red blood cells are mixed with antibodies of known specificity and antigen-dependent agglutination reveals the blood group. These antibodies are exquisitely specific but large (>150 kDa), thermally fragile and costly to produce. Smaller carbohydrate-binding molecules bind both antigens without sufficient discrimination, typically with only tens-to-hundreds-of-micromolar affinity, and often require structural metal ions, making them poor typing reagents. A small, hyperstable protein that binds one antigen specifically would enable cheap, cold-chain-free blood-typing reagents for point-of-care and field use, and more broadly a route to programmable carbohydrate recognition. Designing such a binder, however, requires recognition of a small, flexible, densely polar sugar — a hydroxyl-rich surface with little hydrophobic character and a substantial desolvation penalty — such that even natural lectins bind free sugars only weakly [45, 46]. These are exactly the properties that defeat contact-driven design and ProteinMPNN [12]; instead, sequence and backbone geometry must be co-optimised to place a hydrogen-bond network precisely around the ligand. We are not aware of a prior de novo protein that binds a free carbohydrate with measurable affinity (Fig. 6a).

Because carbohydrate recognition depends on precise polar contacts while sugars remain conformationally flexible, we adapted latent generative search to address both properties explicitly. We conditioned Proteína-Complexa on the type II A pentasaccharide and used pocket-burial terms together with physics-based Tmol hydrogen-bond rewards during search to favour geometrically precise polar interactions [25]. At every search step, RosettaFold3 (RF3) predicted the bound complex, and the RF3-predicted sugar conformation was fed back into the next generative step. The search therefore updated the ligand pose as the binder evolved, accounting for binding-dependent glycan flexibility rather than treating the sugar as rigid [27].

We expressed 23 designs, each smaller than 10 kDa, and screened the purified proteins against a fluorescently labelled type II A pentasaccharide by fluorescence polarization (FP). Fifteen designs exceeded a +10 mP threshold (Fig. 6b). Excess unlabelled pentasaccharide competed the FP signal back to baseline (Fig. 6c), and biolayer interferometry (BLI) against immobilised glycan, referenced against non-specific sensor binding, showed a clear on-rate and a very slow off-rate, confirming glycan binding independently of the fluorescent tracer (Fig. 6d). Concentration-series measurements were obtained for ten putative binders and yielded five well-constrained dissociation constants of approximately 12–340 *μ*M in the initial FP analysis. Predicted complexes illustrate the pockets formed around the glycan (Fig. 6e). Five representative designs are shown in Fig. 6f: designs 3, 4, 9 and 22 measured by FP and design 15 measured by BLI equilibrium response. Their fitted *K*_D_ values span 10.1–814.3 *μ*M; the design 3 value is an extrapolated estimate because the fitted *K*_D_ lies above the measured concentration range.

To ask whether these binders can type red blood cells, we conferred multivalency on the His-tagged designs by immobilising them on Ni-NTA beads and measured agglutination of type A_2_ or type B cells by OD_600_ (Fig. 6g). Design 3 agglutinated type A_2_ cells but not type B, discriminating between the two antigens (Fig. 6h); this is a demanding test, because A_2_ cells display the A antigen at low density while the type B cells present a competing, higher-valency surface. Five further designs (1, 9, 11, 15 and 19) agglutinated type A and/or type B cells without a clear preference.

We used design 15, which agglutinated both cell types, as a case study in poly-specificity. It bound the A antigen by BLI (Fig. 6i) and was thermally stabilised by the B pentasaccharide (Fig. 6j), pointing to recognition of a feature shared by both antigens. Competition experiments localised that feature: free galactose (30 mM) did not inhibit FP, whereas L-fucose (250 *μ*M) abolished it (Fig. 6k), and the model predicts a hydrophobic stacking contact between design 15 and the fucose ring. Because fucose is common to the A, B and O antigens, this off-target interaction with a shared monosaccharide compresses the dynamic range of the cell assay — a reminder that screening in the relevant cell type is essential to reveal biologically important off-target effects.

Design 3, by contrast, distinguishes A_2_ from B. Its model places a tyrosine on a *β*-strand to hydrogen-bond the terminal N-acetyl group unique to the A antigen — an interaction that cannot form with the galactose of the B antigen — providing a structural rationale for the observed specificity, and it contacts fucose through only two hydrogen bonds rather than a stacking interaction. Design 3 has no detectable sequence homologue in the non-redundant database and no structural relative by Foldseek (no hit at E < 10^−2^) [47], indicating that Proteína-Complexa reached a region of sequence and fold space without a known natural non-antibody solution to A/B discrimination. A complementary BLI screen of the FP-positive designs against type-A and type-B glycans is consistent with antigen-preferential binding (Supplementary Fig. S9).

## 9 Discussion

The results show that codesign with reward-guided search in a continuous latent space produces functional binders across a wide range of targets and scales. A large-scale screening campaign testing over a million binder candidates demonstrated broad on-target success across a diverse panel of 127 targets. In what is, to our knowledge, the largest experimentally validated benchmark of protein binder design methods to date, our codesign-based latent generative search framework outperformed competing generative models, an optimisation baseline and post hoc redesign under compute-matched conditions, with the advantage over sequence redesign indicating that generating backbone and sequence together yields measurable experimental gains. Individual campaigns further demonstrate the breadth of the approach across modalities: picomolar binders to PDGFR; ActRIIA binders that block signalling in cells without affinity maturation; nanomolar Nipah virus binders by de novo design and scaffold re-engineering; kinase binders across the peptide-to-protein size range; and de novo binders to a free carbohydrate, including a design that discriminates between blood-group antigens.

Open questions remain. Codesign outperforms sequence redesign on average, but the margin varies across targets, and which interface properties benefit most from joint optimisation is not yet clear. The carbohydrate results motivate extension to other non-protein modalities. More broadly, latent generative search — our framework pairing a learned generative prior with adaptive inference-time search — translates a principle established in other areas of artificial intelligence into experimentally validated protein design across target classes that have been difficult to address. With an expanded repertoire of scoring functions and optimisation objectives, latent generative search could unlock further new classes of protein design problems. Code and model weights are released to support reproduction and extension (Methods Section 10).

## 10 Methods

### Latent generative search and base model

The base generative model, Proteína-Complexa, and the inference-time search algorithms used here were introduced previously [18]; we summarise them for completeness and refer to that work and to La-Proteína [19] for full specifications. The model builds on La-Proteína’s partially latent representation, in which backbone C_*α*_ coordinates are modelled explicitly and each residue’s amino-acid identity and remaining atoms are encoded into a continuous latent variable by a variational autoencoder; a rectified-flow flow-matching model generates backbone coordinates and latents jointly [20]. The denoiser is a transformer with pair-biased attention over a concatenated sequence of noisy binder tokens (C_*α*_ coordinates and latents) and clean target tokens (Atom37 coordinates, amino-acid identity and binary hotspot tokens); target conditioning is applied to the flow model only, leaving the autoencoder target-agnostic. During training the binder C_*α*_ coordinates are perturbed with a global translation (*c*_*d*_ = 0.2 nm) so that the model reasons over binder placement. We use separate models for protein-protein interactions and for binder design to ligand targets. Extended model details are presented in Supplementary S1; full architecture and training hyperparameters are given in ref. [18] (its Appendix G, Table 14) and are reproduced in Supplementary Table S4.

### Training data

Four datasets are used [18]: Foldseek AFDB monomer cluster representatives [47]; Teddymer dimer cluster representatives (filtered for interface pLDDT > 70, interface predicted aligned error < 10 and interface length > 10); protein multimers filtered from the PDB [23]; and the filtered PLINDER protein–ligand dataset [48]. Teddymer is derived from AFDB50 structures with TED domain annotations [22, 49] (47M structures), split into domains, filtered for spatial proximity and complete CATH annotation [50] to give 10M dimers, then clustered to 3.5M representatives [21]. The variational autoencoder is pretrained on AFDB monomers (500k steps, 16 GPUs) and fine-tuned on 110,976 PDB chains (length 50–256, resolution better than 5.0 Å; 40k steps). The flow model is pretrained on AFDB monomers and then trained on Teddymer and PDB multimers for protein targets; small-molecule targets use PLINDER and AFDB monomers with low-rank adaptation [51].

### Inference-time search

We use best-of-*N* sampling, beam search, Feynman–Kac steering and Monte Carlo tree search adapted to flow models [52–55]. Candidate states are rolled out to clean structures, decoded, folded and scored, with the search applied every *K* denoising steps. Default hyperparameters are beam width *N* = 4, branching factor *L* = 4 and step length *K* = 100; Feynman–Kac steering additionally uses inverse temperature *β* = 10 [18]. Scaffold re-engineering (Nipah) uses a diffuse–denoise procedure that partially noises an input binder and codesigns a related backbone and sequence.

### Reward functions and success criteria

Search and filtering use combinations of complementary rewards (Supplementary Table S5). In-silico success for protein targets follows AlphaFold2-Multimer interface metrics (pLDDT > 90, interface predicted aligned error < 7.0, binder RMSD < 1.5 Å) via ColabDesign [26, 56]; small-molecule and carbohydrate targets use RosettaFold3 (minimum interface predicted aligned error < 2, binder RMSD < 2 Å, ligand RMSD < 5 Å) [27]. Hydrogen-bond energies are computed with the Rosetta energy function via Tmol and hydrogen bonds detected with HBPlus [24, 25, 57]. Per-campaign reward mixtures were as follows. PDGFR/PD-L1, the massive-scale benchmark and PAK1 used beam search with an AlphaFold2 interface-pAE reward. Nipah de novo design used beam search with a Boltz-2 maximum interface score combined with shape complementarity, interface hydrophobicity and hydrogen-bond satisfaction [34]. ActRIIA used averaged complex interface scores for ranking across beam search, Feynman–Kac steering and tree search. Carbohydrate design used pocket-burial and physics-based Tmol hydrogen-bond rewards together with RosettaFold3 interface confidence. After every search step, the RosettaFold3-predicted bound sugar conformation replaced the preceding ligand pose before the next generative step, allowing the search to account for binding-dependent glycan flexibility.

### Designed monomers

Designs were filtered by AlphaFold2 single-sequence prediction (mean pLDDT > 90, RMSD < 2 Å to the design model). Genes were codon-optimised, cloned by Golden Gate assembly and expressed in *E. coli* BL21(DE3) by auto-induction. Proteins were purified by Ni-NTA affinity and size-exclusion chromatography (SEC); yields were determined from A_280_. Oligomeric state was assessed by SEC coupled to multi-angle light scattering, and secondary structure and thermostability by circular dichroism (25–95 °C). A representative design was crystallised and its structure solved by X-ray crystallography.

### PDGFR and PD-L1

Interface hotspots were identified by Rosetta alanine scanning of known complexes (retaining positions with favourable binding contribution; top nine per target). For each hotspot combination, 1,500 candidates (55–95 aa) were generated by beam search with an AlphaFold2 interface-pAE reward. Stage-one filtering used isoelectric point, ESM pseudo-likelihood, minimum interface-pAE, complex interface-pTM and pLDDT, and cysteine count, followed by TM-score clustering at 0.8; stage two validated monomeric structures with OpenFold3 and Boltz-2 (pLDDT > 80, RMSD < 2 Å). 192 designs per target were expressed in *E. coli*, purified, and assayed by surface plasmon resonance with kinetic or steady-state fitting of *K*_D_.

### Nipah virus

De novo designs were generated conditioned on hotspots at the NiV-G receptor-binding site using beam search with the multi-objective reward above; scaffold re-engineering used the diffuse–denoise procedure. Designs were expressed in *E. coli* and purified by Ni-affinity and size-exclusion chromatography, and binding was measured by surface plasmon resonance against NiV-G with *K*_D_ from kinetic or steady-state fitting.

### Massive-scale benchmark

Targets were sourced commercially (Supplementary Table S6). For each target, design tasks spanned multiple hotspot combinations (about ten where literature hotspots were unavailable), with an overall screening budget of 10,000 sequences per target. Per task, a single Proteína-Complexa run with standard beam search and an AlphaFold2 interface-pAE reward produced about 27,000 candidates, filtered to the top 1,000 by predicted aligned error; in-silico success required minimum interface-pAE < 2, complex pLDDT > 0.8, interface-pTM > 0.7 and fewer than five C_*α*_ clashes. For the screening of diverse Proteína-Complexa-generated sequences across all targets and hotspots, diversity selection clustered candidates by predicted structure and cycled ranking criteria across clusters and hotspots (Supplementary Fig. S6).

For the separate baseline comparison experiment using the top-ranked in silico hotspots only both Proteína-Complexa with latent generative search and the baseline methods (BindCraft, RFdiffusion, RFdiffusion3, BoltzGen with varying sequence design approaches) were run under a matched budget of about 32 GPU hours per target with minimum interface score as the primary metric and eight cycles of soluble ProteinMPNN redesign [13, 14, 29, 30, 36]. For each method, sequence design setting and target, 400 sequences were tested. Here, the final candidates were selected greedily based on minimum ipSAE without clustering (details in Supplementary S4) [58]. The same protocol was used in the sampling temperature ablation experiments. The overall campaign used more than 140,000 GPU hours in total for candidate generation and filtering.

Designs were synthesised as oligo pools, cloned into a phagemid and screened by multiplexed phage display [35]; sequencing reads were merged [59] and counted by exact match. Hits were called by two methods whose union was used: a quantile-ratio test (hit if the minimum across replicates exceeds the upper-tail quantile, *q* = 20) and an enrichment *z*-test on output/input ratios with a robust null (median and median absolute deviation) and Benjamini–Hochberg control of the false-discovery rate (Supplementary S4.2).

### ActRIIA

Hotspots were taken from ActRIIA complexes with bimagrumab and Activin A. Across seven design rounds varying sampling size, search algorithm and hotspot conditioning, candidates were ranked by averaged complex interface score and clustered by scaffold (Foldseek) to select 200 designs. Proteins were expressed in *E. coli* BL21(DE3), purified by Ni-affinity with TEV cleavage and a second Ni step, and assayed by surface plasmon resonance (Biacore) against immobilised ActRIIA over a concentration series in two independent runs. Cellular activity was measured in a Smad2/3-responsive luciferase reporter in HEK293 cells stimulated with Activin A (20 ngmL^−1^) or GDF8/myostatin (200 ngmL^−1^), with three-fold serial dilutions of each design.

### Kinase binders

PAK1 mini-proteins (49–74 aa) were generated by beam search with an AlphaFold2 interface-pAE reward and screened by a split-neomycin-resistance complementation assay in *E. coli* [42, 43]; enrichment was computed from next-generation sequencing as log_2_ fold change and classified by a two-component Gaussian mixture model. Selected candidates were validated by HA-Trap co-immunoprecipitation in HEK293T cells with quantitative Western blotting (one-sample *t*-test versus 1). CK1*δ* peptides (<31 aa) were synthesised by Fmoc solid-phase synthesis with a biotinylated lysine, captured on streptavidin beads, and assayed for binding to purified GST–CK1*δ* by Western blot (one-tailed *t*-test versus scrambled control). *K*_D_ values for CK1 were determined with microscale thermophoresis on a Monolith X instrument (NanoTemper Technologies) using the 670 nm detection channel.

### Carbohydrate binders

Proteína-Complexa was conditioned on the type II A pentasaccharide. Pocket burial and physics-based Tmol hydrogen-bond energies rewarded deep pockets and precise polar contacts during search. RosettaFold3 was run without ligand templating after every search step; its predicted bound sugar conformation was fed back as the ligand pose for the next step so that binder generation and glycan conformation co-evolved. Twenty-three designs (all <10 kDa) were cloned into a T7 vector, expressed in *E. coli* BL21(DE3) and purified by Ni-NTA. Purified proteins were screened against a fluorescently labelled (iFluor488) type II A pentasaccharide by fluorescence polarization (FP) in duplicate; designs exceeding +10 mP over a tracer-only control were counted as hits, and dissociation constants were obtained from FP titration series normalised to the tracer-only control. FP specificity was tested by competition with excess unlabelled type II A pentasaccharide and, for design 15, with 30 mM D-galactose and 250 *μ*M L-fucose. Orthogonal binding was measured by biolayer interferometry, both with protein as analyte over immobilised biotinylated glycan (referenced against an analyte-only control) and with immobilised binder against a glycan–DNA conjugate (pentasaccharide coupled to an amine-modified oligonucleotide by reductive amination; a trivalent analyte poorly described by a 1:1 model). Agglutination was assessed by immobilising His_6_-tagged binders on Ni-NTA agarose beads to confer multivalency, incubating with type A_2_ or type B red blood cells (commercial source) in a plate format, and reading OD_600_ in triplicate against blank-bead controls, with significance from a Z-score. Thermostability and ligand-induced stabilisation were measured by circular dichroism at 222 nm with and without the type B pentasaccharide. Structural and sequence novelty of design 3 was assessed by search against the non-redundant sequence database and by Foldseek structural search [47].

## Supplementary Material

Supplement 1

## Data Availability

Inference code for latent generative search and model weights for Proteína-Complexa are released [18], see https://research.nvidia.com/labs/genair/proteina-complexa/ and https://github.com/NVIDIA-Digital-Bio/proteina-complexa.
