# Supplementary material for: Latent generative search unlocks de novo design of untapped biomolecular interactions at scale
Source: bioRxiv. 2026 Sep 18:2026.09.12.751118. Preprint. [Version 1] doi: 10.64898/2026.09.12.751118 (PMC13596280; doi:10.64898/2026.09.12.751118)
Supplement: Supplementary file 1 [file NIHPP2026.09.12.751118v1-supplement-1.pdf]

bioRxiv preprint doi: https://doi.org/10.64898/2026.09.12.751118; this version posted September 18, 2026. The copyright holder for this preprint (which was not certified by peer review) is the author/funder, who has granted bioRxiv a license to display the preprint in perpetuity. It is made available under a CC-BY 4.0 International license.

Supplementary contents

S1 Model architecture and latent generative search (p. 32).
S2 Biophysical characterization of designed monomers (p. 37).
S3 PDGFR and PD-L1 filtering and hotspot analysis (p. 39).
S4 Massive-scale benchmark: extended analysis and hit calling (p. 40).
S5 ActRIIA experimental methods (p. 46).
S6 PAK1 and CK1δ experimental methods (p. 48).
S7 Carbohydrate-binder experimental methods (p. 51).
S8 Supplementary figures (p. 54).
S9 Supplementary tables (p. 70).

bioRxiv preprint doi: https://doi.org/10.64898/2026.09.12.751118; this version posted September 18, 2026. The copyright holder for this preprint (which was not certified by peer review) is the author/funder, who has granted bioRxiv a license to display the preprint in perpetuity. It is made available under a CC-BY 4.0 International license.

S1 Model architecture and latent generative search

This section gives a self-contained mathematical summary of the model used in this study. It follows the notation of Proteína-Complexa throughout and condenses the full treatments in La-Proteína and Proteína-Complexa [18, 19]. The purpose is to make precise how a complete binder sequence and all-atom structure are generated, how a fixed target conditions that distribution, and how external rewards alter sampling without retraining the base model.

S1.1 Protein representation and notation

Let a binder contain N residues. We write $\mathbf{x}^{C_\alpha} \in \mathbb{R}^{N\times 3}$ for its C_α coordinates, $\mathbf{x}^{\neg C_\alpha} \in \mathbb{R}^{N\times 36\times 3}$ for the remaining Atom37 coordinates, and $\mathbf{s} \in \{0,\ldots,19\}^N$ for its amino-acid sequence. Atom37 is a fixed-size storage representation. The residue-dependent mask $\mathbf{m}(\mathbf{s}) \in \{0,1\}^{N\times 36}$ indicates which non-C_α atom slots are present for each residue type. The per-residue latent representation is $\mathbf{z} \in \mathbb{R}^{N\times d_z}$, with $d_z = 8$. A complete binder is abbreviated by

$$\mathbf{x} = (\mathbf{x}^{C_\alpha}, \mathbf{x}^{\neg C_\alpha}, \mathbf{s}). \tag{1}$$

The fixed target context is denoted by $\mathbf{c}^{\text{target}}$. For protein targets it contains Atom37 coordinates, residue identities, chain and residue indices, and optional binary interface-hotspot indicators. For ligand targets it contains atom types and names, coordinates, charges, graph-Laplacian positional encodings, bond masks, and bond orders.

The partially latent construction preserves the spatial backbone explicitly while placing residue identity and all non-C_α geometry in a fixed-dimensional continuous latent space. The joint conditional density of a binder and its latent representation factorizes as

$$\begin{aligned} &p_{\phi,\theta}\left(\mathbf{x}^{C_\alpha}, \mathbf{x}^{\neg C_\alpha}, \mathbf{s}, \mathbf{z} \mid \mathbf{c}^{\text{target}}\right) \\ &\quad = p_\theta\left(\mathbf{x}^{C_\alpha}, \mathbf{z} \mid \mathbf{c}^{\text{target}}\right) p_\phi\left(\mathbf{x}^{\neg C_\alpha}, \mathbf{s} \mid \mathbf{x}^{C_\alpha}, \mathbf{z}\right). \end{aligned} \tag{2}$$

The corresponding conditional density over complete binders is obtained by marginalizing the latent variable:

$$p_{\phi,\theta}(\mathbf{x} \mid \mathbf{c}^{\text{target}}) = \int p_\theta(\mathbf{x}^{C_\alpha}, \mathbf{z} \mid \mathbf{c}^{\text{target}}) p_\phi(\mathbf{x}^{\neg C_\alpha}, \mathbf{s} \mid \mathbf{x}^{C_\alpha}, \mathbf{z})\, \mathrm{d}\mathbf{z}. \tag{3}$$

The first factor is generated by a continuous flow, and the second factor is the autoencoder decoder. Thus the main generator does not have to evolve a mixed categorical–continuous state or a residue-dependent number of atoms.

where coordinate-valued quantities are represented in the model's internal length units.

bioRxiv preprint doi: https://doi.org/10.64898/2026.09.12.751118; this version posted September 18, 2026. The copyright holder for this preprint (which was not certified by peer review) is the author/funder, who has granted bioRxiv a license to display the preprint in perpetuity. It is made available under a CC-BY 4.0 International license.

Complex generation additionally uses a global translation

$$\mathbf{d} \sim \mathcal{N}(\mathbf{0}, c_d^2 \mathbf{I}_3), \qquad c_d = 0.2\,\text{nm}, \tag{4}$$

which is independent of the reference variables and is broadcast over all binder residues as $\mathbf{1}_N \mathbf{d}^\mathsf{T}$. Separate interpolation times $t_x, t_z \in [0, 1]$ are used for the backbone and latent channels:

$$\mathbf{x}_{t_x}^{C_\alpha} = (1 - t_x) \left(\mathbf{x}_0^{C_\alpha} + \mathbf{1}_N \mathbf{d}^\mathsf{T} \right) + t_x \mathbf{x}_1^{C_\alpha}, \tag{5}$$

$$\mathbf{z}_{t_z} = (1 - t_z)\mathbf{z}_0 + t_z \mathbf{z}_1. \tag{6}$$

Translation noise is specific to complex generation: it makes absolute binder placement relative to the target a learned part of denoising rather than a nearly fixed low-frequency feature.

The conditional velocity network has two outputs,

$$\mathbf{v}_x^\theta \in \mathbb{R}^{N \times 3}, \qquad \mathbf{v}_z^\theta \in \mathbb{R}^{N \times d_z}, \tag{7}$$

and receives the interpolated binder state, target context, and both times. Because the interpolants are linear, their target velocities are

$$\mathbf{u}_x = \mathbf{x}_1^{C_\alpha} - \mathbf{x}_0^{C_\alpha} - \mathbf{1}_N \mathbf{d}^\mathsf{T}, \tag{8}$$

$$\mathbf{u}_z = \mathbf{z}_1 - \mathbf{z}_0. \tag{9}$$

The conditional flow-matching objective is

$$\begin{aligned} \mathcal{L}_{\text{CFM}}(\theta) = \mathbb{E}\Big[&\Big\| \mathbf{v}_x^\theta \Big(\mathbf{x}_{t_x}^{C_\alpha}, \mathbf{z}_{t_z}, \mathbf{c}^{\text{target}}, t_x, t_z \Big) - \mathbf{u}_x \Big\|_\text{F}^2 \\ &+ \Big\| \mathbf{v}_z^\theta \Big(\mathbf{x}_{t_x}^{C_\alpha}, \mathbf{z}_{t_z}, \mathbf{c}^{\text{target}}, t_x, t_z \Big) - \mathbf{u}_z \Big\|_\text{F}^2 \Big]. \end{aligned} \tag{10}$$

The expectation is over binder–target pairs $(\mathbf{x}, \mathbf{c}^{\text{target}}) \sim p_{\text{data}}$, encoder samples $\mathbf{z}_1 \sim q_\psi(\mathbf{z} \mid \mathbf{x})$, independent reference noise $\mathbf{x}_0^{C_\alpha}$ and $\mathbf{z}_0$, translation noise $\mathbf{d}$, and independently sampled interpolation times.

Following La-Proteína, define the time densities

$$p_x(t) = 0.02\ \text{Unif}(t; 0, 1) + 0.98\ \text{Beta}(t; 1.9, 1), \tag{11}$$

$$p_z(t) = 0.02\ \text{Unif}(t; 0, 1) + 0.98\ \text{Beta}(t; 1, 1.5), \tag{12}$$

and draw

$$t_x \sim p_x, \qquad t_z \sim p_z, \qquad t_x \perp t_z. \tag{13}$$

The unequal schedules emphasize different noise regimes for global backbone geometry and local atomistic detail.

bioRxiv preprint doi: https://doi.org/10.64898/2026.09.12.751118; this version posted September 18, 2026. The copyright holder for this preprint (which was not certified by peer review) is the author/funder, who has granted bioRxiv a license to display the preprint in perpetuity. It is made available under a CC-BY 4.0 International license.

S1.2 Target conditioning and neural architecture

The denoiser operates on a single concatenated sequence containing noisy binder tokens and clean target tokens. Binder token i contains its interpolated C_α coordinate $\mathbf{x}^{C_\alpha}_{t_x,i}$, its interpolated latent vector $\mathbf{z}_{t_z,i}$, and a positional encoding of its sequence index. Protein-target tokens contain amino-acid identity, fixed Atom37 geometry, chain identity, and optional hotspot indicators. Ligand-target tokens instead represent individual target atoms using the element, charge, aromaticity, and other atom-level features defined above. All token types are projected into a common hidden dimension before being processed jointly.

Supplementary Fig. S10a shows the construction of the initial sequence representation. Sinusoidal encodings of sequence indices are linearly projected and added to learned projections of the coordinate and feature channels. The coordinate projections allow the network to distinguish the current noisy binder geometry from the fixed target geometry, while the feature projections distinguish binder latent states, protein-target residues, and ligand-target atoms. A small set of learned register tokens is appended to the physical tokens. These registers provide sequence-level workspace without being associated with a residue, atom, or generated coordinate.

The two interpolation times enter through a separate conditioning pathway (Supplementary Fig. S10b). Sinusoidal embeddings of t_x and t_z are concatenated and transformed by an MLP to obtain a conditioning vector $\mathbf{c}_t$. This vector is aligned with the binder and target tokens and is used throughout the transformer to modulate normalization and residual updates. The conditioning entries associated with register tokens are set to zero. Keeping t_x and t_z separate allows the network to adapt its computation independently to the noise levels of the backbone and latent channels.

The initial pair representation is constructed as shown in Supplementary Fig. S10c. For protein pairs, its inputs include coordinate-derived pair distances, relative sequence separation, chain identity, and the distinction between binder and target tokens. Ligand pairs additionally contain bond connectivity and bond order, and protein–ligand pairs encode their current cross-interface geometry. The geometric and sequence-derived features are concatenated and adaptively normalized using the time representation. A transformed embedding of t_x and t_z is then concatenated with these features. Rows and columns introduced by the register tokens are zero-padded so that registers participate in sequence attention without introducing artificial geometric relationships.

Each transformer block maintains a sequence representation $\mathbf{H} \in \mathbb{R}^{L\times d_s}$ and a pair representation $\mathbf{P} \in \mathbb{R}^{L\times L\times d_p}$, where L includes the binder, target, and register tokens. As illustrated in Supplementary Fig. S11a, adaptively normalized sequence states are projected into queries, keys, and values. A normalized linear projection of the pair representation supplies an additive bias to each attention head:

$$\mathbf{A}_h = \mathrm{softmax}\left(\frac{\mathbf{Q}_h\mathbf{K}_h^\intercal}{\sqrt{d_h}} + \mathbf{B}_h(\mathbf{P})\right). \tag{14}$$

Consequently, attention between two tokens depends both on their sequence representations and on their pairwise geometric or chemical relationship. The attention outputs

bioRxiv preprint doi: https://doi.org/10.64898/2026.09.12.751118; this version posted September 18, 2026. The copyright holder for this preprint (which was not certified by peer review) is the author/funder, who has granted bioRxiv a license to display the preprint in perpetuity. It is made available under a CC-BY 4.0 International license.

are concatenated, linearly projected, adaptively scaled, and added to the sequence residual stream. A second adaptively normalized sublayer applies a linear–SwiGLU transition followed by another conditioned residual update.

The pair representation is updated after the sequence attention operation (Supplementary Fig. S11b). Two learned projections of the normalized sequence states are combined elementwise to produce a sequence-derived pair update. This update is added to $\mathbf{P}$, after which outgoing and incoming multiplicative triangle layers propagate information over token triplets. A normalized feed-forward pair transition, implemented as linear–ReLU–linear transformations, completes the pair update. Residual connections around these operations preserve the previous pair state while allowing geometric and interaction information to accumulate over successive blocks.

The adaptive operations used in the sequence pathway are detailed in Supplementary Fig. S11c–d. Suppressing token and channel indices, their action can be summarized as

$$\mathrm{AdaNorm}(\mathbf{h}, \mathbf{c}_t) = \sigma(g(\mathrm{RN}(\mathbf{c}_t))) \odot \mathrm{RN}(\mathbf{h}) + b(\mathrm{RN}(\mathbf{c}_t)), \tag{15}$$

$$\mathrm{AdaScale}(\mathbf{u}, \mathbf{c}_t) = \sigma(s(\mathrm{RN}(\mathbf{c}_t))) \odot \mathbf{u}, \tag{16}$$

where g, b, and s are learned projections and RN denotes the base normalization operation. The first operator conditions both the scale and shift of a normalized activation, whereas the second controls the magnitude of a residual branch. This makes the computation in every block explicitly dependent on both flow times.

After the final transformer block, only the binder-token representations are passed to two output projections. The coordinate head produces $\mathbf{v}_x^\theta$, with one three-dimensional velocity per binder residue, and the latent head produces $\mathbf{v}_z^\theta$, with one latent-space velocity per binder residue. Target and register outputs are discarded. Target coordinates remain fixed during integration and influence generation only through the joint sequence representation, pair features, and attention biases.

Only the conditional flow model receives $\mathbf{c}^{\mathrm{target}}$. The atomistic autoencoder remains a target-independent monomer encoder and decoder, allowing its pretrained sequence–structure representation to be reused unchanged for conditional generation.

Training proceeds from broad protein priors to interaction-specific data. The autoencoder is pretrained on AlphaFold Database monomers and fine-tuned on PDB chains. The conditional flow combines AFDB monomers, Teddymer synthetic domain–domain interfaces, and filtered experimental PDB multimers [21–23]. For ligand-conditioned models, filtered PLINDER complexes are added and the protein model is adapted with low-rank updates [48, 51]. Target coordinates are held fixed throughout generation.

S1.3 Sampling dynamics and decoding

At inference, Gaussian binder states are advanced from $(t_x, t_z) = (0, 0)$ to $(1, 1)$. For the Gaussian probability paths used here, the learned velocity determines the scores $\boldsymbol{\zeta}_x$ and $\boldsymbol{\zeta}_z$, the gradients of the log intermediate densities. The stochastic sampler may

bioRxiv preprint doi: https://doi.org/10.64898/2026.09.12.751118; this version posted September 18, 2026. The copyright holder for this preprint (which was not certified by peer review) is the author/funder, who has granted bioRxiv a license to display the preprint in perpetuity. It is made available under a CC-BY 4.0 International license.

be written

$$d\mathbf{x}_{t_x}^{C_\alpha} = \mathbf{v}_x^\theta \, dt_x + \beta_x(t_x)\boldsymbol{\zeta}_x \, dt_x + \sqrt{2\beta_x(t_x)\eta_x} \, d\mathbf{W}_{t_x}, \tag{17}$$

$$d\mathbf{z}_{t_z} = \mathbf{v}_z^\theta \, dt_z + \beta_z(t_z)\boldsymbol{\zeta}_z \, dt_z + \sqrt{2\beta_z(t_z)\eta_z} \, d\mathbf{W}_{t_z}, \tag{18}$$

where $\beta_x(t_x) = 1/t_x$ and $\beta_z(t_z) = \frac{\pi}{2}\tan[\frac{\pi}{2}(1 - t_z)]$. Noise scales $\eta_x, \eta_z < 1$, typically 0.1, implement low-temperature sampling. Once the terminal state is reached, the decoder maps $(\mathbf{x}_1^{C_\alpha}, \mathbf{z}_1)$ to a categorical sequence and non-C_α Atom37 coordinates. Consequently each trajectory directly returns a sequence–structure pair; no inverse-folding model is required.

S1.4 Reward-guided inference-time search

Let $p^\theta(\mathbf{x} \mid \mathbf{c}^{\text{target}})$ denote the terminal distribution of the conditional generator, $R(\mathbf{x})$ a real-valued reward, and $S(\mathbf{x})$ a Boolean success criterion. Rewards used here include structure-prediction interface confidence, hydrogen-bond energy and satisfaction, shape complementarity, pocket burial and physicochemical penalties. A reward for an intermediate state is approximated by stochastically rolling that state to $t_x = t_z = 1$, decoding it, and evaluating the resulting clean complex. This keeps the scoring model outside the generator and permits non-differentiable rewards.

Best-of-N.

Independent terminal samples are ranked by R. This increases inference compute without changing any trajectory and provides the reference case for adaptive methods.

Beam search.

Let $\mathcal{B}_k$ be a beam of N partially denoised states at search stage k. Each state is advanced for K discretized steps along L independent stochastic branches, producing NL candidates $\mathcal{C}_{k+1}$. With rollout reward $\widehat{R}$, the next beam is

$$\mathcal{B}_{k+1} = \mathrm{TopN}_{c \in \mathcal{C}_{k+1}} \, \widehat{R}(c). \tag{19}$$

Completed rollout samples satisfying S are retained rather than discarded. Unless stated otherwise, $N = 4$, $L = 4$, $K = 100$, and the base trajectory contains 400 discretized denoising steps.

Feynman–Kac steering.

Feynman–Kac steering targets the exponentially tilted terminal density

$$\widetilde{p}(\mathbf{x} \mid \mathbf{c}^{\text{target}}) \propto p^\theta(\mathbf{x} \mid \mathbf{c}^{\text{target}}) \exp\{\beta_{\text{FK}} R(\mathbf{x})\}\,. \tag{20}$$

bioRxiv preprint doi: https://doi.org/10.64898/2026.09.12.751118; this version posted September 18, 2026. The copyright holder for this preprint (which was not certified by peer review) is the author/funder, who has granted bioRxiv a license to display the preprint in perpetuity. It is made available under a CC-BY 4.0 International license.

It uses the same branch-and-rollout construction as beam search, but resamples N particles from $\mathcal{C}_{k+1}$ with probabilities

$$\Pr(c) = \frac{\exp\{\beta_{\mathrm{FK}} \widehat{R}(c)\}}{\sum_{c' \in \mathcal{C}_{k+1}} \exp\{\beta_{\mathrm{FK}} \widehat{R}(c')\}}. \tag{21}$$

We use $\beta_{\mathrm{FK}} = 10$. Deterministic top-N selection concentrates more aggressively, whereas resampling preserves reward-weighted diversity.

Monte Carlo tree search.
MCTS interprets discretized denoising as a tree whose nodes are partially latent states. Selection, stochastic expansion, terminal rollout and reward backpropagation are repeated. For a child j of node i, the exploration–exploitation score has the form

$$\mathrm{UCB}(j) = \frac{Q_j}{V_j} + C\sqrt{\frac{\log V_i}{V_j}}, \tag{22}$$

where Q_j is accumulated rollout reward, V_j is the child visit count, V_i is the parent visit count and C is the exploration constant. Unlike beam search, MCTS can revisit an earlier branching decision when later rollouts reveal that it is promising.

S1.5 Codesign-based scaffold re-engineering

For scaffold re-engineering, an observed binder is encoded, then moved to an intermediate noise level (τ_x, τ_z) using Eqs. (5) and (6). Denoising from that state regenerates both backbone and latent variables conditional on the same target. The pair (τ_x, τ_z) controls the edit radius: small values retain little of the input, whereas values near one produce local sequence and geometry changes. Because the decoder returns both modalities, this procedure is codesign rather than fixed-backbone sequence redesign. The Nipah campaign applies this diffuse–denoise construction alongside fully de novo sampling.

S2 Biophysical characterization of designed monomers: experimental methods

Design filtering. Structure predictions for La-Proteína co-designed and ProteinMPNN-designed sequences for the unconditional design benchmark set were generated in single-sequence mode with 3 recycles using the *alphafold2_ptm_model_1* of AF2 in its ColabFold implementation. Designs were considered successful if the predicted structures had a mean pLDDT > 90 and an RMSD < 2 Å from the design model. All successful designs were subsequently manually screened; a subset of 49 designs were selected for experimental analysis. La-Proteína co-generated sequences were preferred whenever metrics matched those of the respective ProteinMPNN sequence. Sequence identity and structural similarity were further analysed using HHblits and the Foldseek web server, respectively.

bioRxiv preprint doi: https://doi.org/10.64898/2026.09.12.751118; this version posted September 18, 2026. The copyright holder for this preprint (which was not certified by peer review) is the author/funder, who has granted bioRxiv a license to display the preprint in perpetuity. It is made available under a CC-BY 4.0 International license.

For the high-temperature generation set, designs were predicted both in single-sequence mode and with MSA. Designs were selected for further analysis if they were confidently predicted with MSA (mean pLDDT $>$ 80 and RMSD $<$ 2 Å from the design model), while being poorly predicted in single-sequence mode (mean pLDDT $<$ 80 and RMSD $>$ 4 Å). Successful designs were manually screened and filtered based on sequence identity (not higher than 40%), yielding a final set of 13 designs.

Cloning, expression, purification, and yield quantification. Genes coding for the designed proteins were codon-optimised for *Escherichia coli* K-12 and synthesised by GenScript. Gene fragments were cloned into the LM627 bacterial expression vector using BsaI-mediated Golden Gate assembly, replacing the vector's *ccdB* selection marker. The assembly products were transformed into *E. coli* NEB5α cells (New England Biolabs) through heat-shock transformation and selected on LB agar (Carl Roth) supplemented with kanamycin (VWR). Plasmids were purified from overnight cultures using the QIAprep Spin Miniprep Kit (Qiagen) and verified through Sanger sequencing (Eurofins Genomics). The sequence-verified plasmids were subsequently transformed into *E. coli* BL21(DE3) cells (New England Biolabs) for protein expression.

Proteins were expressed by auto-induction in TBII medium (MP Biomedicals), supplemented with kanamycin (VWR), a 50×5052 autoinduction mixture, 20 mM $MgSO_4$, and a trace metal mix. The cultures were initially grown at 37 °C for 8 hours, followed by incubation at 18 °C for 24 hours with shaking. Cells were harvested by centrifugation at 5,000 g, resuspended in lysis buffer (50 mM Tris, pH 8.0, 250 mM NaCl, 20 mM imidazole) containing lysozyme and DNase I (Sigma-Aldrich), and lysed by sonication. Clarified lysates obtained by centrifugation were purified using immobilised metal affinity chromatography with HisPur Ni-NTA resin (Thermo Fisher Scientific). Samples were washed with wash buffer (50 mM Tris, pH 8.0, 250 mM NaCl, 20 mM imidazole) and eluted using the same buffer containing 500 mM imidazole. Eluted proteins underwent further purification by size-exclusion chromatography (SEC) on a Superdex 75 Increase 10/300 GL column (Cytiva), using an ÄKTA Pure system (Cytiva) equilibrated in 25 mM Tris, pH 8.0, and 150 mM NaCl.

Protein yields were estimated from the SEC chromatograms recorded at 280 nm by integrating the absorbance peak corresponding to the expected elution of the target species. Peak areas were converted to protein mass using sequence-derived extinction coefficients, corrected for the ÄKTA flow-cell path length, and scaled from the injected sample to the total Ni-NTA eluate. To compare soluble expression yields between different sequence design strategies, a subset of constructs sharing the same backbone but differing in sequence (co-generated by La-Proteína or fixed-backbone design with ProteinMPNN) was expressed and purified in three independent replicates.

Size-exclusion chromatography coupled to multi-angle light scattering (SEC–MALS). Where needed, SEC-purified proteins were concentrated prior to further analysis to ensure sufficient scattering signal. Molecular weight was determined by SEC–MALS using an ÄKTA Pure system equipped with a Superdex™ 75 10/300 GL column and a Wyatt miniDAWN™ detector. Extinction coefficients were calculated from the protein sequence and used by the ASTRA software to determine molecular weights.

bioRxiv preprint doi: https://doi.org/10.64898/2026.09.12.751118; this version posted September 18, 2026. The copyright holder for this preprint (which was not certified by peer review) is the author/funder, who has granted bioRxiv a license to display the preprint in perpetuity. It is made available under a CC-BY 4.0 International license.

Circular dichroism (CD). Proteins were diluted and/or buffer-exchanged into PBS (100 mM KCl, 10 mM phosphate) buffer to concentrations between 0.1–0.3 mg/mL prior to analysis and measured in a 2 mm Hellma QS cuvette. A JASCO J-1500 spectrometer equipped with a Xe lamp was used in combination with the Spectra Manager^{TM} CFR software. Spectra between 200 and 250 nm were initially obtained for each sample at 25 °C to confirm protein folding. Subsequently, temperature-ramp measurements at three distinct wavelengths (208, 222, and 230 nm) were recorded in steps of 10 °C between 25 °C and 95 °C. Once overall stability was confirmed, full spectra in the range of 200–250 nm were obtained at the start and end points (25 °C, 95 °C, and return to 25 °C). All data were baseline-corrected with PBS.

Crystallization, data collection, and structure determination. Protein crystals of LP46 (3.9 mg/mL) were obtained by hanging drop vapor diffusion at room temperature, using 1 μL protein in 20 mM TRIS (pH 8.0), 150 mM NaCl buffer, and 1 μL reservoir solution. The protein sample crystallized in 0.1 M NaAcet (pH 4.3), 32.5 % (v/v) ethylene glycol within 3 days. To improve crystal growth, microseeding was applied using protein crystals of the same condition in the subsequent finescreen. The crystal samples were flash-frozen in liquid nitrogen.

The collection of crystallographic data was conducted at the BESSY II Beamline MX14.1 [60]. Diffraction data were processed using xds, and anisotropy corrected and merged using STARANISO [61, 62]. Phases were determined by Phaser molecular replacement, using the designed structure of LP46 as search model [63]. Manual model refinement was performed in Coot (0.9.8) and automated refinement was carried out with PHENIX.refine (v2.1) [64, 65]. Data collection, phasing, and refinement statistics are summarized in Table S7. Data processing software packages were used from the SBGrid software environment, which provides curated, version-controlled structural biology applications and reproducible execution environments across platforms [66].

The crystal structure is shown in Fig. 2f and a detailed per-residue plot of the root mean square deviation between the experimentally determined structure and the computational design generated by La-Proteína is presented in Supplementary Fig. S5.

Data processing. All data were processed and plotted using Python.

S3 PDGFR and PD-L1 binder design: filtering and hotspot analysis

S3.1 Design filtering pipeline

As described in the protein-binder section of the main text (Section 4), a two-stage filtering pipeline was applied to Proteína-Complexa-generated candidates for PDGFR and PD-L1. In the initial screening stage, designs were required to meet the following quantitative thresholds:

Isoelectric Point $\leq$ 6.0, selected to ensure favorable solubility and charge profiles under physiological conditions.
ESM Pseudolikelihood $\leq$ 10.0, utilized as a metric for sequence fitness and evolutionary plausibility.

bioRxiv preprint doi: https://doi.org/10.64898/2026.09.12.751118; this version posted September 18, 2026. The copyright holder for this preprint (which was not certified by peer review) is the author/funder, who has granted bioRxiv a license to display the preprint in perpetuity. It is made available under a CC-BY 4.0 International license.

Minimum IPAE ≤ 1.9, ensuring high-confidence relative positioning and orientation of the binder at the target interface.
Complex iPTM ≥ 0.80, prioritizing designs with a high probability of correct interface assembly.
Complex pLDDT ≥ 0.88, ensuring high local structural confidence across the designed binder.
Cysteine Count exactly 0, to prevent the formation of off-target disulfide bonds and simplify experimental expression and purification.

To ensure broad exploration of the structural landscape, the remaining candidates were clustered at a TM-score threshold of 0.8, with a single representative from each cluster retained for final selection.

S3.2 Hotspot conditioning analysis

Multiple hotspot combinations were evaluated for both targets to assess the influence of interface residue selection on design success (Table S8). For PDGFR, hotspot sets that included residues A77, A82, and A11—which contribute hydrophobic contacts at the core of the interface—consistently yielded the highest binder rates and affinities. The best-performing combination (NN-PDGFR-6: A139, A77, A82) achieved a 71.1% hit rate with the tightest binder reaching $-\log(K_{\mathrm{D}}) = \mathit{10.03}$, corresponding to sub-100 pM affinity. Conversely, the set restricted to peripheral residues (NN-PDGFR-3: A139, A116, A142, A134) yielded a substantially lower hit rate (31.3%) and weaker affinities, confirming that hotspot selection critically shapes design outcomes.

For PD-L1, overall hit rates were lower (5–18%), consistent with the more challenging, flatter interface. Nevertheless, hotspot conditioning had a marked effect: the combination NN-PDL1-4 (A39, A98, A51) achieved the highest hit rate of 17.9%, while the single-residue set NN-PDL1-2 (A39 alone) yielded only 5.3%. This indicates that even on difficult targets, multi-residue hotspot conditioning concentrating on key interface positions substantially improves design success.

S4 Massive-scale benchmark: extended analysis

S4.1 Large-scale design campaign

Following [35], Manifold Bio selected 127 design targets to define a diverse and biologically relevant benchmark panel for evaluating Proteína-Complexa alongside recent generative and hallucination-based binder design methods. For targets included in prior in vitro studies (e.g., [67]), we used hotspot definitions reported in the literature. For the remaining targets, where rigorously validated hotspots were unavailable for more than 93% of cases, Manifold Bio proposed approximately 10 single-residue hotspots per target and paired each hotspot with distance-based target crops, yielding more than 1,200 formal design tasks.

For each target, we set out to screen 10,000 sequences. For targets with only one known hotspot, this budget was doubled to maintain comparable search breadth. As described in Section 5, approximately half of the total sequence budget was allocated to a diverse hotspot sweep using Proteína-Complexa. The remaining half was

bioRxiv preprint doi: https://doi.org/10.64898/2026.09.12.751118; this version posted September 18, 2026. The copyright holder for this preprint (which was not certified by peer review) is the author/funder, who has granted bioRxiv a license to display the preprint in perpetuity. It is made available under a CC-BY 4.0 International license.

split among baseline comparisons with matched compute budgets, Proteína-Complexa sequence-redesign ablations, and Proteína-Complexa sampling-temperature ablations.

For each task, we ran one instance of Proteína-Complexa with standard beam search and AlphaFold2 interface predicted aligned error (ipAE) as the reward, generating approximately 27,000 candidate binders. We filtered this pool to the top 1,000 candidates ranked by pAE. For each hotspot task, we recorded candidates satisfying all of the following criteria: minimum interface pAE < 2, complex pLDDT > 0.8, ipTM > 0.7, and fewer than 5 backbone Cα clashes with the uncropped target (with a clash defined as any atom pair within 1 Å).

For each target, the hotspot with the largest number of candidates passing these criteria was designated the *top hotspot*. This top-ranked in silico hotspot was used for all baseline experiments shown in Fig. 3c. An analysis of the experimental on-target hit fraction against the in silico hotspot ranking is shown in Supplementary Fig. S7. For targets with only one hotspot, we repeated the search twice and retained the best 1,000 sequences from each run. We did not evaluate samples from hotspots that yielded no in silico success.

In the experiment testing in total 492,636 Proteína-Complexa sequences spanning multiple hotspot combinations for panel-wide target and epitope coverage, to screen a structurally diverse binder set for each target, we pooled all generated binders from hotspots with at least one *in silico* successful design and clustered them at the target level by predicted binder structure. We then filled the evaluation budget using a staged selection procedure that cycled across hotspots and sampled without replacement from these target-level structure clusters. Specifically, we iterated through four ranking regimes in sequence: minimum ipSAE, ipTM, minimum ipAE, and random sampling. Within each regime, the top cluster candidate was selected from previously unselected clusters whenever possible, thereby promoting diversity across both hotspot conditions and predicted binding modes while still prioritizing high-confidence AlphaFold2 predictions (see Supplementary Fig. S6). The resulting screen, including both on-target and off-target hits, is shown in Fig. 3a.

In the experiment testing in total 390,569 sequences from all evaluated method and sequence-design configurations in a compute-matched comparison, we used only the top-ranked in-silico hotspot and we ranked the 1,000 retained Proteína-Complexa candidates by minimum ipSAE and selected the top 400 without clustering. For the baseline methods we similarly chose the top 400 candidates by minimum ipSAE without clustering from the pool of samples generated with the same computational budget across all methods. Hence, the baseline method allocation within the candidate budget for experimental testing was distributed evenly across methods. The same selection procedure was applied to sampling-temperature and sequence-redesign ablations using the top hotspot condition. Minimum ipSAE [58] was chosen here as the primary selection metric to align with recent in vitro practice and to provide a consistent criterion across methods. For sequence-redesign ablations, we followed [18], applying soluble ProteinMPNN for both full-sequence redesign and fixed-interface redesign. For temperature ablations, we started from the default backbone/local latent temperatures of $0.1, 0.1$ and evaluated $0.1, 0.4$, $0.4, 0.1$, and $0.4, 0.4$.

bioRxiv preprint doi: https://doi.org/10.64898/2026.09.12.751118; this version posted September 18, 2026. The copyright holder for this preprint (which was not certified by peer review) is the author/funder, who has granted bioRxiv a license to display the preprint in perpetuity. It is made available under a CC-BY 4.0 International license.

Overall, the campaign required more than 140,000 GPU hours and produced a curated set of 1,015,870 binders that were successfully experimentally tested. Because Proteína-Complexa was run across more than 1,200 simultaneous design tasks, we standardized beam search hyperparameters to generate roughly 27,000 binders per task. Under this setup, 80% of tasks finished within 20–32 GPU hours, with higher costs for longer target crops. To match compute across methods, each baseline received approximately 32 GPU hours per target and hotspot. Because downstream experimental processing introduced modest differences in final tested counts, we report tested sequence counts alongside hit counts and hit rates. For the initial designed set on the 83 solvable baseline targets (Fig. 3c), Proteína-Complexa used 26 GPU hours on average, with 80% of targets under 32 GPU hours and a maximum runtime of approximately 40 GPU hours. Additional baseline implementation details are provided in Supplementary S4.6.

S4.2 Experimental testing: Manifold Bio large-scale all-by-all binder experiment

Protein sourcing.

Recombinant proteins were obtained from commercial vendors (Supplementary Table S6) and accompanied by vendor quality-control documentation (e.g., SDS-PAGE purity and activity/binding checks). Proteins were reconstituted and stored according to manufacturer specifications before use.

Library cloning, phage production, and display.

All binder polypeptide sequences were back-translated using Manifold Bio's internal codon optimization tool and ordered as oligopools (Twist Biosciences). Libraries were cloned into a pADL-100 derivative phagemid (Antibody Design Laboratories) using Gibson Assembly. Insert/vector mixtures were assembled under standard conditions, electroporated into bacterial cells, and assessed for library coverage. Libraries with adequate coverage were prepared and stored at $-80\,^{\circ}$C. Phage display protocols followed [35]. Sequencing libraries were indexed and run on an Illumina NovaSeq X (Broad Clinical Labs, MA). Paired-end reads (2×150 bp) were merged with BBMerge [59]. Mean sequence quality was Q40 (error rate $\approx 10^{-4}$ per base). Only exact matches to designed binder variants were counted.

Computational binder selection and hit calling.

Hits were called from enrichment statistics computed against the pre-selection input library, then classified by the number of distinct screen targets called positive relative to the intended design target. Single-target hits were labeled *on-target specific* (match to design target) or *off-target specific* (single hit against non-design target). Hits spanning two to four targets were labeled *on-target cross-reactive* (design target plus one to three additional targets) or *off-target cross-reactive* (two to four non-design targets).

Read counts were converted to frequencies under a Dirichlet–multinomial model with a symmetric prior ($\alpha = 1$), giving posterior-mean frequency $f_i = (n_i + \alpha)/(N_{\text{reads}} + M\alpha)$ and posterior standard deviation $\sigma_i =$

bioRxiv preprint doi: https://doi.org/10.64898/2026.09.12.751118; this version posted September 18, 2026. The copyright holder for this preprint (which was not certified by peer review) is the author/funder, who has granted bioRxiv a license to display the preprint in perpetuity. It is made available under a CC-BY 4.0 International license.

$\sqrt{f_i(1-f_i)/(N_{\text{reads}}+M\alpha+1)}$ for each of the M binders. The prior acts as Laplace smoothing so that no binder has an estimated frequency of exactly zero, which is required for the ratios below. Frequencies were scaled to concentrations, yielding input concentration c_i with error e_{c_i} and, for each of the k replicate wells r of target group g, output concentration $c_{o,i,g,r}$. For binder i and target group g the enrichment ratio and its propagated error are

$$R_{i,g} = \frac{\bar{c}_{o,i,g}}{c_i}, \qquad \bar{c}_{o,i,g} = \frac{1}{k}\sum_{r=1}^{k} c_{o,i,g,r}, \tag{23}$$

$$e_{R_{i,g}} = R_{i,g}\sqrt{\left(\frac{e_{o,i,g}}{\bar{c}_{o,i,g}}\right)^2 + \left(\frac{e_{c_i}}{c_i}\right)^2}, \qquad e_{o,i,g} = \frac{\text{sd}_r(c_{o,i,g,r})}{\sqrt{k}}. \tag{24}$$

Normalizing to the pre-selection input library is what distinguishes this statistic from a rank threshold on output signal alone: a binder abundant in the output because it was already abundant in the input is not enriched, and a low-input binder that survives selection is recovered even though its absolute output signal is small.

Because the great majority of library members are not selected, the null enrichment level is estimated robustly from the data itself,

$$R_0 = \text{median}_i(R_{i,g}), \qquad \sigma_0 = 1.4826 \times \text{median}_i(|R_{i,g} - R_0|), \tag{25}$$

which is insensitive to contamination by up to 50% true hits. A single global (R_0, σ_0) per sub-library is not adequate, however: $R_{i,g} = \bar{c}_{o,i,g}/c_i$ is inflated by small denominators, so low-abundance binders are systematically over-called against a null dominated by high-abundance ones. We therefore stratify the null by input abundance. Binders are sorted by $\log_{10} c_i$ and divided into B equal-count quantile bins ($B = \min(50, \lfloor n/100 \rfloor)$, at least 3); $R_0^{(b)}$ and $\sigma_0^{(b)}$ are computed per bin by Eq. 25 and linearly interpolated to give per-binder $R_{0,i}$ and $\sigma_{0,i}$, with $\sigma_{0,i}$ floored at 10% of the global value for numerical stability. The null is estimated separately within each sub-library, so sub-libraries of differing quality do not borrow each other's background. The test statistic is the studentized enrichment

$$z_{i,g} = \frac{R_{i,g} - R_{0,i}}{\sqrt{e_{R_{i,g}}^2 + \sigma_{0,i}^2}}, \tag{26}$$

which adds replicate measurement error and null spread in quadrature, so a noisily measured binder must deviate further to reach the same significance. Only right-tail deviations are tested, since depletion below the null is counter-selection rather than binding, and in an earlier two-sided formulation every hit drawn from the depleted tail was off-target:

$$p_{i,g} = 1 - \Phi(z_{i,g}). \tag{27}$$

Magnitude is reported separately as $\text{FC}_{i,g} = \log_2(R_{i,g}/R_{0,i})$.

A binder was called a hit for target group g when $p_{i,g} < 0.05$ and $\text{FC}_{i,g} > 2$, and it passed exactly one target gene across the panel. The magnitude term is required

bioRxiv preprint doi: https://doi.org/10.64898/2026.09.12.751118; this version posted September 18, 2026. The copyright holder for this preprint (which was not certified by peer review) is the author/funder, who has granted bioRxiv a license to display the preprint in perpetuity. It is made available under a CC-BY 4.0 International license.

alongside significance because with $M \sim 10^5$ binders per group a small but highly reproducible ratio can be statistically significant without being biologically meaningful. Because the uniqueness term depends on how many of the 129 targets a binder passed, it is a panel-global property and is evaluated in a single pass over the full panel rather than reconstructed from any one target's statistics.

Called hits were assessed against the negative-control ("empty") wells carried on every plate, which are real outputs of the same selection, amplification and sequencing process with no target protein present and therefore no target-specific enrichment. For each hit we compare its mean output across the target's k replicate wells to its mean across all control wells. Over the 40 highest-yielding target groups of a representative screen (18,536 calls), hits sit a median 4.2× above their own control-well level (interquartile range 3.8–4.8×; 2.0–18.2× across targets, every target at $\geq 2\times$), and for 39 of 40 targets fewer than 4% of calls have a control-well mean that reaches their target mean (median 0.7%). Applying the identical statistic to k control wells drawn as a decoy pseudo-target, on the same binders, yields a median ratio of 0.79× — so the separation is specific to the presence of the target and is not a property of the statistic or of the binders themselves.

Specificity across the panel is the second line of evidence, and it is built into the hit rule rather than applied afterwards: a binder enriched on more than one target gene is not called for any of them. Because the 129 targets are screened simultaneously on the same library, each target's panel-mates act as mutual controls, so a binder that clears the significance and magnitude terms broadly is recognised as non-specific rather than counted once per target.

S4.3 Per-target hotspot analysis

To characterize hotspot-conditioning effects on design success, we analyzed hit distributions across hotspot combinations for targets with multiple tested hotspots (Supplementary Fig. S7). For each target, cells report the fraction of designs yielding on-target specific hits for a given hotspot, with in silico hotspot rank annotated. Targets are ordered by hotspot entropy, which quantifies how evenly success is distributed across hotspot choices. Targets at the top are robust to hotspot choice, whereas targets at the bottom are hotspot-sensitive. Notably, the top-ranked computational hotspot is not always experimentally optimal, supporting multi-hotspot evaluation per target.

S4.4 Model temperature ablation

To assess sensitivity to sampling temperature, we ran an ablation across four backbone/local latent temperature pairs on the 83 solvable baseline targets, designing against each target's top-ranked in silico hotspot. The default setting $(0.1, 0.1)$ yielded the highest on-target hit count (702) and specificity (86.5%), while solving 35 targets (see Supplementary Table S1). Increasing local latent temperature to 0.4 while keeping backbone temperature at 0.1 solved 34 targets and produced fewer total hits (495), although the solved targets were different ones (new/lost columns in table). Each temperature setting uniquely solved 3–6 targets not solved by the others. Specificity remained stable (73.0–86.5%), suggesting elevated temperatures did not materially

bioRxiv preprint doi: https://doi.org/10.64898/2026.09.12.751118; this version posted September 18, 2026. The copyright holder for this preprint (which was not certified by peer review) is the author/funder, who has granted bioRxiv a license to display the preprint in perpetuity. It is made available under a CC-BY 4.0 International license.

reduce selectivity. These results support a mixed-temperature strategy to expand solvable-target coverage while preserving hit quality.

S4.5 Compositional dropout from the phage input pool

Overall, approximately 1.4 million minibinder sequences were designed and ordered across four length-stratified pools. Deep sequencing of the phage input pool before selection recovered 73.2% of designs at one or more reads, resulting in the 1,015,870 successfully tested binders. Two sequence features strongly predicted dropout: sequence length and fractional lysine+glutamate (K+E) content.

Across amino acids, lysine and glutamate showed the strongest negative Spearman correlation with detection (K: $\rho = -0.268$, E: $\rho = -0.217$; Supplementary Fig. S12b). Joint binning by length and K+E fraction (Supplementary Fig. S12a) showed detection rates from 97.9% at 50 aa with K+E < 0.08 to 0.19% at 88 aa with K+E > 0.50.

We tested three possible explanations. (i) Net charge alone does not explain the trend: arginine (+1, $\rho = +0.090$) and aspartate (-1, $\rho = +0.063$) were positively correlated with detection. (ii) Helix propensity alone does not explain the trend: alanine, methionine, and leucine have comparable/high P_α values without similarly negative correlations ($\rho = +0.071, -0.038, -0.050$). (iii) Adenine-rich codons alone do not explain the trend: asparagine and glutamine share related codon motifs yet show no corresponding negative behavior ($\rho = +0.011, +0.145$). No single property recapitulated the K+E dropout pattern.

In a PaCMAP embedding of all ≈1.4 million designs in 20-dimensional amino-acid composition space (Supplementary Fig. S12c,d), undetected designs concentrated in the high-K+E region. Among the 1,015,870 detected designs, input abundance spanned 0.011× to 12.3× the median (2nd–98th percentile; 1092-fold), with lower abundance in the same high-K+E region.

An enrichment of K+E among non-recovered de novo designs was independently observed in a CAR-T proliferation assay [68], where combined helical-region K+E content was the strongest predictor of non-recovery (ROC-AUC = 0.91). This suggests a shared compositional liability across both bacterial phage display and mammalian surface-expression contexts.

S4.6 Details on use of prior methods for benchmarking

BindCraft. We used the code and checkpoints provided in the public BindCraft repository. We followed the default `default_4stage_multimer_hardtarget` configuration for setting and filters. For each target, we used the top-ranked hotspot for the hotspot conditioning of BindCraft. To ensure fair comparison under the constraint of 32 GPU hour computation budget while maintaining a minimum number of samples for meaningful statistical analysis, we adopted the following procedure of sample selection. First, we generated M binders with a 32 GPU hour budget. Second, we re-designed and evaluated the generated binder sequence 8 times using soluble weights of ProteinMPNN [36] and held interface residues fixed. We re-designed 8 times to be consistent with the re-designing protocol of all models benchmarked in this paper. Then

bioRxiv preprint doi: https://doi.org/10.64898/2026.09.12.751118; this version posted September 18, 2026. The copyright holder for this preprint (which was not certified by peer review) is the author/funder, who has granted bioRxiv a license to display the preprint in perpetuity. It is made available under a CC-BY 4.0 International license.

we randomly trim the generated binder set size to N ($N \leq M$) such that the total generation+redesign+evaluation pipeline could fit in the computation budget. After that, we selected the top-1 re-designed sequence from each generated binder based on the best *minimum ipSAE* [58] metric. If $N < 400$ for large targets that required more compute, we backfilled to 400 samples using the best top-2 (or even top-3 if need) re-designed sequences. In this way, we obtained at least 400 samples per target in the final BindCraft sample set.

RFDiffusion. We utilized the open-source implementation of RFdiffusion in the public RFdiffusion repository, and followed the protein-protein interaction (PPI) design settings provided in the official documentation. For each target, we first generated backbone structures and subsequently employed the soluble weights of ProteinMPNN to generate 8 binder sequences per backbone. We performed 8 redesign cycles to maintain consistency with the benchmarking protocol used for all models in this study. A total computational budget of 32 GPU-hours was allocated for the full pipeline, including backbone generation, sequence redesign, and AlphaFold2-based evaluation. From the resulting pool, we selected the top-1 redesigned sequence for each generated binder based on the best *minimum ipSAE* metric, yielding a final set of 400 binders per target.

RFDiffusion3. We adopted the protein binder design protocol following the production settings in the RosettaCommons Foundry repository for RFdiffusion3. The procedure involved an initial generation of binder structures, followed by the application of soluble ProteinMPNN to produce 8 redesigned sequences per structure. We evaluated both the self-generated sequences and the 8 redesigned variants. Under the 32 GPU-hour budget, we selected the top 400 self-generated sequences and the top 400 redesigned sequences based on the *minimum ipSAE* metric. This procedure resulted in a total of 800 binders per target.

BoltzGen. We employed the BoltzGen `protein-anything` protocol to design binders using the code from the public BoltzGen repository. This unified all-atom diffusion approach integrates structure generation (BoltzGen core) and inverse folding (BoltzIF). For each design trajectory, we generated one sequence via Boltz inverse folding and an additional 8 sequences using the soluble weights of ProteinMPNN. We evaluated the self-generated sequences, the Boltz inverse-designed sequences, and the 8 ProteinMPNN-redesigned sequences. The 32 GPU-hour budget encompassed the entire pipeline: self-generation, Boltz inverse design, ProteinMPNN redesign, and the subsequent evaluation of all resulting sequences. Following the standard selection rules based on *minimum ipSAE*, we identified the top 400 sequences from each of the three categories (self-generated, Boltz inverse-designed, and soluble inverse-designed), resulting in a total of 1,200 binders per target.

S5 ActRIIA binder design: experimental methods

Expression and purification protocol. Plasmids encoding the top 200 designs were transformed into *E. coli* BL21(DE3) cells. A single colony was inoculated into 100 mL LB medium supplemented with 100 μg/mL ampicillin and cultured at 37 °C until the optical density at 600 nm (OD_{600}) reached approximately 0.6 (3–4 hours).

bioRxiv preprint doi: https://doi.org/10.64898/2026.09.12.751118; this version posted September 18, 2026. The copyright holder for this preprint (which was not certified by peer review) is the author/funder, who has granted bioRxiv a license to display the preprint in perpetuity. It is made available under a CC-BY 4.0 International license.

The temperature was then reduced to 18 °C, and protein expression was induced with 0.1 mM IPTG. Cultures were incubated at 18 °C for an additional 16 hours. The overall timeline from gene synthesis to purified protein was approximately three weeks.

To obtain high-quality protein samples for downstream analysis, affinity tags were removed for selected constructs. Cell pellets were resuspended in lysis buffer (1× PBS, pH 7.4), lysed by sonication, and centrifuged to remove cell debris. The clarified supernatant was loaded onto a nickel-affinity column for purification. Eluted fractions were treated with TEV protease to remove the affinity tag and subsequently passed through a second nickel column to separate cleaved protein from uncleaved protein and the His-tagged TEV protease. The target protein was then buffer-exchanged into PBS, and protein concentrations were determined prior to surface plasmon resonance (SPR) analysis. A summary of expression yields for both tagged and tagless constructs is provided in Supplementary Table S9; of the 200 designs, 192 expressed solubly and 110 achieved yields exceeding 50 mg/L.

SPR protocol. The surface plasmon resonance (SPR) experiments were conducted on a Biacore 8K+ instrument at 25 °C (both sample and analysis temperature) to characterize the binding kinetics of 80 analytes and a Bimagrumab control to the ligand ActRIIA(20–135)-Avi. The ligand was immobilized on all eight channels of a Streptavidin (SA) sensor chip at a concentration of 0.5 μg/mL with a flow rate of 5 μL/min for 52 seconds. Assays were performed using a running buffer of 10 mM HEPES pH 7.4, 150 mM NaCl, 3 mM EDTA, 0.05% Tween-20, and 2% DMSO. The experimental workflow included 15 start-up cycles to stabilize the surface prior to analysis, and data were collected at a flow rate of 30 μL/min using multi-cycle kinetics (MCK) with association/dissociation times of 90/180 seconds. Two independent SPR runs were performed; binding measurements were largely consistent across both experiments. The only design showing noticeable variability was #51, which exhibited fluctuations in both K_D and R_{max} values, possibly arising from nonspecific surface interactions or partial self-aggregation during the assay. Representative sensorgrams from both runs are shown in Supplementary Fig. S13, and the full Activin A functional dose-response panel across all eight lead designs is shown in Supplementary Fig. S14.

Smad2/3 luciferase reporter assay protocol. A Smad2/3-responsive luciferase reporter assay was performed using a stable HEK293 TGF-β/Activin/Myostatin-responsive reporter cell line in a 384-well format to evaluate the inhibitory activity of candidate binders. Reporter cells were seeded at 10,000 cells per well in 20 μL of DMEM-based culture medium in white, clear-bottom 384-well plates and incubated at 37 °C with 5% CO_2 for approximately 18 h to allow cell attachment. Test proteins, including engineered mini-binder candidates and the reference antibody bimagrumab, were added in 5 μL volumes and pre-incubated with the cells for 4 h. Subsequently, 5 μL of ligand solution was added to stimulate the pathway, using either human Activin A (20 ng/mL final concentration) or GDF8/myostatin (200 ng/mL final concentration). Test molecules were evaluated in three-fold serial dilutions across the plate. Control wells included ligand-stimulated cells (assay medium + ligand) and unstimulated cells (assay medium only) to define maximal activation and basal signaling levels, respectively. After overnight incubation (∼18 h) to allow transcriptional activation of the Smad2/3-responsive reporter,

bioRxiv preprint doi: https://doi.org/10.64898/2026.09.12.751118; this version posted September 18, 2026. The copyright holder for this preprint (which was not certified by peer review) is the author/funder, who has granted bioRxiv a license to display the preprint in perpetuity. It is made available under a CC-BY 4.0 International license.

30 μL of luciferase detection reagent was added to each well, and luminescence was measured using a plate luminometer. Inhibition of pathway signaling by test proteins was quantified as the reduction in luminescence relative to ligand-stimulated control wells.

S6 PAK1 and CK1δ kinase binder design: experimental methods

S6.1 De novo binder design

Candidate binders targeting the PAK1 catalytic domain (PAK1c; UniProt Q13153, amino acids 273–498) or the CK1δ catalytic domain (CK1δc; UniProt P48730, amino acids 9–277) were designed using Proteína-Complexa. For the split-NeoR complementation assay, a library of 50 PAK1c binders (49–74 amino acids) was generated. Binder open reading frames were flanked by constant sequences for downstream PCR amplification and cloned into the split-NeoR selection vector. For CK1δc, 18 short peptide binders (<31 amino acids) were designed and synthesized as biotin-conjugated peptides.

S6.2 Split-NeoR bacterial selection

The split-neomycin resistance (split-NeoR) protein-fragment complementation assay was used to select PAK1 binders based on reconstitution of aminoglycoside phosphotransferase (APH(3′)-II) activity [42, 43]. Binder candidates were fused to the C-terminal fragment of NeoR, and PAK1c was fused to the N-terminal fragment; both fusion proteins were co-expressed in *E. coli* from a single plasmid. The pooled binder library was transformed into NEB Stable Competent *E. coli* (New England Biolabs, C3040) by chemical transformation. Transformants were plated on LB agar without kanamycin (input control) or LB agar supplemented with 25 μg/mL kanamycin (selection) and incubated overnight. Colonies from both conditions were pooled and plasmid DNA was isolated by miniprep for downstream NGS analysis.

S6.3 Next-generation sequencing and enrichment analysis

Binder-encoding amplicons were prepared by PCR using KAPA HiFi 2× ReadyMix (Roche, KK2601) with library-specific primers flanking the binder open reading frames. PCR conditions were 98 °C for 3 min; 15 cycles of 98 °C for 20 s, 65 °C for 15 s, and 72 °C for 30 s; and 72 °C for 1 min. Template DNA was normalized to 3.0 ng/μL (30 ng input per reaction). Amplicon libraries were submitted for paired-end sequencing (Azenta AmpliconEZ). Reads were mapped to the reference library using a 30 bp ORF-prefix matching strategy anchored to the 5′ constant sequence (5′-`CACTCAGGGTCCGGT`-3′), achieving mapping rates of 91.7–94.7% across samples. Raw read counts were normalized to counts per million (CPM). Enrichment was calculated as $\log_2$ fold change:

$$\log_2((\text{selection CPM} + 1) \,/\, (\text{input CPM} + 1)) \,,$$

bioRxiv preprint doi: https://doi.org/10.64898/2026.09.12.751118; this version posted September 18, 2026. The copyright holder for this preprint (which was not certified by peer review) is the author/funder, who has granted bioRxiv a license to display the preprint in perpetuity. It is made available under a CC-BY 4.0 International license.

where a pseudocount of 1 CPM was added to avoid division by zero. To classify binders as enriched or depleted, a two-component Gaussian mixture model (GMM) was fitted to the $\log_2$ fold-change values using expectation-maximization. Two components were chosen based on the observed bimodal distribution. Each binder was assigned to the component with the higher posterior probability, and the decision boundary was defined as the $\log_2$ fold-change value at which the posterior probabilities of the two components were equal.

S6.4 Structure prediction

Predicted structures of binder–PAK1 complexes were generated using AlphaFold2 Multimer (v2.3.2) with five models and five seeds per model (25 predictions total per complex). The combined interface predicted TM-score and predicted TM-score (ipTM+pTM) from the best-ranked model was used as the metric of predicted binding confidence.

S6.5 GST-tagged protein purification

GST-tagged CK1δc was expressed from pGEX-GST-CK1δ as an N-terminal fusion in BL21(DE3) *E. coli*, induced with 0.1 mM IPTG, and grown at 20 °C for two nights. Cells were lysed by sonication in lysis buffer (50 mM Tris pH 7.5, 100 mM NaCl, 1 mM EDTA, 1 mM EGTA, 2% IGEPAL CA-630, 3 mM DTT, and protease inhibitors) and clarified by centrifugation and 0.45 μm filtration. Proteins were purified using glutathione-Sepharose resin (Glutathione Sepharose™ 4B, Cytiva) at 4 °C and eluted in 50 mM Tris pH 7.5, 150 mM NaCl, 3 mM DTT, and 25 mM reduced glutathione, as previously described [69]. Protein-containing fractions were identified by Bradford assay, pooled, concentrated by ultrafiltration with PBS washes, and stored at −80 °C.

S6.6 Biotinylated peptide synthesis and streptavidin bead pull-down

Biotinylated peptides were synthesized on a MultiPep 1 automated peptide synthesizer (CEM Corporation) using standard Fmoc solid-phase chemistry. Biotin was incorporated at the N-terminus via a biotinylated lysine followed by two glycine spacers. For pull-down assays, all steps were carried out at 4 °C unless otherwise noted. Streptavidin magnetic beads (Pierce™ Streptavidin Magnetic Beads) were washed three times with 500 μL Bead Wash Buffer (BWB; 25 mM HEPES pH 7.4, 150 mM NaCl, 0.01% Tween-20, and 1 mM DTT). For peptide capture, 17 μL of bead slurry was incubated with 1 mL of 3 μM biotinylated peptide with end-over-end mixing for 30–45 min at 4 °C, then washed three times for 3 min with 500 μL BWB. Purified GST-tagged kinase domain (0.75 μg) was added to peptide-loaded beads in 1 mL BWB+ (BWB supplemented with protease inhibitor) and incubated for 30–40 min at 4 °C with end-over-end rotation. Beads were washed four times with 500 μL BWB+ (10 min each). Bound proteins were eluted in 20 μL of 2× Laemmli Sample Buffer (Bio-Rad) containing β-mercaptoethanol at 95 °C for 5 min.

bioRxiv preprint doi: https://doi.org/10.64898/2026.09.12.751118; this version posted September 18, 2026. The copyright holder for this preprint (which was not certified by peer review) is the author/funder, who has granted bioRxiv a license to display the preprint in perpetuity. It is made available under a CC-BY 4.0 International license.

S6.7 Co-immunoprecipitation

HEK293T cells were seeded in 12-well plates and transfected 24 hours later using PEI MAX 40000 (Polyethylenimine HCl MAX, Linear, Mw 40,000; Kyfora Bio, 24765-100). Each well received 500 ng of binder plasmid (pDS261: pCAG-GFP-3×Flag-P2A-3×HA-binder) and 500 ng of bait plasmid (pDS217: pCAG-PAK1-Myc), with PEI MAX at 20 μL per condition. DNA–PEI MAX complexes were formed in Opti-MEM for 15 min at room temperature and added to cells. Medium was replaced 6 hours post-transfection; cells were harvested 48 hours post-transfection. Cells were washed once with cold PBS and lysed in 300 μL of lysis buffer (20 mM HEPES pH 7.4, 150 mM NaCl, 0.5% NP-40, and protease inhibitor cocktail) on ice for 5–10 min. Lysates were clarified by centrifugation (15,000×g, 10 min, 4 °C), and 5–10% of clarified lysate was saved as input. HA-tagged proteins were immunoprecipitated using 15 μL ChromoTek HA-Trap Magnetic Agarose (Proteintech, cat. no. atma) with rotation for 90 min at 4 °C. Beads were washed three times with 500 μL wash buffer (20 mM HEPES pH 7.4, 150 mM NaCl, and 0.1% NP-40). Bound proteins were eluted in 30 μL 2× Laemmli sample buffer at 95 °C for 5 min. An empty pDS261 vector served as the negative control. Three independent biological replicates were performed.

S6.8 Western blotting

For PAK1 co-IP experiments, input and eluate samples were resolved by SDS-PAGE on Mini-PROTEAN precast gels (4–20%; Bio-Rad, 4561096) and transferred to membranes. Membranes were probed with anti-Myc chicken polyclonal antibody (Invitrogen, A-21281) to detect Myc-PAK1, followed by IRDye 800CW donkey anti-chicken IgY secondary antibody. Anti-Flag antibody was used to detect GFP-Flag binder expression (IRDye 680RD secondary, 700 nm channel). Blots were imaged using a LI-COR Odyssey system with simultaneous dual-color detection (700 and 800 nm channels). For CK1δ pull-down experiments, proteins were transferred to membrane and probed with anti-GST antibody (Bethyl Laboratories GST Tag Polyclonal Antibody, HRP, 1:1000). Standard western blot procedures were followed for blocking, primary antibody incubation, and chemiluminescent detection (SuperSignal™ West Femto Maximum Sensitivity Substrate).

S6.9 Microscale thermophoresis binding assays

Purified CK1δ kinase domain was fluorescently labeled in PBS (pH 7.4) using the RED-NHS 2nd Generation Protein Labeling Kit (NanoTemper Technologies, cat. no. MO-L011) according to the manufacturer's protocol. Microscale thermophoresis (MST) binding experiments were performed on a Monolith X instrument (NanoTemper Technologies) equipped with a Nano RED detector. Labeled CK1δ was diluted to 80 nM in assay buffer (0.14% Pluronic F-127 in PBS, pH 7.4). Peptide binders were prepared as 16-point, twofold serial dilution series in assay buffer, with top concentrations of 100 μM for CK3 and CK16 and 200 μM for CK9. Each peptide dilution was mixed 1:1 (v/v) with labeled CK1δ and incubated for 15–30 min at room temperature, protected from light, before loading.

bioRxiv preprint doi: https://doi.org/10.64898/2026.09.12.751118; this version posted September 18, 2026. The copyright holder for this preprint (which was not certified by peer review) is the author/funder, who has granted bioRxiv a license to display the preprint in perpetuity. It is made available under a CC-BY 4.0 International license.

Samples were loaded into Monolith Premium Capillaries (NanoTemper Technologies, cat. no. MO-K025) and analyzed at 25 °C using the 670 nm detection channel and a 1.5 s retention time. Binding was quantified using normalized fluorescence (F_{norm}), defined as the ratio of fluorescence intensity in the heated region after IR-laser activation to the pre-heating baseline fluorescence (López-Méndez et al., 2021). Averaged F_{norm} values were plotted against $\log_{10}([\text{ligand}])$ and fitted by nonlinear least-squares regression using the Levenberg–Marquardt algorithm to a three-parameter logistic model with the Hill slope fixed at 1. Dissociation-constant (K_{d}) measurements were performed in triplicate for each binder. Capillaries with fluorescence counts outside the 500–1,400 range or with excessive aggregation were excluded before curve fitting.

S6.10 Image quantification and statistical analysis

Band intensities were quantified using Fiji/ImageJ. For PAK1 co-IP experiments, co-IP efficiency was calculated as the ratio of anti-Myc signal in the eluate to the anti-Myc signal in the input (elution/input ratio), then normalized to the empty-vector control within each replicate to obtain fold-enrichment values. Statistical significance was assessed by one-sample t-test (two-tailed) against a null hypothesis of fold enrichment $= 1.0$. Data are presented as mean $\pm$ SD from three independent biological replicates. For CK1δ pull-down experiments, samples were normalized to the scrambled control for their respective run. Statistical significance was assessed by one-sample t-test (one-tailed) against a null hypothesis of fold enrichment $= 1.0$. Data are presented as mean $\pm$ SD from three to four independent biological replicates.

S7 Carbohydrate binder design: experimental methods

S7.1 Design generation and filtering

Candidate binders targeting the type II A pentasaccharide were generated using Proteína-Complexa conditioned on the sugar ligand structure. To promote precise hydrogen-bonding networks and deep pocket burial—both critical for overcoming the large desolvation penalty of carbohydrate binding—inference-time rewards included pocket burial scores and hydrogen-bond counts at the binder–ligand interface. Twenty-four candidates were selected for expression testing; 23 designs, all smaller than 10 kDa, advanced to the binding screen reported in the main text (Supplementary Fig. S15).

S7.2 Protein cloning and expression

The 24 initial candidates were gene-optimized for *E. coli* expression and ordered as gene strings with 20 bp overhangs compatible with the acceptor vector T7-pRSF. The T7-pRSF vector was linearized by PCR, and residual circular template was removed by DpnI (NEB) digestion. Linear product was purified by SPRI beads (Beckman) and eluted at a concentration of 50 ng/μL. Genes were ligated into the vector using Gibson

bioRxiv preprint doi: https://doi.org/10.64898/2026.09.12.751118; this version posted September 18, 2026. The copyright holder for this preprint (which was not certified by peer review) is the author/funder, who has granted bioRxiv a license to display the preprint in perpetuity. It is made available under a CC-BY 4.0 International license.

assembly and transformed into BL21(DE3) cells (NEB) under kanamycin selection. Colonies were verified by whole-plasmid sequencing.

Cells were grown at 37 °C in 5 mL LB-kanamycin medium to an OD_{600} of 0.4–0.8, at which point expression was induced with 1 mM IPTG. Cultures were transferred to 20 °C with agitation at 1,000 rpm on a flat shaker-incubator, and expression was carried out for 14 hours. Cells were pelleted by centrifugation (5 min, 4,000×g) and lysed in 2 mL lysis buffer (10× BugBuster (Millipore), diluted in PBS supplemented with $CaCl_2$ and $MgCl_2$ (Thermo)). Lysates were agitated at 1,000 rpm for 30 min at 20 °C and clarified by centrifugation (10 min, 4,000×g).

The supernatant was collected and incubated with Ni-NTA agarose beads (200 μL slurry per protein) with agitation at 1,000 rpm for 30 min at 20 °C. After centrifugation (3 min, 300×g), the supernatant was discarded and the bead pellet washed with 1 mL PBS. Proteins were eluted in 200 μL elution buffer (PBS, 250 mM imidazole) with agitation for 10 min. The eluate was subjected to two rounds of buffer exchange into PBS using 3 kDa size-exclusion filters (Amicon), with 10-fold dilution and re-concentration per round. Final protein concentrations, quantified by A_{280} (NanoDrop), ranged from 0.8 to 3.5 mg/mL. Proteins were stored at 4 °C and assayed within three days of purification.

S7.3 Red blood cell agglutination assay

Commercially sourced reagent red blood cells (types A_2 and B) were tested separately. Each neat stock was diluted 1:100 in PBS and washed once by centrifugation (3 min, 300×g) without further dilution. A Ni-NTA agarose bead suspension was prepared at a ratio of 2:48 (neat resin:PBS, v/v). For each sample and replicate, 50 μL of bead suspension was loaded with 2 μL of purified binder solution, and 50 μL of the relevant RBC suspension was added. Negative controls included RBCs + beads (no protein), RBCs + protein (no beads), and beads + protein (no RBCs). Samples were incubated in U-bottom 96-deep-well plates (Greiner), with a separate plate per replicate. After 10 min, samples were inspected macroscopically and microscopically for visible agglutination. After 2 hours of incubation, samples were imaged using an EVOS-FL microscope at the region of highest bead density per well, then agitated and OD_{600} measured using a plate reader (SpectraMax). Data are plotted as mean ± standard deviation of three independent replicates. A construct containing the B-binding CBM51 domain served as the positive control, and RBCs + beads without protein served as the negative control baseline.

S7.4 Glycan–DNA conjugation for BLI

To produce a glycan conjugate of sufficient molecular weight to elicit a detectable biolayer interferometry (BLI) response, the blood group A pentasaccharide (type II; Biosynth) was conjugated to a modified DNA oligonucleotide. An oligonucleotide bearing three amine-modified positions ([AmC6]GA[AmC6dT]GA[AmC6dT]CTC-GACGCTCTCCCTTATGCGACTCC; Integrated DNA Technologies) was mixed with the pentasaccharide at a 1:5 molar ratio in 50% DMSO containing 100 mM 2-picoline borane (Sigma). The reductive amination reaction was incubated for 3 hours

bioRxiv preprint doi: https://doi.org/10.64898/2026.09.12.751118; this version posted September 18, 2026. The copyright holder for this preprint (which was not certified by peer review) is the author/funder, who has granted bioRxiv a license to display the preprint in perpetuity. It is made available under a CC-BY 4.0 International license.

at 37 °C. The product was purified and buffer-exchanged into PBS using PD-10 columns (Sephadex G-25, 3 mL elution). Recovered DNA yield was estimated by A_{260} (NanoDrop).

S7.5 Biolayer interferometry

BLI experiments were performed on an Octet K2 (Sartorius) two-channel system using HIS1K biosensors (Sartorius, 18-5120). Interactions were measured in flat black 96-well plates (Nunc; 200 μL/well) with shaking at 1,000 rpm. The assay sequence consisted of: baseline (PBS, 120 s), loading (protein in PBS at 0.1 mg/mL, 120 s), baseline (PBS, 120 s), association (four sequential steps of 120 s each at glycan–DNA conjugate concentrations of 2, 4, 6, and 8 μM in PBS), and dissociation (PBS, 120 s). A reference sensor was treated identically but without protein in the loading step. Data were acquired at 5 Hz and reference-subtracted.

S7.6 Circular dichroism

CD spectroscopy was performed on an Aviv 410 CD spectrometer using a quartz cuvette (1 mm path length). Design NV15 (S15) was loaded at a final concentration of 0.25 mg/mL in PBS. A wavelength scan (200–250 nm) was carried out at 25 °C with a bandwidth of 1.00 nm to confirm the presence of secondary structure elements. For thermal stability analysis, CD signal was monitored at 222 nm with a bandwidth of 10 nm. Dynode voltage was recorded concurrently to check for aggregation, which was not observed. An initial scan from 40 to 95 °C identified the inflection region, followed by a higher-resolution scan from 70 to 98 °C. Secondary structure elements remained stable beyond 100 °C, precluding accurate determination of a melting temperature within the accessible range.

bioRxiv preprint doi: https://doi.org/10.64898/2026.09.12.751118; this version posted September 18, 2026. The copyright holder for this preprint (which was not certified by peer review) is the author/funder, who has granted bioRxiv a license to display the preprint in perpetuity. It is made available under a CC-BY 4.0 International license.

S8 Supplementary figures

The following figures supplement the main text and Supplementary Methods.

bioRxiv preprint doi: https://doi.org/10.64898/2026.09.12.751118; this version posted September 18, 2026. The copyright holder for this preprint (which was not certified by peer review) is the author/funder, who has granted bioRxiv a license to display the preprint in perpetuity. It is made available under a CC-BY 4.0 International license.

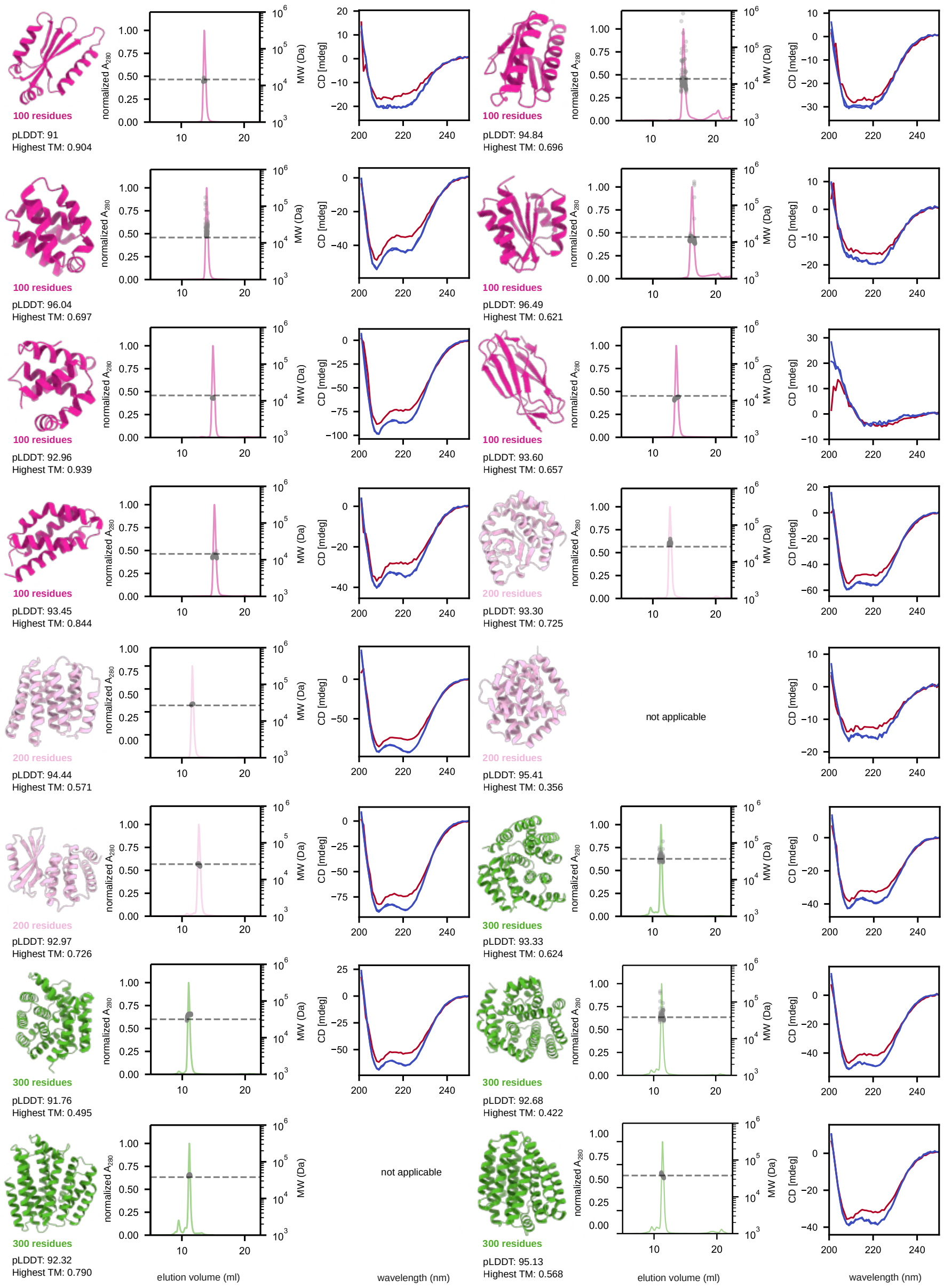

Fig. S1 Biophysical characterization of designed monomeric folds: 100–300 residue designs. For each design, the predicted structure model (left), SEC profile overlaid with MALS-determined molecular weight (middle), and CD spectra at 20 °C (blue) and 95 °C (red) (right) are shown. Protein models are colored according to chain length. The average pLDDT from AF2 single-sequence predictions and the Foldseek TM-score to the closest known fold are reported for each design.

bioRxiv preprint doi: https://doi.org/10.64898/2026.09.12.751118; this version posted September 18, 2026. The copyright holder for this preprint (which was not certified by peer review) is the author/funder, who has granted bioRxiv a license to display the preprint in perpetuity. It is made available under a CC-BY 4.0 International license.

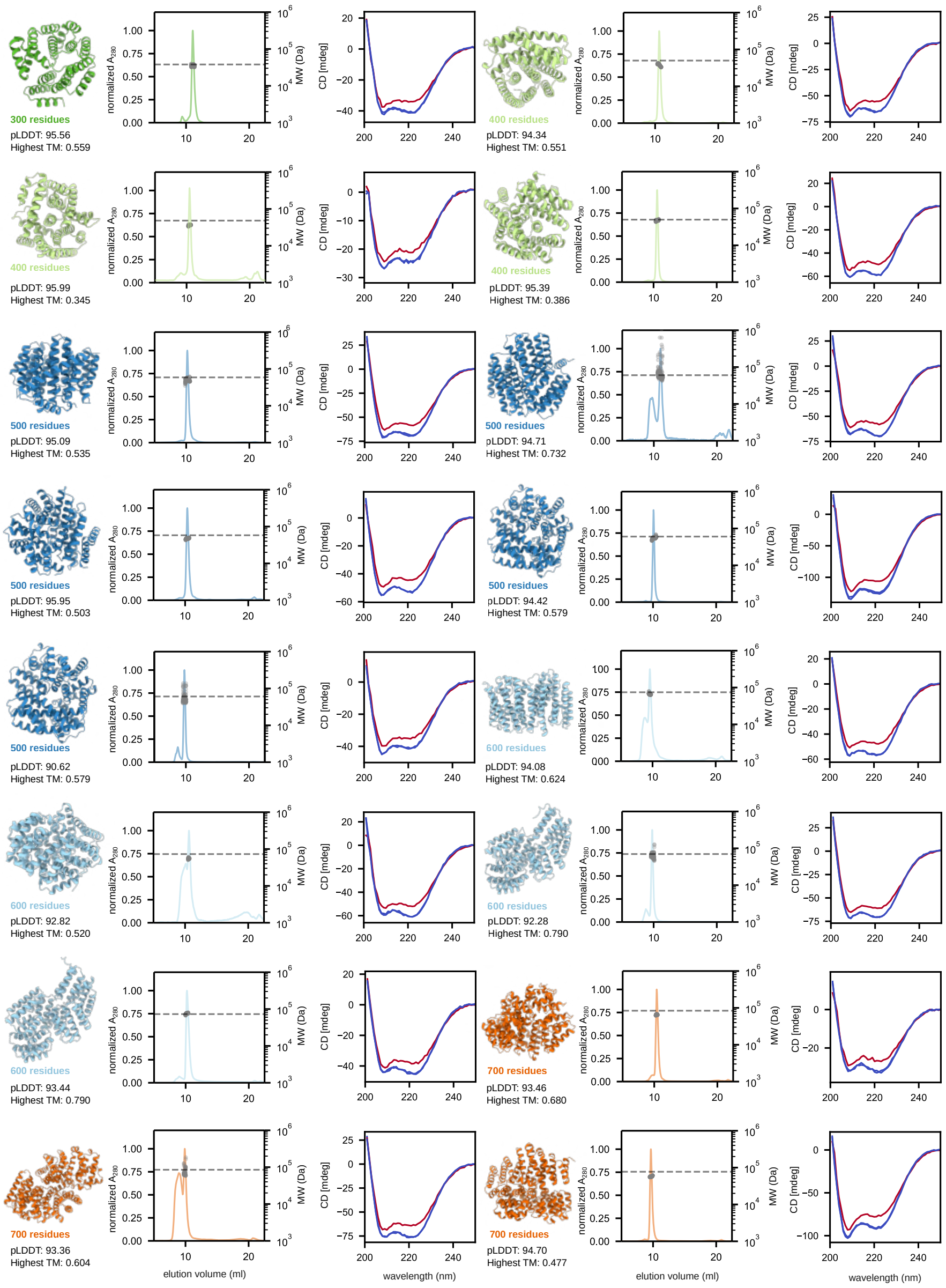

Fig. S2 Biophysical characterization of designed monomeric folds: 300–700 residue designs. Layout as in Supplementary Fig. S1.

bioRxiv preprint doi: https://doi.org/10.64898/2026.09.12.751118; this version posted September 18, 2026. The copyright holder for this preprint (which was not certified by peer review) is the author/funder, who has granted bioRxiv a license to display the preprint in perpetuity. It is made available under a CC-BY 4.0 International license.

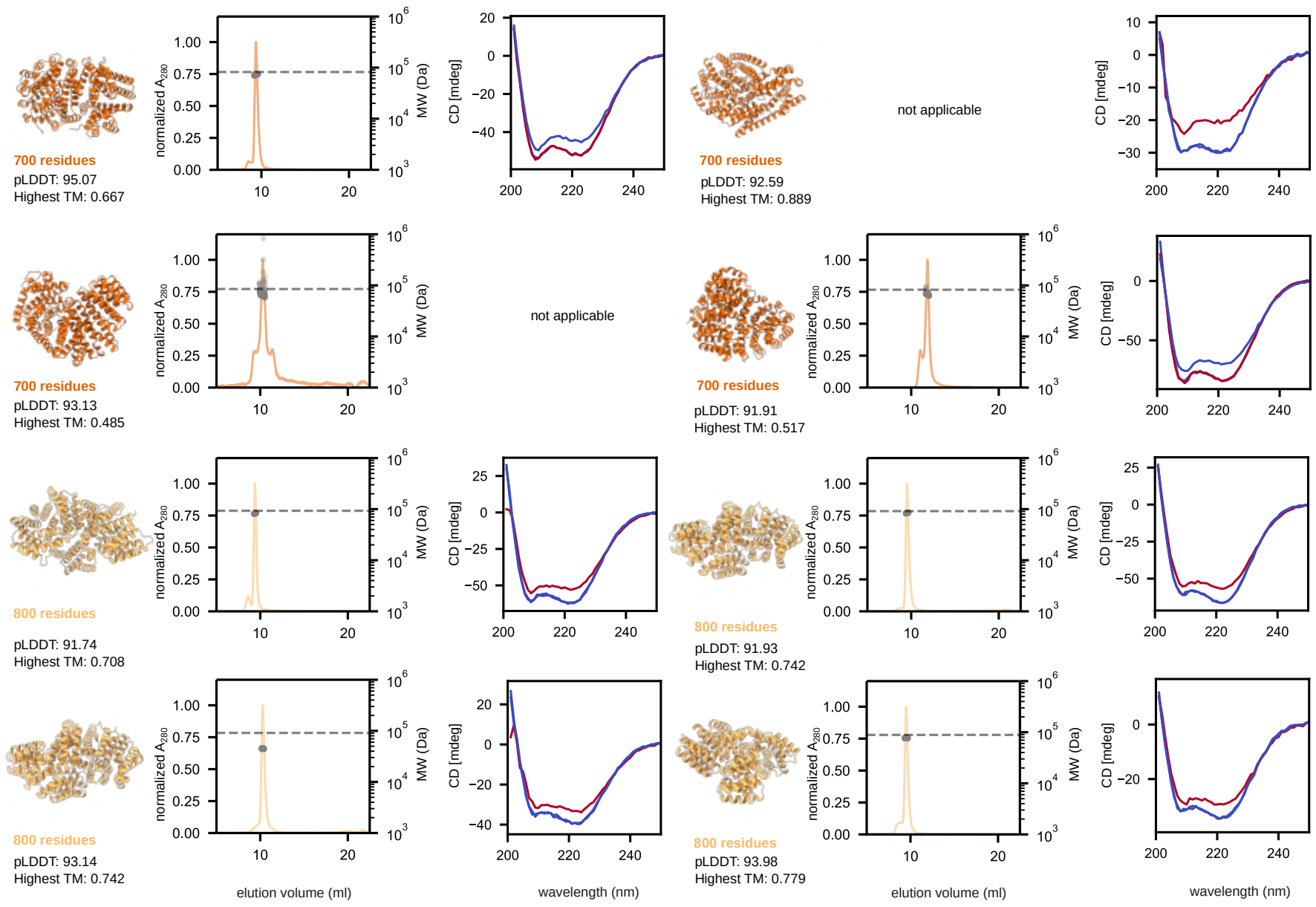

Fig. S3 Biophysical characterization of designed monomeric folds: 700–800 residue designs. Layout as in Supplementary Fig. S1.

bioRxiv preprint doi: https://doi.org/10.64898/2026.09.12.751118; this version posted September 18, 2026. The copyright holder for this preprint (which was not certified by peer review) is the author/funder, who has granted bioRxiv a license to display the preprint in perpetuity. It is made available under a CC-BY 4.0 International license.

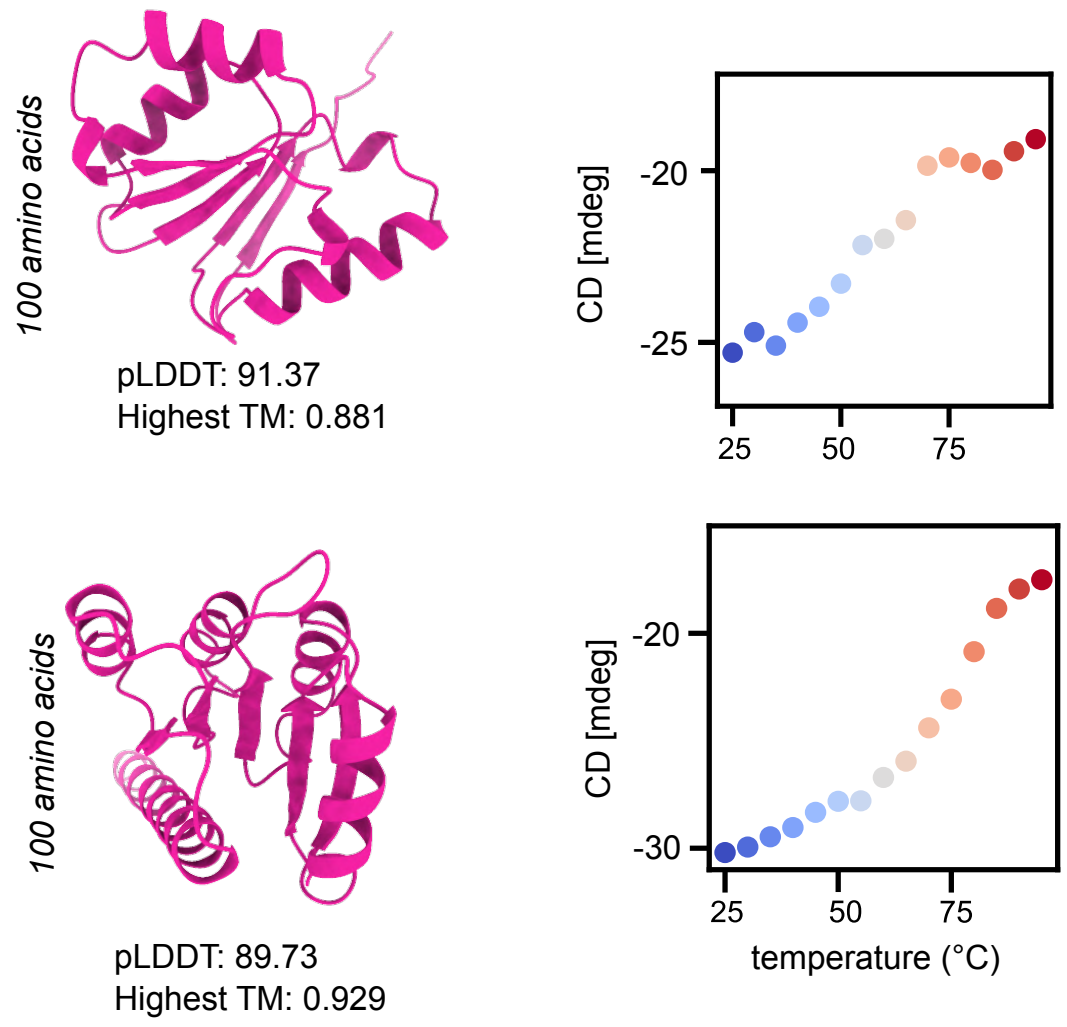

Fig. S4 Biophysical characterization of monomers generated at high sampling temperature. Predicted structures and circular-dichroism thermal denaturation curves are shown for the two designs, from a selected set of 13 that expressed solubly. Both proteins undergo cooperative thermal transitions below 95 °C. The mean pLDDT from MSA-based AlphaFold2 predictions and the Foldseek TM-score to the closest known fold are reported for each design.

bioRxiv preprint doi: https://doi.org/10.64898/2026.09.12.751118; this version posted September 18, 2026. The copyright holder for this preprint (which was not certified by peer review) is the author/funder, who has granted bioRxiv a license to display the preprint in perpetuity. It is made available under a CC-BY 4.0 International license.

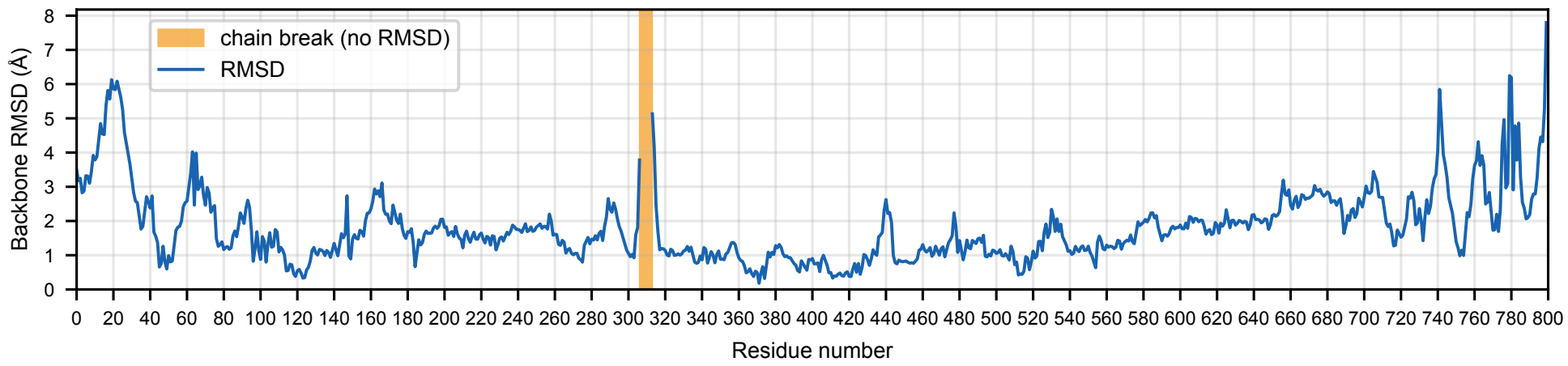

Fig. S5 Root mean square deviation (RMSD) between crystal structure and design model. Plot shows the average per-residue RMSD between the backbone atom positions of the experimentally determined crystal structure and the generated design by La-Proteína. Residues 307–312 were not experimentally resolved.

bioRxiv preprint doi: https://doi.org/10.64898/2026.09.12.751118; this version posted September 18, 2026. The copyright holder for this preprint (which was not certified by peer review) is the author/funder, who has granted bioRxiv a license to display the preprint in perpetuity. It is made available under a CC-BY 4.0 International license.

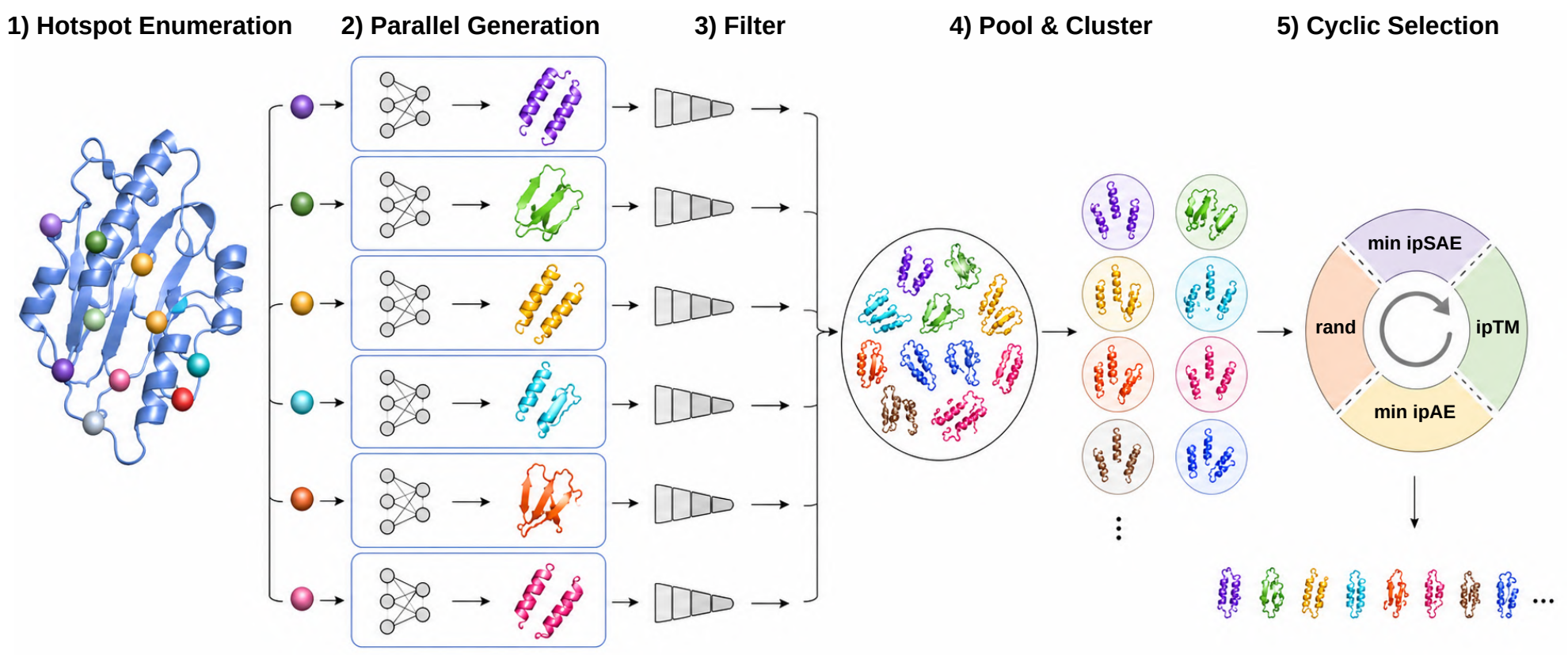

Fig. S6 Massive-scale design and selection pipeline. The per-target procedure used to select the 492,636 Proteína-Complexa-generated sequence subset across the 127-target panel with diverse hotspots. (1) enumeration of candidate interface hotspot combinations; (2) parallel generation of candidates by beam-search-guided latent generative search; (3) in-silico filtering on interface confidence and physicochemical metrics; (4) pooling and structural clustering to remove redundancy; and (5) cyclic selection that rotates ranking criteria (minimum interface pAE, interface pTM, minimum interface pSAE and random selection) across clusters to assemble a diverse, size-matched set of designs for synthesis and multiplexed phage-display screening.

bioRxiv preprint doi: https://doi.org/10.64898/2026.09.12.751118; this version posted September 18, 2026. The copyright holder for this preprint (which was not certified by peer review) is the author/funder, who has granted bioRxiv a license to display the preprint in perpetuity. It is made available under a CC-BY 4.0 International license.

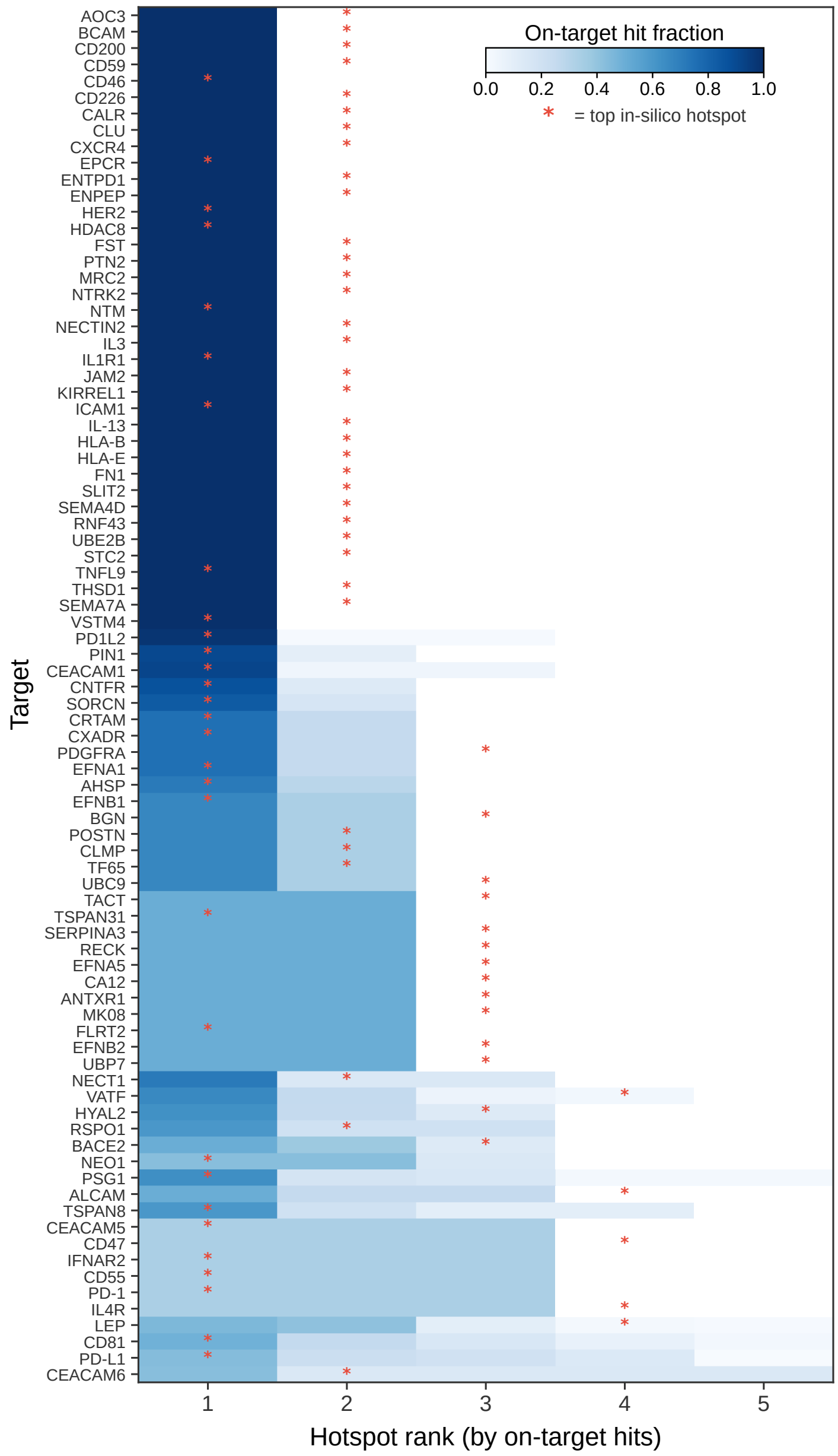

Fig. S7 Per-target hotspot hit concentration analysis. Heatmap of on-target hit fraction across the top five hotspots for targets with at least one on-target hit. Targets are ordered by hotspot entropy. Red asterisks denote the top-ranked in-silico hotspot per target. Hit fractions are normalized over the top 10 hotspots per target.

bioRxiv preprint doi: https://doi.org/10.64898/2026.09.12.751118; this version posted September 18, 2026. The copyright holder for this preprint (which was not certified by peer review) is the author/funder, who has granted bioRxiv a license to display the preprint in perpetuity. It is made available under a CC-BY 4.0 International license.

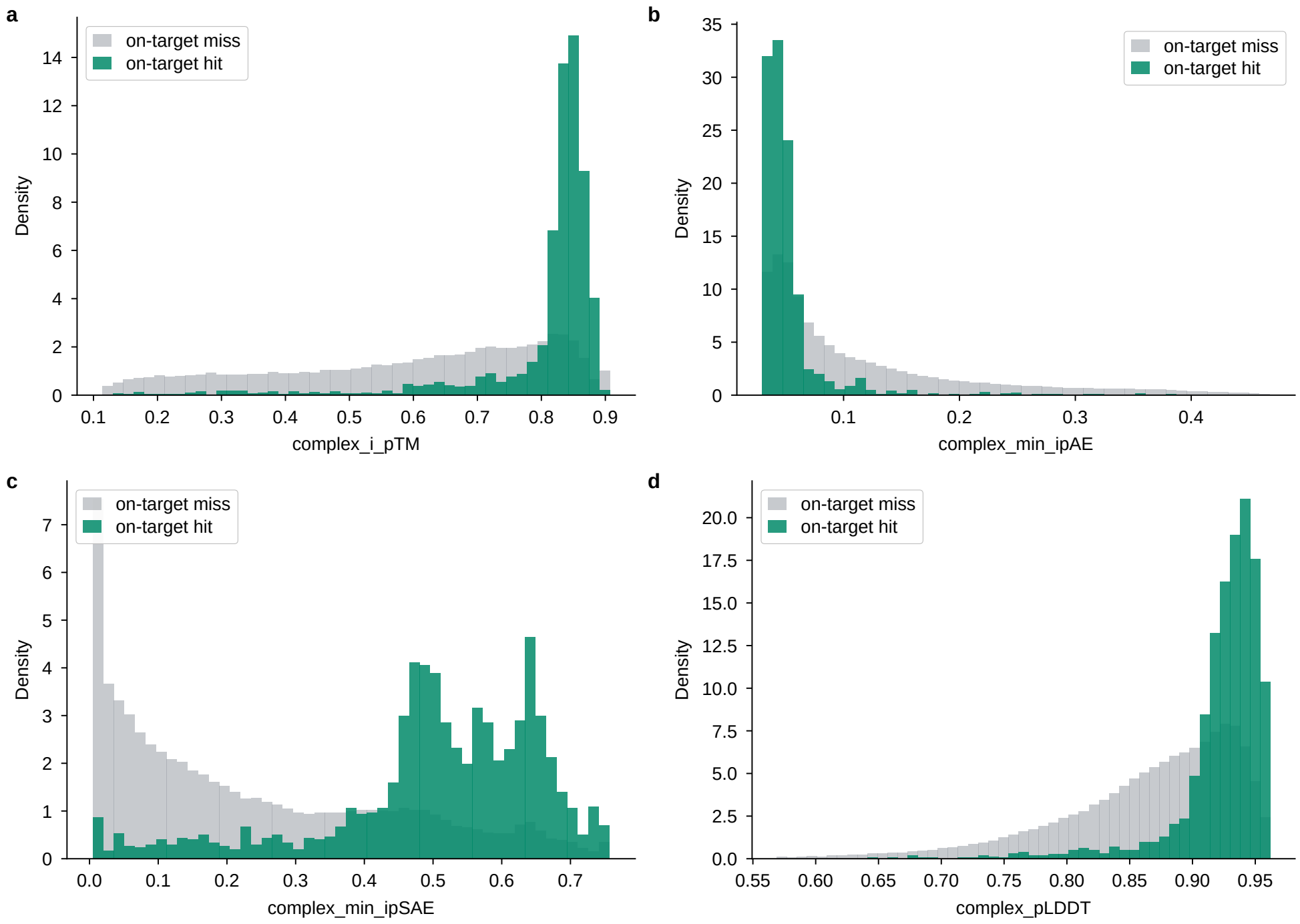

Fig. S8 In-silico confidence scores separate experimental hits from misses. Distributions of AlphaFold2-Multimer complex confidence metrics — interface pTM (**a**), minimum interface pAE (**b**), minimum interface pSAE (**c**) and complex pLDDT (**d**) — for Proteína-Complexa designs classified as on-target hits (green) versus misses (grey) in the phage-display benchmark. Higher interface pTM, interface pSAE and pLDDT and lower interface pAE are enriched among experimental hits, indicating that these in-silico metrics are predictive of experimental success.

bioRxiv preprint doi: https://doi.org/10.64898/2026.09.12.751118; this version posted September 18, 2026. The copyright holder for this preprint (which was not certified by peer review) is the author/funder, who has granted bioRxiv a license to display the preprint in perpetuity. It is made available under a CC-BY 4.0 International license.

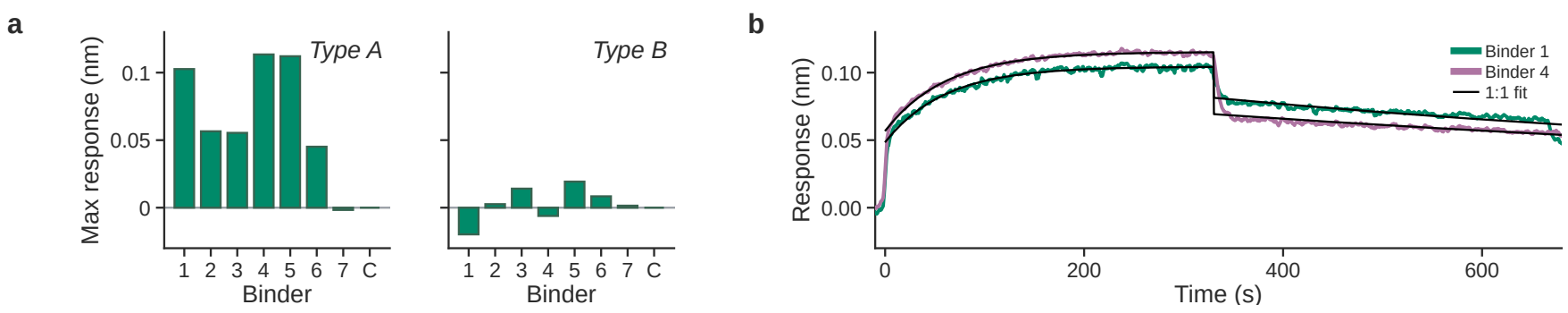

Fig. S9 BLI specificity screen of carbohydrate-binder designs. a, Maximum biolayer-interferometry (BLI) association response of BLI-tested FP-positive designs, and a control (C), challenged with the type-A (left) versus the type-B (right) glycan conjugate, measured in *E. coli* lysate. Most designs respond strongly to the type-A glycan with little or no response to type-B, consistent with antigen-preferential binding. **b**, Representative association/dissociation sensorgrams for designs 1 and 4 against the type-A glycan, with 1:1 kinetic fits overlaid (black). Because the assay was run in crude lysate, these data are reported as supporting rather than definitive evidence of antigen specificity (see main text).

bioRxiv preprint doi: https://doi.org/10.64898/2026.09.12.751118; this version posted September 18, 2026. The copyright holder for this preprint (which was not certified by peer review) is the author/funder, who has granted bioRxiv a license to display the preprint in perpetuity. It is made available under a CC-BY 4.0 International license.

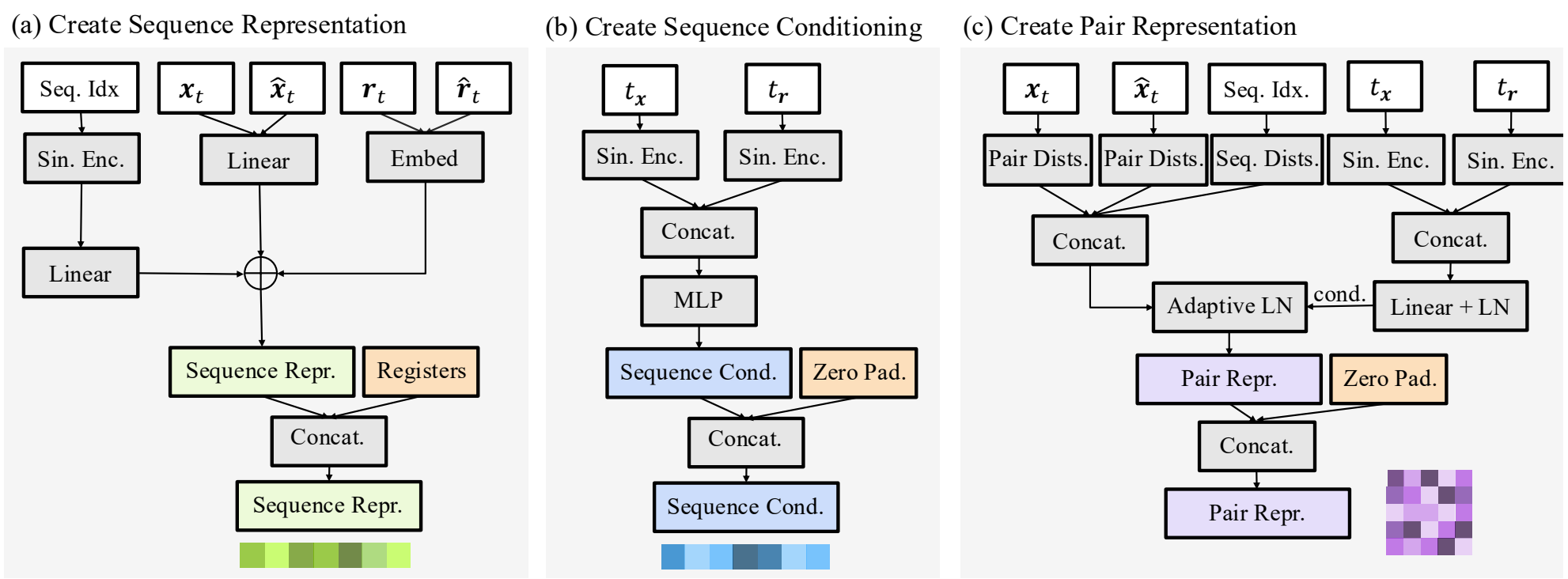

Fig. S10 Construction of the inputs to the conditional flow denoiser. a, Sequence indices, coordinate channels, and latent or target-specific features are encoded and combined to form the initial sequence representation. Learned register tokens are then appended to the physical binder and target tokens. **b**, The backbone and latent interpolation times are sinusoidally encoded, concatenated, and passed through an MLP to produce the sequence conditioning representation. Zero-valued entries are appended for the register tokens. **c**, Pairwise coordinate distances, sequence separations, and the two time embeddings are combined to construct the initial pair representation. The geometric and sequence-derived pair features are adaptively normalized using the time conditioning before being concatenated with a transformed time embedding. Pair entries involving register tokens are zero-padded. The latent channel denoted by r and t_r in the schematic corresponds to $\mathbf{z}$ and t_z in the main text.

bioRxiv preprint doi: https://doi.org/10.64898/2026.09.12.751118; this version posted September 18, 2026. The copyright holder for this preprint (which was not certified by peer review) is the author/funder, who has granted bioRxiv a license to display the preprint in perpetuity. It is made available under a CC-BY 4.0 International license.

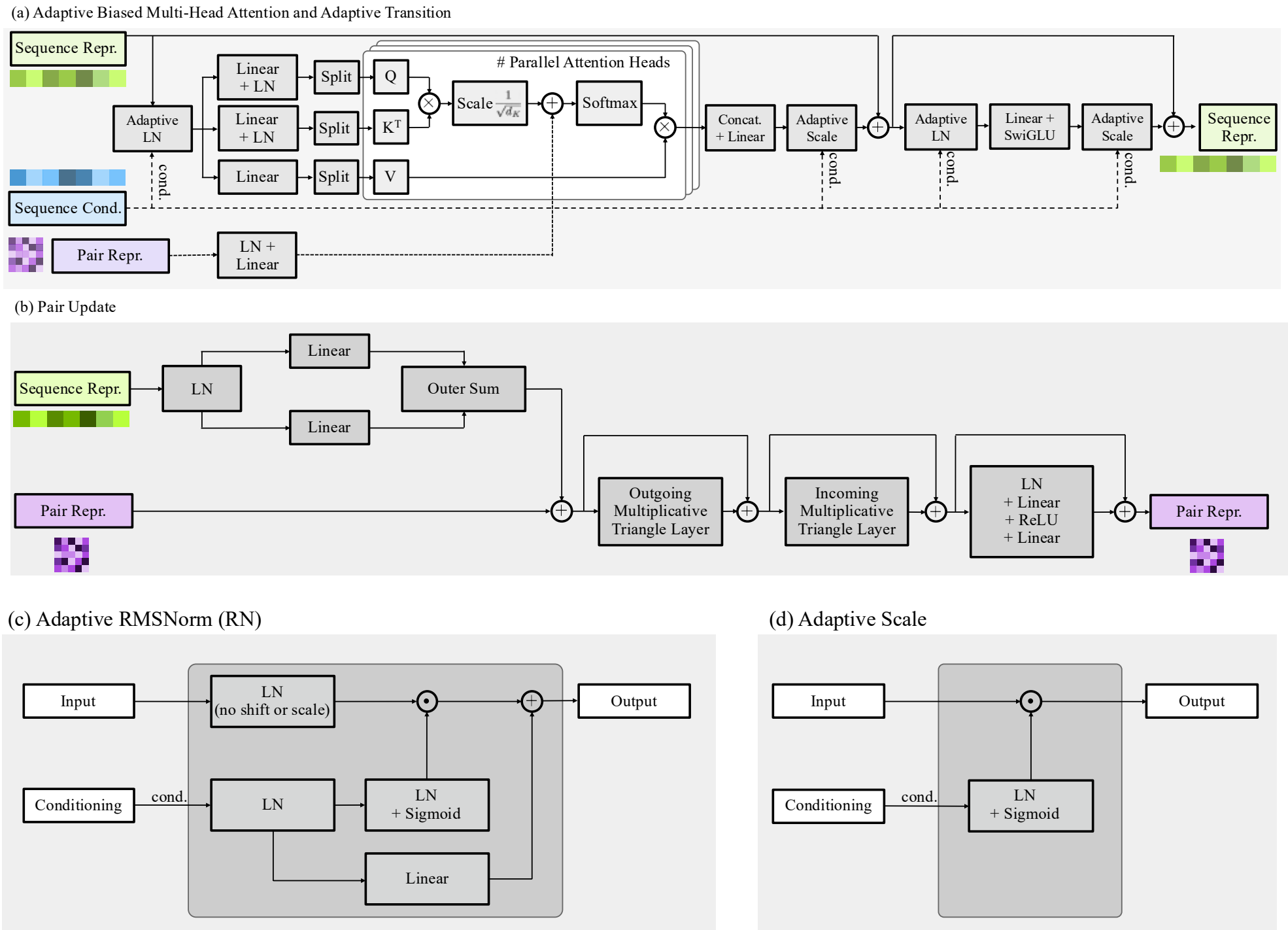

Fig. S11 Architecture of one conditional sequence–pair transformer block. a, Adaptive pair-biased multi-head self-attention uses the pair representation to add a learned bias to the attention logits. Sequence conditioning modulates the normalization and residual scaling in both the attention and SwiGLU transition sublayers. **b**, The pair representation is updated from normalized sequence states through two learned projections and an elementwise product, followed by outgoing and incoming multiplicative triangle layers and a pair transition. Residual connections surround each pair update. **c**, Adaptive normalization uses the conditioning representation to generate a sigmoid gate and an additive shift for normalized activations. **d**, Adaptive scaling gates a residual branch using a sigmoid function of the normalized conditioning representation. In the diagram, RN denotes the base normalization operator, solid plus signs denote residual additions, and dashed connections carry conditioning signals or pair-derived attention biases.

bioRxiv preprint doi: https://doi.org/10.64898/2026.09.12.751118; this version posted September 18, 2026. The copyright holder for this preprint (which was not certified by peer review) is the author/funder, who has granted bioRxiv a license to display the preprint in perpetuity. It is made available under a CC-BY 4.0 International license.

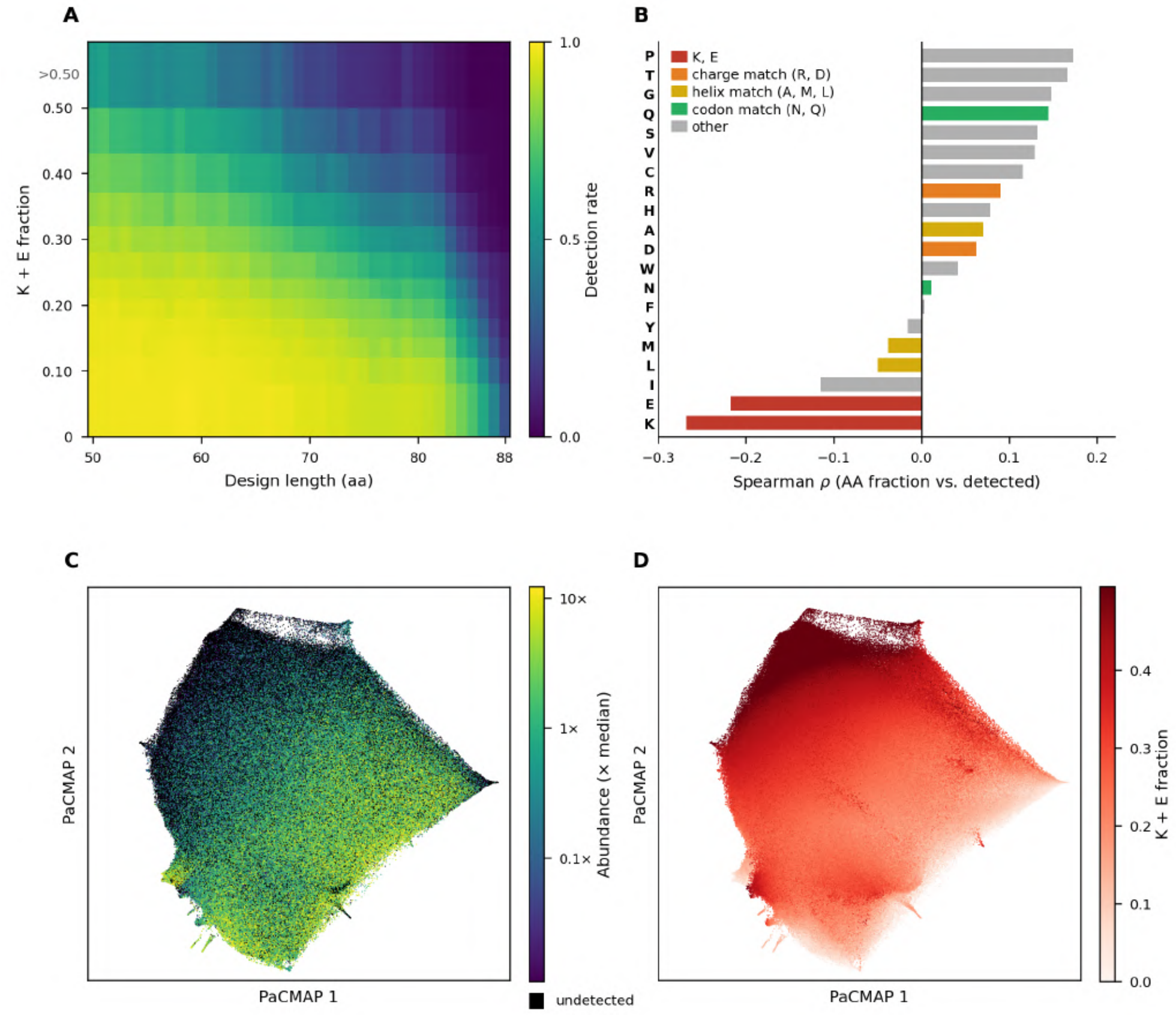

Fig. S12 Lysine and glutamate content correlates with loss from the phage input pool. **a**, Detection rate as a function of design length and combined K+E fraction across 1.4 million *de novo* designs in four length-stratified libraries. **b**, Spearman correlation between per-residue fractional composition and detection. **c**, PaCMAP embedding of all designs, highlighting undetected sequences and relative abundance among detected sequences. **d**, Same embedding colored by K+E fraction.

bioRxiv preprint doi: https://doi.org/10.64898/2026.09.12.751118; this version posted September 18, 2026. The copyright holder for this preprint (which was not certified by peer review) is the author/funder, who has granted bioRxiv a license to display the preprint in perpetuity. It is made available under a CC-BY 4.0 International license.

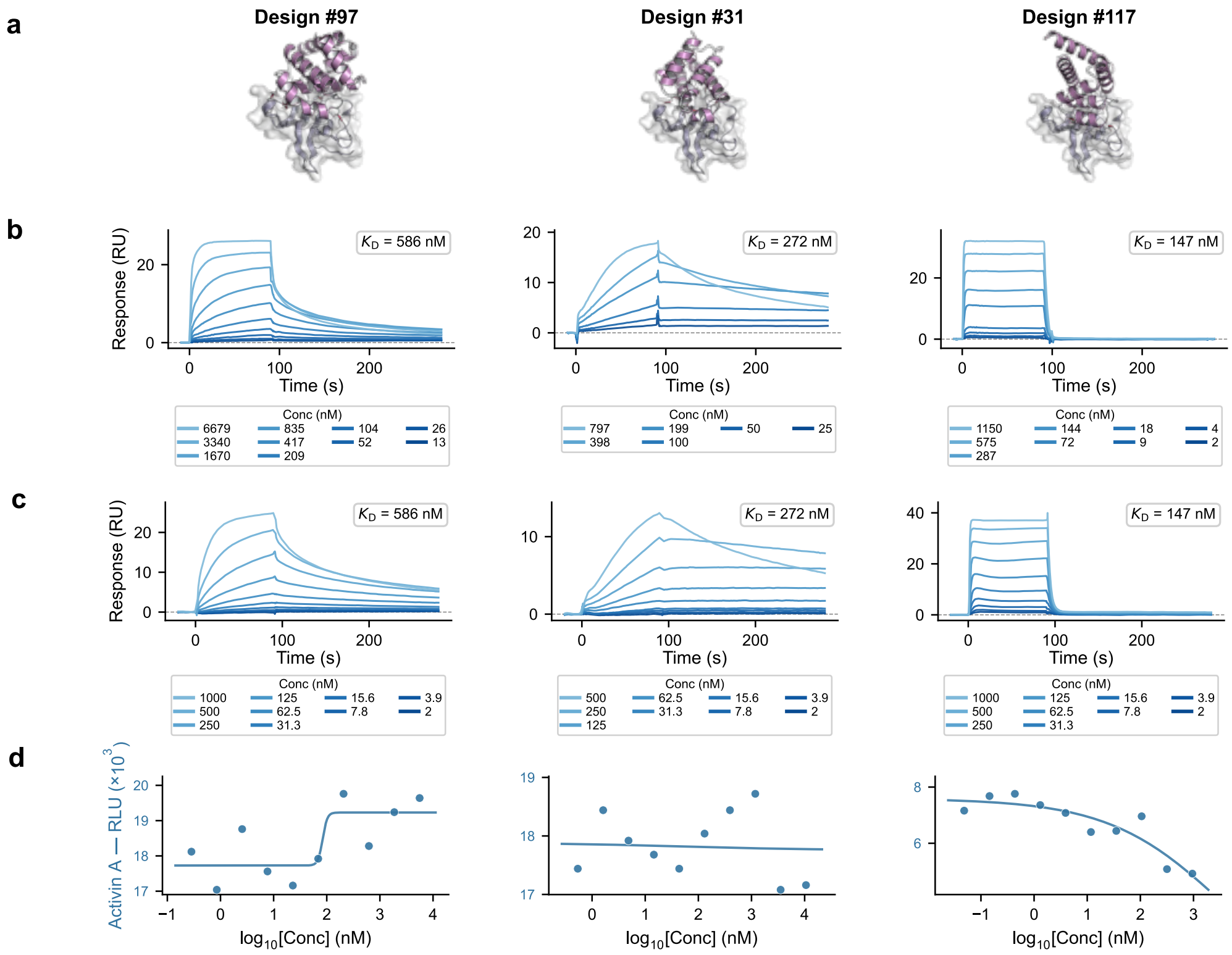

Fig. S13 Experimental characterization of three additional ActRIIA binder designs. Designs #97, #31 and #117 achieved sub-μM binding affinity but did not exhibit significant functional inhibition. **a**, Predicted complex structures of each binder (purple) engaged with the ActRIIA receptor (gray surface). **b**, SPR sensorgrams from the first independent measurement (raw traces only), with equilibrium K_D values indicated. **c**, SPR sensorgrams from the second independent measurement (raw traces only). **d**, Dose-response curves from the Smad2/3-responsive luciferase reporter assay under Activin A stimulation (20 ng/mL). Despite nanomolar-to-sub-μM affinity for ActRIIA, these designs did not produce measurable functional inhibition at the tested concentrations, suggesting that binding orientation and/or epitope overlap was insufficient to block native ligand signaling.

bioRxiv preprint doi: https://doi.org/10.64898/2026.09.12.751118; this version posted September 18, 2026. The copyright holder for this preprint (which was not certified by peer review) is the author/funder, who has granted bioRxiv a license to display the preprint in perpetuity. It is made available under a CC-BY 4.0 International license.

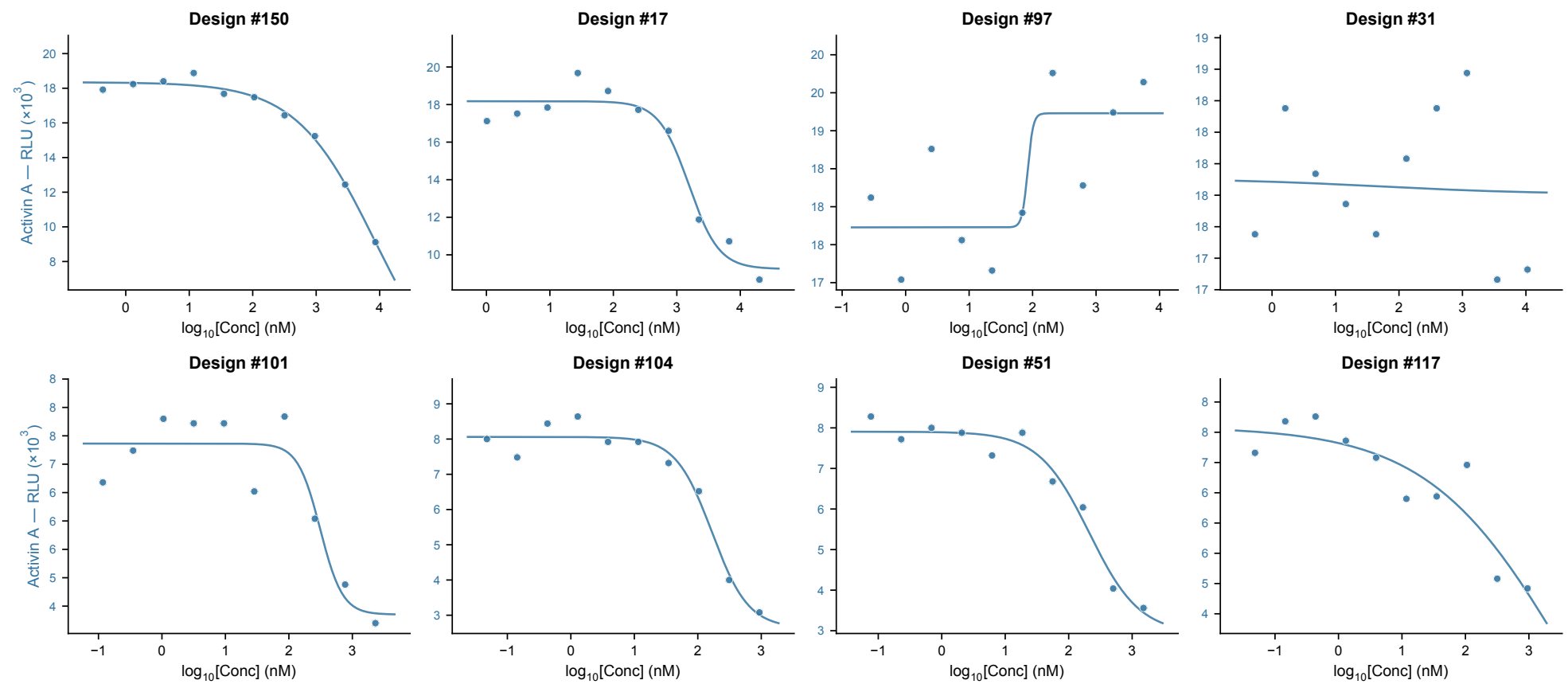

Fig. S14 Activin A functional assay across all eight ActRIIA binder designs. Dose-response curves from the Smad2/3 luciferase reporter assay are shown for all eight designs tested in this campaign (blue curves). Luminescence values are plotted against $\log_{10}$ concentration (nM). This panel complements the focused main-text figure by showing the full set of experimentally characterized designs under Activin A stimulation conditions.

bioRxiv preprint doi: https://doi.org/10.64898/2026.09.12.751118; this version posted September 18, 2026. The copyright holder for this preprint (which was not certified by peer review) is the author/funder, who has granted bioRxiv a license to display the preprint in perpetuity. It is made available under a CC-BY 4.0 International license.

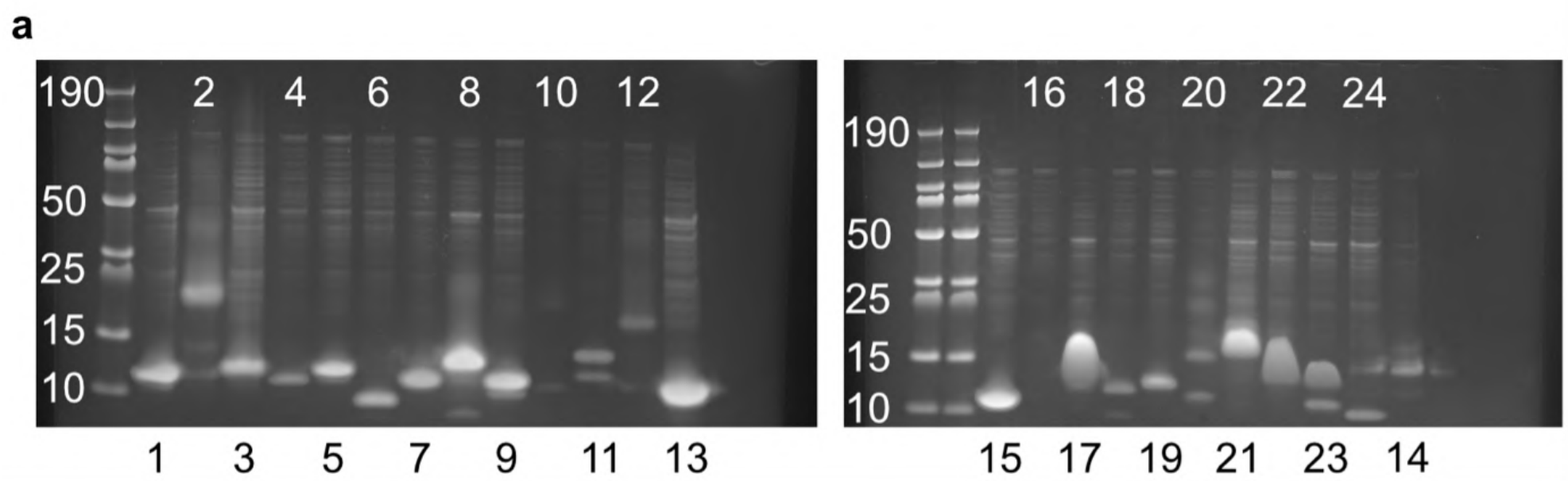

Fig. S15 Expression of the initial carbohydrate-binder candidates. SDS-PAGE analysis of the 24 candidates expressed in *E. coli*. Lanes S1–S24 correspond to the expression-test panel; molecular-weight markers (kDa) are shown. Twenty-three designs advanced to the binding screen reported in the main text.

bioRxiv preprint doi: https://doi.org/10.64898/2026.09.12.751118; this version posted September 18, 2026. The copyright holder for this preprint (which was not certified by peer review) is the author/funder, who has granted bioRxiv a license to display the preprint in perpetuity. It is made available under a CC-BY 4.0 International license.

S9 Supplementary tables

The following tables supplement the main text and Supplementary Methods.

bioRxiv preprint doi: https://doi.org/10.64898/2026.09.12.751118; this version posted September 18, 2026. The copyright holder for this preprint (which was not certified by peer review) is the author/funder, who has granted bioRxiv a license to display the preprint in perpetuity. It is made available under a CC-BY 4.0 International license.

Table S1 Temperature ablation for Proteina-Complexa (self-generated sequences) on baseline solvable targets ($n = 83$). On-target hit counts, specificity (specific/all on-target), and target coverage across backbone and local latent temperature combinations. New and lost columns indicate targets gained or lost relative to the default (0.1, 0.1) setting; unique denotes targets solved exclusively by that condition and no other.

**Temperature**	**On-target**			**Targets**				**# Seqs.**
(backbone, local latent)	**All**	**Specific**	**Specificity**	**Solved**	**New**	**Lost**	**Unique**	
(0.1, 0.1)	**702**	**607**	**86.5%**	35	–	–	4	49,263
(0.1, 0.4)	<u>495</u>	<u>419</u>	<u>84.6%</u>	34	+12	−13	6	45,255
(0.4, 0.1)	492	412	83.7%	30	+6	−11	3	45,904
(0.4, 0.4)	318	232	73.0%	30	+7	−12	4	49,697

bioRxiv preprint doi: https://doi.org/10.64898/2026.09.12.751118; this version posted September 18, 2026. The copyright holder for this preprint (which was not certified by peer review) is the author/funder, who has granted bioRxiv a license to display the preprint in perpetuity. It is made available under a CC-BY 4.0 International license.

Table S2 Properties of selected PAK1 mini-protein binder candidates. NGS $\log_2$ FC represents the $\log_2$ fold-change in read frequency relative to the input library after kanamycin selection. AF2 ipTM+pTM is the combined score from AlphaFold2 Multimer (best of 25 models).

**Binder**	**Length (aa)**	**NGS** $\log_2$ FC	**AF2 ipTM+pTM**	**Amino acid sequence**
Pk3	57	1.83	0.848	IVAQVIYHRLSPELRQEFEEKYKGNKSNAKLF AFARQKDPSLTQESVARVLFRQIVA
Pk4	51	1.05	0.765	IFKALARKLLPPEVYEKIKDSGPVTILNAISQ YTGKSKEALARPLFRQVVA
Pk5	52	1.73	0.626	FIEIIVKTKFRKEYEAELEKGKSPFQAANTVL AKKGLSRESVARPLFRQLVA
Pk6	55	−0.20	0.538	FRFHIIKRKAQKNPQYSHLSDAELLALARKLS LLSSHPLAPTEKEYRTLFRQIVA

bioRxiv preprint doi: https://doi.org/10.64898/2026.09.12.751118; this version posted September 18, 2026. The copyright holder for this preprint (which was not certified by peer review) is the author/funder, who has granted bioRxiv a license to display the preprint in perpetuity. It is made available under a CC-BY 4.0 International license.

Table S3 Predicted CK1δ peptide binders. All peptides were synthesized with an N-terminal biotinylated lysine followed by two glycine spacers.

Name	Length (aa)	Sequence
CK1	25	YTEAMDLVRKGIYRREEVLAQYGEK
CK2	27	EEQYKVSLQYAKEKKLEEYIKSINQLI
CK3	26	LSLAEYLVKHLKLPKNLAEKIASSMN
CK4	26	SLSEATIQLIAKQLGVDASQLEQLLS
CK5	25	DAVYLKSILQSGDLLRLKEYLLSLN
CK6	26	NFLEDGLSPSTALSFAIEKLCIQYGV
CK7	25	KQATEDRIRQEAEQKAAQERQQLAA
CK8	26	NLQEIKQQYREKEQQLQQEQQQRQQQ
CK9	28	FPCSYPGCGKTFTSSSSLRNHMKTHSRS
CK10	30	EEYKFELERLKKGELSPTEITLLEENLKRA
CK11	26	ELLKENPDLSVDFIRHQLSLLRSVGV
CK12	26	SLAEELFLRKLVDVHTANLISEKLTE
CK13	25	QLTSYQLEFMKKHGLTTEEELTAFM
CK14	25	YTSVYSSIYGPCPSCDEEERRRFGL
CK15	29	DLSDLSLGSLELIARLTGTNLSDLLAQRK
CK16	28	SDIKKLREFFTEKGYNPSDNLLELMVKL
CK17	30	GGASDEFLARLLGVSVEEIKRRRSLAASKK
CK18	30	EFAKNRQLLEEDLKRRGFSPEEISYRLSFV

bioRxiv preprint doi: https://doi.org/10.64898/2026.09.12.751118; this version posted September 18, 2026. The copyright holder for this preprint (which was not certified by peer review) is the author/funder, who has granted bioRxiv a license to display the preprint in perpetuity. It is made available under a CC-BY 4.0 International license.

Table S4: **Architecture and training hyperparameters for Proteína-Complexa.** Values are reproduced from Appendix G and Table 14 of ref. [18]. Protein–protein denotes the model used for protein-target binder generation, and ligand–protein the model used for small-molecule targets. All GPU counts refer to NVIDIA A100-80GB devices. N/A, not applicable.

**Hyperparameter**	**Protein–protein**	**Ligand–protein**
Partially latent flow-matching architecture		
Sequence representation dimension	768	768
Sequence-conditioning dimension	256	256
t sinusoidal-encoding dimension	256	256
Index sinusoidal-encoding dimension	256	256
Fold-class-conditioning dimension	0	0
Pair-representation dimension	256	256
Sequence-separation dimension	128	128
Pair-distance dimension, $\mathbf{x} * t$	30	30
Pair-distance dimension, $\widehat{\mathbf{x}}(\mathbf{x} * t)$	30	30
Pair-distance minimum (Å)	1	1
Pair-distance maximum (Å)	30	30
Attention heads	12	12
Transformer layers	14	14
Trainable parameters	159M	159M
Variational autoencoder architecture		
Encoder/decoder sequence-representation dimension	768	768
Encoder/decoder attention heads	12	12
Encoder/decoder transformer layers	12	12
Encoder/decoder sequence-conditioning dimension	128	128
Encoder/decoder index sinusoidal-encoding dimension	128	128

Continued on next page

bioRxiv preprint doi: https://doi.org/10.64898/2026.09.12.751118; this version posted September 18, 2026. The copyright holder for this preprint (which was not certified by peer review) is the author/funder, who has granted bioRxiv a license to display the preprint in perpetuity. It is made available under a CC-BY 4.0 International license.

Table S4: **Architecture and training hyperparameters for Proteína-Complexa** (continued).

**Hyperparameter**	**Protein–protein**	**Ligand–protein**
Encoder/decoder pair-representation dimension	256	256
Latent dimension	8	8
Trainable parameters	256M	256M
VAE training on AFDB		
Dataset	AFDB monomers	AFDB monomers
Maximum sequence length	256	512
Training steps	500k	140k
Batch size per GPU	14	5
Learning rate	10^{-4}	10^{-4}
Optimizer	Adam	Adam
GPUs	16	32
VAE fine-tuning on PDB		
Dataset	PDB	N/A
Training steps	40k	N/A
Batch size per GPU	12	N/A
Learning rate	10^{-4}	N/A
Optimizer	Adam	N/A
GPUs	16	N/A
Partially latent flow-model pretraining		
Dataset	AFDB monomers	AFDB monomers
Maximum sequence length	256	512
Training steps	540k	270k
Batch size per GPU	12	5
Learning rate	10^{-4}	10^{-4}
Optimizer	Adam	Adam
GPUs	32	48
Partially latent flow-model fine-tuning		
Dataset	Teddymer + PDB	PLINDER + AFDB monomers
Training steps	290k	60k
Target dropout	0%	50%
Low-rank adaptation (LoRA)	No	Yes
LoRA rank	N/A	32
LoRA α	N/A	64
Batch size per GPU	6	5
Learning rate	10^{-4}	10^{-4}
Optimizer	Adam	Adam
GPUs	96	96

bioRxiv preprint doi: https://doi.org/10.64898/2026.09.12.751118; this version posted September 18, 2026. The copyright holder for this preprint (which was not certified by peer review) is the author/funder, who has granted bioRxiv a license to display the preprint in perpetuity. It is made available under a CC-BY 4.0 International license.

Table S5: Reward functions and downstream selection criteria used in this study. The table consolidates the campaign-specific definitions reported in the main text and Methods. Unless stated otherwise, beam search used 400 denoising steps, beam width $N = 4$, branch factor $L = 4$ and update interval $K = 100$; Feynman–Kac steering used inverse temperature $\beta = 10$ [18].

Campaign or task	Search procedure	Reward used during search	Downstream success or selection criteria
Designed monomers	Generation followed by filtering	None since pure generative model benchmarking	AlphaFold2 single-sequence mean pLDDT > 90 and backbone RMSD < 2 Å to the design model
PDGFR and PD-L1	Beam search	AlphaFold2-Multimer interface predicted aligned error (interface pAE)	Physicochemical and confidence filtering, TM-score clustering at 0.8, then OpenFold3 and Boltz-2 monomer validation with pLDDT > 80 and RMSD < 2 Å
Nipah virus G, *de novo* and scaffold re-engineering	Beam search	Multi-objective combination of Boltz-2 maximum interface score, shape complementarity, interface hydrophobicity and hydrogen-bond satisfaction	Top 10 de novo and top 10 scaffold reengineering variants were selected ipAE, TMol hydrogen bonds, shape complementarity and visual inspection for key interactions
Massive-scale 127-target benchmark	Beam search	AlphaFold2-Multimer interface pAE	Minimum interface pAE < 2, complex pLDDT > 0.8, interface pTM > 0.7 and fewer than five C_α clashes; diversity selection cycled rankings across minimum interface pAE, interface pTM, minimum interface pSAE and random selection
ActRIIA	Beam search, Feynman–Kac steering or Monte Carlo tree search across seven rounds	AlphaFold2-Multimer interface pAE	Ranking by the averaged score followed by Foldseek scaffold clustering and experimental surface-plasmon-resonance testing
PAK1 mini-proteins	Beam search	AlphaFold2-Multimer interface pAE (lower is better)	Fifty best selected candidates by ipAE were tested

Continued on next page

bioRxiv preprint doi: https://doi.org/10.64898/2026.09.12.751118; this version posted September 18, 2026. The copyright holder for this preprint (which was not certified by peer review) is the author/funder, who has granted bioRxiv a license to display the preprint in perpetuity. It is made available under a CC-BY 4.0 International license.

Table S5: Reward functions and downstream selection criteria used in this study (continued).

**Campaign or task**	**Search procedure**	**Reward used during search**	**Downstream success or selection criteria**
CK1δ peptides	Beam Search	AlphaFold2-Multimer interface pAE	18 best candidates were selected by ipAE
Carbohydrate antigen	Beam search	Pocket burial, physics-based Tmol hydrogen-bond energies and RosettaFold3 interface confidence; after each search step the RosettaFold3-predicted sugar pose replaced the preceding pose	RosettaFold3 minimum interface pAE < 2, binder RMSD < 2 Å and ligand RMSD < 5 Å

bioRxiv preprint doi: https://doi.org/10.64898/2026.09.12.751118; this version posted September 18, 2026. The copyright holder for this preprint (which was not certified by peer review) is the author/funder, who has granted bioRxiv a license to display the preprint in perpetuity. It is made available under a CC-BY 4.0 International license.

Table S6: List of commercially sourced proteins used in the Manifold Bio large-scale all-by-all binder screen.

Target_UniProtID	Target	Catalog No.	Vendor
ACE2_Q9BYF1	ACE2	10108-H08H	Sino Biological
ADAM10_O14672	ADAM10	936-AD	R&D Systems
ALCAM_Q13740	ALCAM	10045-H08H	Sino Biological
ANTXR1_Q9H6X2	ANTXR1	13367-H08H	Sino Biological
AOC3_Q16853	AOC3	3957-AO	R&D Systems
BACE2_Q9Y5Z0	BACE2	10783-H08H	Sino Biological
BCAM_P50895	BCAM	10238-H08H	Sino Biological
Bet v1_Q42499	Bet v1	NA-BV1-1	In Bio
BGN_P21810	BGN	10447-H08H	Sino Biological
CA12_O43570	CA12	10617-H08H	Sino Biological
CCR5_P51681	CCR5	13022-H91H-NA	Sino Biological
CD200_P41217	CD200	10886-H08H	Sino Biological
CD46_P15529	CD46	12239-H08H	Sino Biological
CD47_Q08722	CD47	12283-H08H	Sino Biological
CD55_P08174	CD55	10101-H08H	Sino Biological
CD59_P13987	CD59	12474-H08H	Sino Biological
CD81_P60033	CD81	14244-H07H	Sino Biological
CEACAM1_P13688	CEACAM1	10822-H08H	Sino Biological
CEACAM5_P06731	CEACAM5	11077-H08H	Sino Biological
CEACAM6_P40199	CEACAM6	10823-H08H	Sino Biological
CEMIP2_Q9UHN6	CEMIP2	CSB-EP023791HU	Cusabio
CLMP_Q9H6B4	CLMP	5019-AM	R&D Systems
CLU_P10909	CLU	11297-H08H1	Sino Biological
CNTFR_P26992	CNTFR	11012-H08B	Sino Biological
CR1_P17927	CR1	5748-CD	R&D Systems
CSPG4_Q6UVK1	CSPG4	2585-PG	R&D Systems
CXADR_P78310	CXADR	10799-H08H	Sino Biological
CXCR3_P49682	CXCR3	12979-H95H-NA	Sino Biological
CXCR4_P61073	CXCR4	11325-H95H-NA	Sino Biological
DCN_P07585	DCN	10189-H08H	Sino Biological
DPP4_P27487	DPP4	10688-H08H	Sino Biological
EFNB1_P98172	EFNB1	10894-H08H	Sino Biological
ENPEP_Q07075	ENPEP	10554-H07B	Sino Biological
ENTPD1_P49961	ENTPD1	16020-H08B	Sino Biological
FcRn_P55899	FcRn	CT009-H08H	Sino Biological
FLRT2_O43155	FLRT2	11296-H08H	Sino Biological
FMOD_Q06828	FMOD	11514-H02H	Sino Biological
FN1_P02751	FN1	10314-H08H	Sino Biological
HER2_P04626	HER2	10004-H08H	Sino Biological
HLA-B_P01889	HLA-B	ab225646-100ug	Abcam
HLA-E_P13747	HLA-E	HL0-H82E7-25ug	Acro
HYAL2_Q12891	HYAL2	11012-GH	R&D Systems
ICAM1_P05362	ICAM1	10346-H08H	Sino Biological
IFNAR2_P48551	IFNAR2	10359-H08H	Sino Biological

Continued on next page

bioRxiv preprint doi: https://doi.org/10.64898/2026.09.12.751118; this version posted September 18, 2026. The copyright holder for this preprint (which was not certified by peer review) is the author/funder, who has granted bioRxiv a license to display the preprint in perpetuity. It is made available under a CC-BY 4.0 International license.

Table S6 – *continued from previous page*

Target_UniProtID	Target	Catalog No.	Vendor
IGSF8_Q969P0	IGSF8	13435-H08H	Sino Biological
IL-13_P35225	IL-13	10369-H08H	Sino Biological
IL4R_P24394	IL4R	10402-H08H	Sino Biological
ITGA6_P23229	ITGA6	CT069-H2508H	Sino Biological
JAM2_P57087	JAM2	1074-VJ	R&D Systems
KIRREL1_Q96J84	KIRREL1	15752-H08H1	Sino Biological
LTBP4_Q8N2S1	LTBP4	CSB-EP850788HU	Cusabio
MCAM_P43121	MCAM	10115-H08H	Sino Biological
MRC2_Q9UBG0	MRC2	10483-EN	R&D Systems
NECTIN2_Q92692	NECTIN2	10005-H08H	Sino Biological
NEO1_Q92859	NEO1	8607-NE-050	R&D Systems
NRG3_P56975	NRG3	16071-H08H	Sino Biological
NTM_Q9P121	NTM	15169-H08H	Sino Biological
NTRK2_Q16620	NTRK2	10047-H08H	Sino Biological
PD-1_Q15116	PD-1	10377-H08H	Sino Biological
PD-L1_Q9NZQ7	PD-L1	10084-H08H	Sino Biological
PDGFRA_P16234	PDGFRA	10556-H08H	Sino Biological
POSTN_Q15063	POSTN	10299-H08H	Sino Biological
PSG1_P11464	PSG1	15378-H08H	Sino Biological
RECK_O95980	RECK	10309-RE	R&D Systems
SEMA4D_Q92854	SEMA4D	11825-H08H	Sino Biological
SEMA7A_O75326	SEMA7A	2068-S7	R&D Systems
SERPINA3_P01011	SERPINA3	10307-H08H	Sino Biological
SIRPA_P78324	SIRPA	30014-H08H	Sino Biological
SLIT2_O94813	SLIT2	11967-H08H	Sino Biological
SLIT3_O75094	SLIT3	9067-SL	R&D Systems
THBD_P07204	THBD	11864-H08H	Sino Biological
THSD1_Q9NS62	THSD1	IT-010-017p	Immune Technology
TSPAN13_O95857	TSPAN13	11551-TS	R&D Systems
TSPAN14_Q8NG11	TSPAN14	11552-TS	R&D Systems
TSPAN31_Q12999	TSPAN31	11553-TS	R&D Systems
TSPAN8_P19075	TSPAN8	15683-H07H	Sino Biological
VSTM4_Q8IW00	VSTM4	2086-VT	R&D Systems
Additional targets (short-form identifiers)			
AHSP	AHSP	14391-HNAE	Sino Biological
AMBP	AMBP	13141-H05H1	Sino Biological
CALR	CALR	13539-H02H	Sino Biological
CD226	CD226	10565-H02H	Sino Biological
CEAM6	CEAM6	10823-H08H-B	Sino Biological
CRTAM	CRTAM	11975-H08H-B	Sino Biological
CSF1	CSF1	11792-H02H	Sino Biological
DAF	DAF	10101-H02H	Sino Biological
EFNA1	EFNA1	10882-H02H	Sino Biological
EFNA5	EFNA5	10192-H02H	Sino Biological
EFNB2	EFNB2	10881-HCCH	Sino Biological

Continued on next page

bioRxiv preprint doi: https://doi.org/10.64898/2026.09.12.751118; this version posted September 18, 2026. The copyright holder for this preprint (which was not certified by peer review) is the author/funder, who has granted bioRxiv a license to display the preprint in perpetuity. It is made available under a CC-BY 4.0 International license.

Table S6 – *continued from previous page*

Target_UniProtID	Target	Catalog No.	Vendor
EPCR	EPCR	13320-H02H	Sino Biological
FCG3B	FCG3B	11046-H27H-B	Sino Biological
FGFR1	FGFR1	10616-H02H	Sino Biological
FST	FST	10685-H02H	Sino Biological
HDAC8	HDAC8	10864-H09B	Sino Biological
IL1R1	IL1R1	10126-H02H	Sino Biological
IL20	IL20	13060-HNAE	Sino Biological
IL3	IL3	11858-HNAE	Sino Biological
LEP	LEP	10221-HNAE	Sino Biological
MK08	MK08	10795-H09B	Sino Biological
MMP2	MMP2	10082-HNAH	Sino Biological
NECT1	NECT1	11611-H02H	Sino Biological
PARVA	PARVA	13919-H09E	Sino Biological
PD1L2	PD1L2	10292-H02H	Sino Biological
PIN1	PIN1	10282-HNCE	Sino Biological
PTN2	PTN2	10570-HNCB	Sino Biological
RNF43	RNF43	16108-H02H	Sino Biological
RSPO1	RSPO1	11083-HNAS	Sino Biological
S10A4	S10A4	10185-H01H	Sino Biological
SAE1	SAE1	13921-HNCB	Sino Biological
SORCN	SORCN	14547-HNCB	Sino Biological
STC2	STC2	13653-H02H	Sino Biological
SYUA	SYUA	12093-HNAE	Sino Biological
TACT	TACT	11202-H08H-B	Sino Biological
TF65	TF65	12054-H09E	Sino Biological
TNFL4	TNFL4	13127-H04H	Sino Biological
TNFL9	TNFL9	15693-H01H	Sino Biological
UBC9	UBC9	13205-HNCE	Sino Biological
UBE2B	UBE2B	51243-MNCE	Sino Biological
UBP7	UBP7	11681-HNCB	Sino Biological
VATF	VATF	15605-H09E	Sino Biological
XIAP	XIAP	10606-H17E	Sino Biological
Benchmark targets			
BHRF1_2wh6	BHRF1	LS-G64115-20	LS Bio
SC2RBD_6m0j	SC2RBD	40592-V08H-100	Sino Biological
IL-7RA_3di3	IL-7RA	10975-H02H-100	Sino Biological
PD-L1_5o45	PD-L1	10084-H02H-100	Sino Biological
TrkA_1www	TrkA	11073-H08H-100	Sino Biological
Insulin_4zxb	Insulin	11038-HNAY-100	Sino Biological
H1N1_5vli	H1N1	11684-V08H-100	Sino Biological
VEGF-A_1bj1	VEGF_A	11066-HNAH-20	Sino Biological
IL-17A_4hsa	IL-17A	12047-HNAS-100	Sino Biological
TNFa_1tnf	TNFa	10602-H08H-50	Sino Biological

bioRxiv preprint doi: https://doi.org/10.64898/2026.09.12.751118; this version posted September 18, 2026. The copyright holder for this preprint (which was not certified by peer review) is the author/funder, who has granted bioRxiv a license to display the preprint in perpetuity. It is made available under a CC-BY 4.0 International license.

Table S7 Data collection and refinement statistics for LP46.

	**LP46**
**PDB ID**	32GJ
**Data collection**	
Synchrotron, beamline	HZB, BESSYII (MX 14.1)
Wavelength (Å)	0.9184
Space group	$P6_1$
a, b, c (Å)	164.564, 164.564, 71.344
α, β, γ (°)	90.00, 90.00, 120.00
Resolution (Å)*	47.506 – 2.948 (3.346 – 2.948)
Directional diffraction limits (Å)*	3.535, 3.535, 2.817
Total reflections*	327788 (29129)
Total unique*	16020 (1332)
R_{merge}*	0.365 (2.221)
R_{meas}*	0.374 (2.273)
Mean $I/\sigma(I)$*	10.0 (1.7)
Completeness (ellipsoidal, %)*	94.6 (77.6)
Completeness (spherical, %)*	68.2 (18.1)
Multiplicity*	20.5 (21.9)
$CC_{1/2}$*	0.996 (0.638)
**Refinement**	
Resolution (Å)#	47.51 – 2.95 (3.04 – 2.95)
Reflections used in refinement#	16013 (136)
R_{work}#	0.2146 (0.3594)
R_{free}#	0.2452 (0.3415)
Number of non-hydrogen atoms#	6051
macromolecules	6051
ligands	0
solvent	0
Protein residues	794
RMS deviations (bonds)#	0.003
RMS deviations (angles)#	0.59
Ramachandran favored (%)#	97.22
Ramachandran allowed (%)#	2.78
Ramachandran outliers (%)#	0.00
Rotamer outliers (%)#	0.54
Clashscore#	5.07
Average B-factor#	57.50
macromolecules	57.50
MolProbity Score#	1.41

Criteria used in determination for diffraction limits*: local($I/\sigma I$) $\geq$ 1.20.
*As reported by STARANISO; directional limits (Å) along principal axes of ellipsoid fitted to diffraction cut-off surface; axis vectors: a^* [1.00, 0.00, 1.00], b^* [0.00, 1.00, 0.00], c^* [0.00, 0.00, 1.00].
#As reported by `phenix.table_one`.

bioRxiv preprint doi: https://doi.org/10.64898/2026.09.12.751118; this version posted September 18, 2026. The copyright holder for this preprint (which was not certified by peer review) is the author/funder, who has granted bioRxiv a license to display the preprint in perpetuity. It is made available under a CC-BY 4.0 International license.

Table S8 Evaluation of hotspot performance for binders to PDGFR and PDL1 targets.

**Input ID**	**Hotspots**	**Percent Binders**	**Best** $-\log(K_d)$
NN-PDGFR-1	A139, A77, A82, A11, A116, A80, A142, A8, A134	73.5%	9.61
NN-PDGFR-2	A77, A82, A11, A80, A8	77.5%	9.75
NN-PDGFR-3	A139, A116, A142, A134	31.3%	7.42
NN-PDGFR-5	A139, A77	58.5%	9.24
NN-PDGFR-6	A139, A77, A82	71.1%	10.03
NN-PDL1-1	A39, A98, A51, A108, A49, A106, A59, A37, A32	5.5%	6.97
NN-PDL1-2	A39	5.3%	6.10
NN-PDL1-3	A39, A98	9.1%	6.33
NN-PDL1-4	A39, A98, A51	17.9%	6.75
NN-PDL1-5	A98, A108, A106	8.3%	6.03
NN-PDL1-6	A39, A51, A49, A37	11.1%	5.02
NN-PDL1-7	A39, A98, A108, A106	13.9%	6.91

bioRxiv preprint doi: https://doi.org/10.64898/2026.09.12.751118; this version posted September 18, 2026. The copyright holder for this preprint (which was not certified by peer review) is the author/funder, who has granted bioRxiv a license to display the preprint in perpetuity. It is made available under a CC-BY 4.0 International license.

Table S9 Expression and purification yields for the 200 ActRIIA binder designs. Soluble expression yields are reported after the first nickel-affinity purification step for both His-tagged (tagged) and TEV-cleaved (tagless) constructs. Of the 200 designs, 192 expressed solubly and 110 achieved yields exceeding 50 mg/L in the tagged format. Tag removal by TEV protease was unsuccessful for 22 constructs.

**Yield (after 1st nickel)**	**Tagged Constructs**	**Tagless**
No soluble expression	9	9
Tag can't be cleaved	–	22
<10 mg/L	2	59
10–20 mg/L	9	52
20–30 mg/L	12	36
30–50 mg/L	58	20
>50 mg/L	110	2
**Total**	**200**	**200**
